# Reducing stigma and discrimination associated with COVID-19: early stage pandemic rapid review and practical recommendations

**DOI:** 10.1017/S2045796021000056

**Published:** 2021-01-28

**Authors:** P. C. Gronholm, M. Nosé, W. H. van Brakel, J. Eaton, B. Ebenso, K. Fiekert, M. Milenova, C. Sunkel, C. Barbui, G. Thornicroft

**Affiliations:** 1 Health Service and Population Research Department, Centre for Global Mental Health and Centre for Implementation Science, Institute of Psychology, Psychiatry & Neuroscience, King's College London, London, UK; 2 WHO Collaborating Centre for Research and Training in Mental Health and Service Evaluation; Department of Neuroscience, Biomedicine and Movement Sciences; Section of Psychiatry, University of Verona, Verona, Italy; 3 NLR International, Amsterdam, Netherlands; 4 CBM Global, and Centre for Global Mental Health, London School of Hygiene and Tropical Medicine, Keppel Street, London, UK; 5 Leeds Institute of Health Sciences, University of Leeds, Leeds, UK; 6 KNCV Tuberculosis Foundation, The Hague, Netherlands; 7 Global Mental Health Peer Network, Johannesburg, Gauteng, South Africa

**Keywords:** Coronavirus, emergency response, health inequalities, public health

## Abstract

**Aims:**

To develop recommendations for strategies and interventions to reduce stigma and discrimination related to coronavirus disease 2019 (COVID-19), through reviewing and synthesising evidence in relation to COVID-19 and other disease outbreaks and infectious/stigmatised conditions from systematic reviews and primary studies and recommendations from additional materials.

**Methods:**

Rapid review, drawing on the World Health Organization's (WHO) methodology for developing interim guidelines during health emergencies. PubMed/MEDLINE, PsycINFO, Cochrane Central and Campbell Collaboration searched up to mid-April 2020. Searches were supplemented by reference-searching and expert recommendations. Searches were designed to identify: (1) systematic reviews (<10 years), or (2) primary intervention studies (no date limit) reporting evidence on anti-stigma interventions (in relation to COVID-19 or other infectious/stigmatised conditions) or (3) additional relevant materials. Data were extracted on population, intervention, outcome and results. These data were compiled into evidence summary tables and narrative overviews. Recommendations on strategies for COVID-19 stigma-reduction were developed using the WHO ‘Evidence to Decision’ framework approach. The review protocol was registered with PROSPERO (registration ID: CRD42020177677).

**Results:**

The searches identified a total of 4150 potentially relevant records, from which 12 systematic reviews and 29 additional articles were included. Overarching considerations and specific recommendations focus on: (1) language/words used in relation to COVID-19 and affected people; (2) media/journalistic practices; (3) public health interventions; (4) targeted public health interventions for key groups and (5) involving communities and key stakeholders.

**Conclusions:**

These recommendations represent the first consolidated evidence-based guidance on stigma and discrimination reduction in relation to COVID-19. Mitigating the impact of stigma is critical in reducing distress and negative experiences, and strengthening communities' resolve to work together during exceptional circumstances. Ultimately, reducing stigma helps addressing structural inequalities that drive marginalisation and exacerbate both health risks and the impact of stigma. Administrations and decision makers are urged to consider integrating these recommendations into the ongoing COVID-19 response.

## Introduction

A novel coronavirus pneumonia was first detected in Wuhan, China, in late 2019. This disease, officially named coronavirus disease 2019, or COVID-19, is caused by the virus SARS-CoV-2 (severe acute respiratory syndrome coronavirus 2). The World Health Organization (WHO) declared the disease outbreak a pandemic on 11 March 2020 (World Health Organization, 2020c). The emergence and rapid spread of this new viral diseases has caused confusion, anxiety and fear among the general public. As with other public health emergencies and perceived health threats, fear and anxiety about COVID-19 can result in stigma and discrimination towards people associated with the disease (Centres for Disease Control and Prevention, 2020).

Indeed, the COVID-19 pandemic has provoked stigmatisation and discriminatory behaviours against people who have, or might have, COVID-19 (World Health Organization, 2020b). The impact of stigma can be long-term, affecting the person beyond the acute phase of illness, continuing when people are no longer symptomatic, and when there is no longer a risk of others acquiring COVID-19 from the person. Stigma can also occur by association, meaning it affects people associated with COVID-19 through their work (e.g. health and social care workers), affiliation with a person who is unwell (e.g. caregivers, family members), and people of certain ethnic backgrounds or country of origin (due to public perception of places and populations amongst whom the virus is more common or where it occurred earlier) (World Health Organization, 2020b). These findings are in line with evidence that stigma has been a key concern in relation to previous comparable viral outbreaks and epidemics, for example severe acute respiratory syndrome (SARS), Middle-East respiratory syndrome (MERS) and the Ebola virus disease (Person *et al*., 2004; Fukuda *et al*., 2015; James *et al*., 2020).

Health-related stigma has been defined as a personal experience or social process characterised by exclusion, rejection, blame and devaluation, as a result of anticipating or experiencing negative social judgements due to a person or group being in association with a given health condition (Weiss and Ramakrishna, 2006). Given the complex ways in which stigma can manifest, to facilitate classification, stigma can been considered as composed of issues related to inaccurate knowledge (e.g. regarding what an illness is, how it is acquired), attitudes (e.g. stereotyped negative beliefs, or negative emotional reactions) and behaviours (e.g. discriminatory practices) (Thornicroft, 2006). Addressing stigma is important, as it can drive people to deny or hide the illness to avoid discrimination, to prevent or delay timely health care seeking, and can discourage people from adopting healthy behaviours (Stangl *et al*., 2019). Such barriers could contribute to more severe health problems, and greater difficulties in controlling the viral disease outbreak (Van Bortel *et al*., 2016). Stigma and discrimination often also affect the mental health of stigmatised people, which may itself worsen these negative outcomes. Stigmatisation can also lead to rejection, avoidance and social distancing of those who are feared (Stangl *et al*., 2019), potentially leading to further harm, such as making it harder for people to secure food or other basic necessities (BBC News 2020).

Understanding and countering the COVID-19 stigmatisation through interdisciplinary efforts has been highlighted as an urgent priority (Holmes *et al*., 2020; IASC Inter-Agency Standing Committee, 2020; Nature, 2020; World Health Organization, 2020a). In this context, the aim of this rapid review was to review evidence at the onset of the pandemic to develop timely, relevant and feasible recommendations for strategies and interventions to reduce stigma and discrimination related to COVID-19.

## Methods

### Search strategy and selection criteria

This rapid review draws on the methodology of the World Health Organization (WHO) ‘Health Emergency Interim Guidelines’ (Garritty *et al*., 2017; World Health Organization, 2017).

The review search strategy involves systematic database literature searches and expert consultations to identify relevant resources to inform guideline recommendation development.

Inclusion criteria of relevant articles for inclusion were systematic reviews of any type of primary intervention studies (from the past 10 years), any type of primary intervention studies (no date limit) reporting on the efficacy of anti-stigma interventions (all types of stigma) in relation to COVID-19 or in relation to the following highly infectious/stigmatised conditions: SARS, MERS, pandemic influenza, Ebola, tuberculosis, leprosy, HIV/AIDS or mental illness.

The outcome of interest was changes in stigma (any type; e.g. anticipated stigma, public stigma, self-stigma, structural stigma, stigma-by-association, perceived stigma), or changes in outcomes considered to be reflective of stigma (knowledge, attitudes, behaviours).

The target populations were: people experiencing stigma (e.g. people who are confirmed or suspected to have the condition, or who have recovered; people associated with the condition due to their work [e.g. healthcare workers], country of origin or ethnicity; or because of an affiliation with someone who is unwell [e.g. caregivers, family members]); or people who can act on stigma (e.g. general public, policy makers, people associated with the condition, or who have experience of the illness).

Database searches were conducted in PubMed/MEDLINE, PsycINFO, Cochrane Central and Campbell Collaboration, searched up to mid-April 2020. Search strategies were developed and ran separately in relation to each condition, and each database. In brief, we used a combination of search terms related to stigma (e.g. ‘stigma’, ‘discrimination’) AND the given condition (e.g. ‘COVID-19’, ‘2019-nCoV’, ‘SARS-CoV-2’). The searches followed a stepwise approach. First, the ‘stigma AND condition’ search was carried out with a limit to systematic reviews of any type of primary intervention studies reporting on the efficacy of anti-stigma interventions, published in the last 10 years. If this search yielded systematic reviews relevant for inclusion, no further searches were run. If no relevant systematic reviews were identified, then a second search was carried out for the ‘stigma AND condition’ with a limit to any type of intervention study reporting on the efficacy of an anti-stigma intervention. If no relevant studies were identified, a third search was carried out for ‘stigma AND condition’, with no limits. The search strategies are available in the online Supplementary Materials. Database searches were supplemented by reference searching for further literature for inclusion, and expert recommendations regarding potentially relevant articles.

Systematic reviews and primary studies identified through the first and second steps of the database search were considered as evidence for this review. Articles identified through the third step of the database search were considered as ‘additional materials’ relating to stigma-reduction and the condition. Within the additional materials, this review also considered related guidelines and recommendations from credible international and national public health organisations and intra- and inter-governmental organisations, sourced through expert recommendations.

The evidence and additional materials sourced through the searches were reviewed for inclusion following the WHO rapid advice guideline development principles (Garritty *et al*., 2017; World Health Organization, 2017). A key systematic review article (reflecting most recent, or most comprehensive evidence) was selected to provide evidence on stigma-reduction in relation to each condition (COVID-19, or other infectious/highly stigmatised condition); within conditions also considering evidence regarding type of stigma (e.g. self-stigma, public stigma, etc.), population (e.g. person living with illness, general public) and setting (e.g. healthcare), as relevant. Decisions regarding inclusion were made through discussion between the two lead authors and endorsed by the full review group (all authors).

The protocol for this review was registered on PROSPERO (ID: CRD42020177677).

### Data synthesis

The lead authors (PCG, MN) individually extracted information on population, intervention, outcome and results from the included evidence articles, and subsequently reviewed each other's extraction for accuracy. Extracted data were compiled into evidence summary tables, and reviewed for accuracy through group discussions. Methodological quality of included evidence was assessed using standard criteria in the AMSTAR-2 (Shea *et al*., 2017) framework, to aid recommendation development. Narrative overviews focused on information regarding stigma reduction were also made for each included evidence article and additional materials.

Following the WHO methodology for rapid development of recommendations, data from the included evidence article and additional materials were used to populate a GRADE Evidence to Decision (EtD) framework (Alonso-Coello *et al*., 2016). Here the review question (‘which interventions are effective in reducing COVID-19 related stigma?’) is considered in relation to various criteria (e.g. whether the problem addressed [COVID-19 stigma] is considered a priority, whether there is variability in how the outcome of interest [stigma] is valued, whether the favourable effects of an intervention [stigma reduction] outweigh potential undesirable effects; see online Supplementary Materials for further details). The data within the EtD framework are used to support the development of evidence-informed health system and public health recommendations and decisions, through a debate considering the available evidence and insights from additional materials as synthesised within the framework. Based on this discussion, the review group developed recommendations regarding effective strategies for stigma reduction in relation to COVID-19.

## Results

**Fig. 1.**
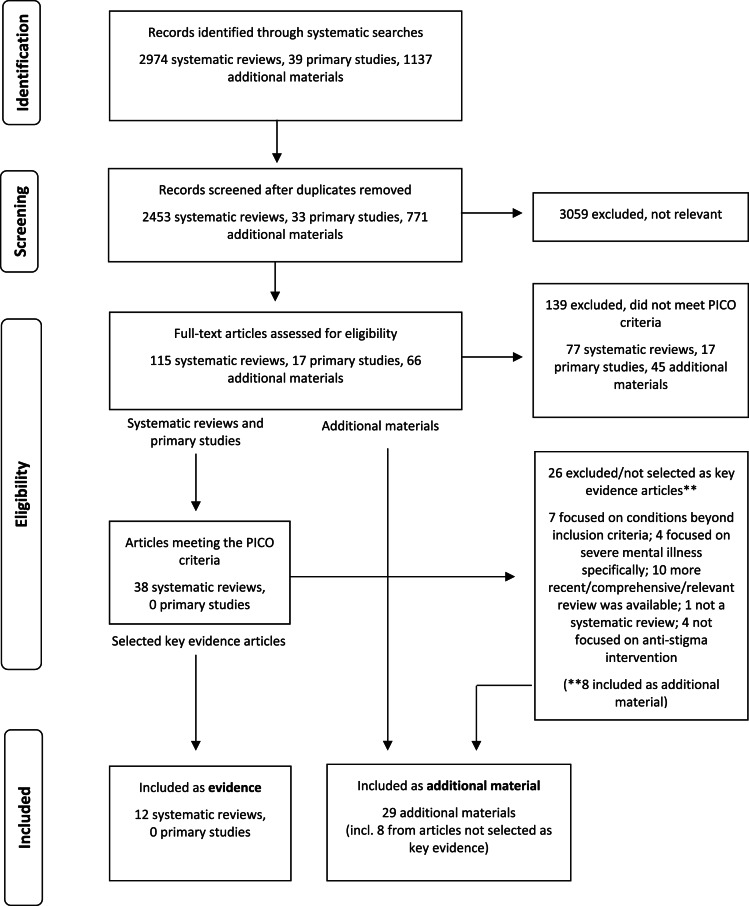
PRISMA flow diagram of the article selection process.

The searches identified a total of 4150 records, reflecting 2974 records of potentially relevant systematic reviews, 39 primary studies and 1137 articles representing additional materials. From these, 12 systematic reviews were selected as evidence for this review, no primary studies met the criteria, and 29 articles were included as additional materials. Figure 1 provides an overview of the article selection process.

The characteristics of the systematic reviews providing evidence for this review are presented in Table 1 (added details in online Supplementary Materials). These reviews reported on the effectiveness of anti-stigma interventions in relation to leprosy (Sermrittirong *et al*., 2014), tuberculosis (Sommerland *et al*., 2017), HIV/AIDS (Andersson *et al*., 2019; Feyissa *et al*., 2019; Ma *et al*., 2019; Pantelic *et al*., 2019) and mental illness (Clement *et al*., 2013; Mehta *et al*., 2015; Hanisch *et al*., 2016; Büchter and Messer, 2017; Heim *et al*., 2018; Mills *et al*., 2020). In terms of stigma, most reviews focused on general stigma in relation to the condition (Clement *et al*., 2013; Sermrittirong *et al*., 2014; Mehta *et al*., 2015; Hanisch *et al*., 2016; Sommerland *et al*., 2017; Heim *et al*., 2018; Feyissa *et al*., 2019; Ma *et al*., 2019; Pantelic *et al*., 2019), but self-stigma (Büchter and Messer, 2017; Mills *et al*., 2020), and enacted, anticipated or internalised stigma (Andersson *et al*., 2019) were also considered. Some reviews focused on stigma in specific target groups or settings; namely affected persons and their families (Büchter and Messer, 2017; Andersson *et al*., 2019; Ma *et al*., 2019), healthcare workers (Heim *et al*., 2018; Feyissa *et al*., 2019) or low- and middle-income countries (Mehta *et al*., 2015; Heim *et al*., 2018; Pantelic *et al*., 2019). No evidence (i.e., systematic reviews or primary intervention studies) was identified for stigma-reduction directly in relation to COVID-19, SARS/MERS, influenza or Ebola. Most reviews included evidence from studies using a broad range of quantitative designs (generally this included randomised-control trials or RCTs) (Clement *et al*., 2013; Mehta *et al*., 2015; Hanisch *et al*., 2016; Feyissa *et al*., 2019; Ma *et al*., 2019; Pantelic *et al*., 2019), one focused on RCTs specifically (Büchter and Messer, 2017), and some included evidence from quantitative, qualitative and/or mixed methods studies (Sermrittirong *et al*., 2014; Sommerland *et al*., 2017; Heim *et al*., 2018; Andersson *et al*., 2019; Mills *et al*., 2020).

**Table 1.** Characteristics of articles providing evidence for this review

Study	Stigmatised condition	Intervention focus (stigma target)	Population	Results
Andersson *et al*. (2019)	HIV/AIDS	HIV-related enacted, anticipated or internalised stigma.	People with HIV	*n* = 27 studies. Group-based behavioural interventions, patient-centred mental health programmes and community support initiatives reduced stigma.
Büchter and Messer (2017)	Mental illness	Self-stigma	People with mental illness	*n* = 5 studies. No statistically significant effect found. None of included study found sustainable effects on recovery, help-seeking or self-stigma.
Clement *et al*. (2013)	Mental illness	Mental health-related stigma, mass media interventions	General public	*n* = 22 studies. Mass media interventions may reduce prejudice in the immediate, short and medium term, with small-to-medium effect.
Feyissa *et al*. (2019)	HIV/AIDS	HIV-related stigma in healthcare settings	Healthcare workers	*n* = 14 records reporting on eight studies. Training popular opinion leaders can reduce avoidance intent and prejudicial attitudes, and improve compliance to universal precaution (moderate quality evidence). Professionally assisted peer group interventions, modular interactive training, participatory self-guided assessment and intervention, contact strategy combined with information giving and empowerment can reduce stigma (very low quality evidence).
Hanisch *et al*. (2016)	Mental illness	Mental illness-related stigma at the workplace	People in the workplace	*n* = 16 studies. Ten interventions increased mental-health knowledge. Six studies (with high risk of bias) improved mental-health literacy. Nine studies improved attitudes. All types of anti-stigma interventions in 11 studies consistently had a significant positive impact on employees' supportive behaviour (except one study with only a marginally significant effect).
Heim *et al*. (2018)	Mental illness	Mental health-related stigma in primary health care settings in low- and middle-income countries (LMICs)	Primary health care staff	*n* = 18 studies. Most studies found positive effects on attitudes of primary healthcare staff towards people with mental illness, but some effects were rather small.
Ma *et al*. (2019)	HIV/AIDS	HIV-related self-stigma	People living with HIV/AIDS and their families	*n* = 23 studies. Psycho-education, support for treatment adherence, psychotherapy, narrative interventions, and community participation interventions show promising trend of stigma reduction.
Mehta *et al*. (2015)	Mental illness	Question (1) Mental health-related stigma, with long- and medium-term follow-up Question (2) Mental health-related stigma in LMICs	Any	Question 1 *n* = 72 studies; Question 2 *n* = 11 studies. Question (1) Modest evidence for the effectiveness of anti-stigma interventions beyond 4 weeks follow-up (increasing knowledge, reducing stigmatising attitudes). Question (2) No statistically significant improvements in knowledge. Significant reductions in stigmatising attitudes. No studies assessed behavioural outcomes/discrimination.
Mills *et al*. (2020)	Mental illness	Self-stigma, self-help interventions	People with mental illness	*n* = 8 studies. Self-help interventions can reduce self-stigma, specifically depression personal stigma and help-seeking related self-stigma.
Pantelic *et al*. (2019)	HIV/AIDS	HIV-related self-stigma, in LMICs	People living with HIV and key populations affected by HIV	*n* = 20 studies. Structural interventions such as scale-up of antiretroviral treatment, prevention of medication stockouts, social empowerment and economic strengthening may help reduce self-stigma.
Sermrittirong *et al*. (2014)	Leprosy	Leprosy-related stigma	General public, medical staff, patients with leprosy	*n* = 25 studies. Interventions with evidence of effective stigma reduction: integration of leprosy programmes into general health care, Information Education and Communication programmes, socio-economic rehabilitation.
Sommerland *et al*. (2017)	Tuberculosis	Tuberculosis-related stigma	General public, people with tuberculosis, caregivers (including healthcare workers)	*n* = 7 studies. Knowledge-shaping and attitude-changing interventions reduced anticipated stigma. Home visits and support groups reduced both anticipated and internalised stigma.

The methodological quality of the included systematic reviews was mixed (for results see online Supplementary Materials). The most common limitations were a lack of registered review protocols, providing no rationale for why the review focused on given study designs, no references provided for excluded studies, no reporting of funding information for included studies, and no assessment of potential publication bias.

Additional evidence on stigma reduction was included in relation to COVID-19 (American Psychological Association, 2020; Asmundson and Taylor, 2020; Centres for Disease Control and Prevention, 2020; Devakumar *et al*., 2020; Earnshaw, 2020; IASC Inter-Agency Standing Committee, 2020; Lin 2020; Logie and Turan, 2020; Nature, 2020; World Health Organization, 2020a, 2020b), SARS (Person *et al*., 2004), influenza (Barrett and Brown, 2008; Earnshaw and Quinn, 2013), Ebola (Davtyan *et al*., 2014; IASC Reference Group on Mental Health and Psychosocial Support, 2015; Mayrhuber *et al*., 2017), tuberculosis (Chang and Cataldo, 2014), leprosy (Topp *et al*., 2019), HIV/AIDS (Mak *et al*., 2017; Hartog *et al*., 2020), mental illness (Thornicroft *et al*., 2016; Janoušková *et al*., 2017; Nyblade *et al*., 2019) and mixed conditions (Mak *et al*., 2006, 2009; Fischer *et al*., 2019; Rao *et al*., 2019; World Health Organization, 2019). These additional materials represented a mixture of editorials, commentaries, opinion pieces, correspondence and narrative reports; technical guidance, or briefing papers/reports; data-based studies reporting on stigma experiences; systematic reviews (that were not selected as key evidence for this review, or that did not meet criteria for inclusion as evidence); and one scoping review. For further details see online Supplementary Materials.

Recommendations for principles and actions to reduce stigma and discrimination related to COVID-19 were made based on these data providing evidence and insights regarding effective stigma-reduction in relation to highly infectious and stigmatised conditions, and stigma during infectious disease outbreaks.

The recommendations are prefaced with the following *overarching considerations*:

- The link between stigma and COVID-19, and its impact, should be considered for people who have a current confirmed COVID-19 diagnosis, and also people who have been in COVID-19 quarantine, received COVID-19 treatment, and people associated with COVID-19 due to their work (e.g. health or social care workers), ethnicity or country of origin (given public perceptions of populations among whom the virus is considered more common), or an affiliation with a person who is unwell or otherwise associated with the virus (e.g. caregivers, family members, peers).
- Interventions to reduce COVID-19 stigma are recommended in all countries (high-, middle- and low-income) as stigma is likely to manifest across cultures where effective interventions to reduce such stigma are likely to share similar basic characteristics (subject to local modifications/adaptations).
- All intervention messages should be accessible to people of different ethnic and cultural groups, people with limited literacy, and people who require accessible formats due to disability. Inviting these groups to participate in planning and implementation of activities related to reducing COVID-19 stigma improves the quality and effectiveness of such messaging.
- Politicisation of management of the pandemic must be avoided as this may lead to further stigma and discrimination, and reduce the effectiveness of other efforts to combat COVID-19. Impacts of negatively singling out populations affected by the pandemic is likely to endure after the current health crisis. The recommendations are most effective if carried out together, as part of a comprehensive response implemented within an integrated approach to promote social inclusion and individual, family and community recovery.

*Specific recommendations* on stigma reduction were developed in relation to: (1) language and words used in relation to COVID-19; (2) media and journalistic practices; (3) public health interventions; (4) targeted interventions for key groups and (5) involving communities and key stakeholders. These recommendations are outlined in Box 1, and elaborated in the online Supplementary Materials.

Box 1. Recommendations for reducing stigma and discrimination related to COVID-19

1. Language and words†

1.1 Language and words reflecting stigmatising attitudes should not be used when talking about COVID-19. It is recommended: not to attach locations or ethnicity to the disease, not to refer to people with COVID-19 as ‘cases’, ‘victims’ or ‘suspects’; not to use exaggerated language or metaphors (e.g. ‘plague’).
1.2 Over-emphasising attribution of disease burden, severity and death to ethnicity, pre-illness behaviour/travel history, age, gender or underlying medical conditions should be avoided as it devalues affected people, assigns blame, can lead to a false sense of security in the rest of the population, and undermine epidemic control measures.

1. Media and Journalists†

2.1 Language in the media should be considered both as a delivery platform for anti-stigma strategies and as a target for anti-stigma efforts, as media reporting can shape popular perceptions, discourse, communication and behaviour.
2.2 Mass media should be employed to share balanced and accurate information, focused on avoiding COVID-19 stigma. Sensationalist headlines/stories should be avoided. Journalists have an ethical responsibility to convey messages based on good science and disease management principles.
2.3 Communication training should be provided for those in government and in health/care services, including those providing public briefings.
2.4 People affected by COVID-19 should be involved in shaping media language.

1. Public health interventions

3.1 COVID-19 stigma-reduction strategies targeting the general public should build on knowledge-shaping and attitude-changing strategies.*
3.2 Stigma among the general public can be reduced by providing treatment programmes for stigmatised conditions within general health care settings; this consideration is particularly relevant in the treatment of possible long-term complications following COVID-19*
3.3 Mass media interventions targeting the general public are recommended to reduce prejudice in the immediate, short and medium term.*
3.4 Anti-stigma campaigns should include correcting myths, rumours and stereotypes, and challenging bias. Emphasis should go beyond factual knowledge, to strategically addressing specific public misconceptions, which may vary among different populations and cultural/religious groups.†
3.5 Using artists and art to showcase stories, conditions and experiences of people who have suffered discrimination can cultivate engagement, empathy, acceptance and social change.†
3.6 Messaging should emphasise the joint social responsibility to support efforts to reduce impacts of COVID-19. Focus on a sense of community and jointly achieved positive outcomes is more likely to succeed than actions directed by fear or shaming. Messaging should include publicly supporting frontline workers, those who volunteer/assist vulnerable populations, and people from these communities (often disproportionately represented among frontline workers).†
3.7 Strategies to reduce and/or slow down transmission through avoiding physical contact should be framed as ‘physical distancing’, rather than ‘social distancing’ or ‘social isolation’. Remaining socially connected, and promoting a sense of community, support, common purpose and inclusion is likely to improve solidarity.†
3.8 Universal public health strategies (e.g. testing; stimulus checks; policies like physical distancing, travel bans and quarantine) can reduce stigma (vs. targeted strategies, which can imply blame on particular individuals/groups).†
3.9 Excessive policing or criminalising the breaching of COVID-19-related health policies is likely to increase stigma and discrimination, and risks loss of trust which may in turn reduce compliance with such measures or lead to protests.†
3.10 Comprehensive stigma mitigation requires public health strategies implemented in the immediate term (e.g. addressing misinformation) complemented by efforts to tackle societal-level issues of social and economic inequalities that facilitate stigma in the long term (e.g. racism, xenophobia, structural-level policies and laws).†

1. Targeted public health interventions for key groups

*People directly affected by COVID-19*

4.1 Stigma (anticipated, enacted, internalised) can be reduced through strategies building on group-based interventions, psycho-educational interventions, social empowerment strategies, community-based strategies and self-help interventions.*

*Family members*

4.2 Interventions should build on positive, community-proposed coping strategies. Family-based interventions and strategies based on empowerment are recommended to mitigate self-stigma.†

*Health care and frontline workers*

4.3 Health care professionals and key frontline workers need protection from discrimination and abuse, as this will increase stress and work-pressures, affecting their ability to work. Interventions to support and provide encouragement/counselling for those on COVID-19 frontlines are strongly recommended.*
4.4 An information-based approach, including the involvement of popular opinion leaders, should be implemented to reduce stigma against health workers.*

*Vulnerable/high-risk populations*

4.5 Tailored anti-stigma interventions and protection should be ensured for disadvantaged and marginalised groups (e.g. homeless people, people with disabilities, people who are incarcerated, migrants and refugees, and racial minorities), who may be particularly exposed to stigma and exclusion and at increased risk of COVID-19.†

1. Involving communities and key stakeholders†

5.1 Involving persons affected by COVID-19 is a key factor for successful anti-stigma interventions; particularly using voices, stories, recovery and hope narratives and images of local people.
5.2 Messaging should be contextualised and targeted, based on local knowledge of the specific beliefs and fears and drive stigma in a given setting, community or population.
5.3 Context-specific stigma can be understood through scoping information from local organisations, community leaders, clinicians, news messages, public health websites and social media posts.
5.4 Partnering with community leaders is essential to build trust, and develop and implement contextually appropriate anti-stigma strategies. They can draw on interpersonal connections to promote reassurance, add legitimacy to general public health efforts, and disseminate information to those who might mistrust official communication channels.

Key: *Recommendation based on evidence from **systematic reviews** selected for inclusion; †Recommendation based on **additional materials** (i.e. commentaries, reviews and other papers relevant for this review).

## Discussion

This rapid review developed timely and feasible recommendations for reducing stigma and discrimination in relation to COVID-19, thus responding to calls for scientific advice and evidence-based recommendations for stigma-reduction strategies in this public health emergency (Nature, 2020). Our recommendations specify overarching considerations that should underpin any efforts to reduce COVID-19-related stigma, from the outset of the pandemic and beyond, as well as providing strategies and actions to take at the micro-, meso- and macro levels to reduce stigma and discrimination.

Our recommendations represent the first consolidated set of guidance on principles for reducing stigma and discrimination in relation to COVID-19, responding to recent calls for scientific advice and evidence-based recommendations for stigma-reduction strategies in relation to COVID-19. Given the negative impact stigma and discrimination on people's physical and mental health and wellbeing, social equity, livelihoods and on efforts to control the disease outbreak (Stangl *et al*., 2019; Devakumar *et al*., 2020; World Health Organization, 2020b), these recommended stigma-reduction strategies are of critical importance within the wider context of responses to the pandemic.

These recommendations are anticipated to be actionable by policy makers, public health officials, planners and managers at the local and national levels, researchers, media representatives, national and international non-governmental organisations, community-based organisations, people affected by COVID-19 (directly or by association), and lay people.

The strength of this review and the resulting recommendations for principles of stigma-reduction related to COVID-19 lies in the transparent and robust methodologies that were applied for sourcing evidence and other materials for inclusion and data synthesis, the multidisciplinary sources of evidence, and the development of recommendations following a transparent and rigorous WHO methodology.

These recommendations do, however, need to be considered in view of some limitations. Given the urgency, a rapid review approach was taken for the systematic searches, and an expedited procedure utilised for the process to develop the recommendations, as per WHO guidance. Although rapid reviews methodologies in response to developing emergencies have limitations (Clarke, 2020), it is a recommended approach for feasible and timely evidence gathering and synthesis, and for the development of expedited recommendations for action. Rapid reviews are intended to provide initial guidance in emergencies, but we believe our work will also make a contribution beyond the immediate ongoing emergency given the long-term perspective of the recommendations, and the robust methodology underpinning the review. We envision that these recommendations provide a starting point for further, more comprehensive stigma reduction recommendations in relation to COVID-19. For now, implementing a full WHO guideline development procedure would have been excessively time consuming (these processes typically take 1–2 years), which is unfeasible under circumstances where stigma-reduction in relation to COVID-19 is highlighted as a priority. It also needs to be noted that these recommendations are based on a mixture of evidence from systematic reviews and insights from additional materials. Although the use of evidence plus additional considerations is part of the GRADE approach, we acknowledge that our recommendations are based on a limited evidence base. Thus, to make the most of all available information, an a-priori decision was made to consider evidence from systematic reviews and primary intervention studies, as well as insights from the additional materials to inform recommendations. The additional materials could not be assessed for methodological quality, given how no rating tool is available that would allow for useful comparisons between the range of materials that were included. To allow for recommendations to be assessed in view of this, we have indicated whether each recommendation is based on systematic review evidence or additional materials. Given the recency of the COVID-19 pandemic, no direct evidence on effective stigma reduction in relation to COVID-19 was available to inform the recommendations. We had accounted for this possibility by planning to extrapolate evidence on effective stigma-reduction also from other disease outbreaks and infectious/stigmatised conditions. This means the recommendations are based on the assumption of generalisability between the conditions. However, this approach is in line with recommendations to consider health-related stigma across conditions, rather than separately for each condition in a siloed manner, to facilitate public health responses and the ability to act on stigma (Stangl *et al*., 2019; Van Brakel *et al*., 2019). Using learnings on stigma-reduction strategies from previous disease outbreaks and infectious/stigmatised conditions to inform strategies in relation to COVID-19 is, in our view, the best available approach to formulate informed recommendations where direct evidence is not available, and is an approach that has been utilised previously (Mak *et al*., 2009; Davtyan *et al*., 2014; Fischer *et al*., 2019).

Future research should continue to assess the nature of stigma in relation to COVID-19, and the implementation and impact of the recommended stigma-reduction strategies, including, for example, qualitative operational research in humanitarian settings, the scalability and sustainability of anti-stigma interventions, and the cost-effectiveness of such interventions. The evidence should be used to revise and refine the recommendations regarding best practice to reduce stigma in relation to COVID-19 and other infectious disease epidemics. Studies are also needed to examine commonalities and differences in stigma and discrimination in differences in cultures and settings. Comparing the effectiveness of interventions in different populations will help confirm the extent to which interventions are generalisable across settings. People who have direct experience of COVID-19 associated stigma should play a key role in the development and implementation of such research. Research efforts should attempt to avoid the common limitations of stigma research, such as small-scale studies, heterogeneous designs, mixed methodological quality, lack of contextually and culturally adapted interventions, unvalidated outcome measures, limited evaluation of long-term impacts of interventions and behavioural outcomes, and limited evidence from low- and middle-income settings. Methodologically sound research generates stronger evidence, and increased harmonisation across studies would allow for the results to be pooled using robust synthesis methods for increased impact.

In conclusion, the recommendations developed through this rapid review at the onset of the pandemic provide strategies for the reduction of stigma and discrimination associated with COVID-19 at the micro-, meso- and macro level. They are a necessary complement to wider public health measures, both at the onset of a pandemic and beyond, and we urge administrations and decision-makers to recognise these recommendations and how these practices can be integrated into the ongoing COVID-19 public health, social and humanitarian sectors response, and subsequent long-term plans, to counter the stigma and discrimination evident in relation to the pandemic at all stages of an outbreak, including societies' recovery from it. Mitigating the impact of stigma is of critical importance, not only to reduce distress and negative experiences for people who are discriminated against, but also to strengthen communities' resolve to work together during exceptional circumstances, and facilitate adherence to public health strategies and health behaviours that help to control a viral disease outbreak. Stigma-reduction strategies will not only reduce distress and negative experiences in the immediate term, but critically a commitment to the reduction of stigma and discrimination will ultimately address deeper patterns of structural inequalities that drive stigma, social and economic marginalisation, and exacerbate both health risks and the impacts of stigma, in relation to COVID-19 and health conditions overall. Social inclusion, justice and solidarity are key components of health protection, required in the immediate term to manage the current COVID-19 public health emergency, but also in the long term for communities and countries to recover from its impact, and to be better prepared to respond to further waves of the outbreak or to comparable pandemics in the future.

## Supplementary material

For supplementary material accompanying this paper visit https://doi.org/10.1017/S2045796021000056. click here to view supplementary material

## Availability of data and materials

Additional data available from the corresponding author at petra.gronholm@kcl.ac.uk.
