# Supplementary material for: Reducing stigma and discrimination associated with COVID-19: early stage pandemic rapid review and practical recommendations
Source: Epidemiol Psychiatr Sci. 2021 Jan 28;30:e15. doi: 10.1017/S2045796021000056 (PMC7884669; doi:10.1017/S2045796021000056)
Supplement: Supplementary file 1 [file S2045796021000056sup001.docx]

**Online Supplemental Materials**

**Reducing stigma and discrimination associated with COVID-19: early stage pandemic rapid review and practical recommendations**

Petra C Gronholm^¶^, Michela Nosé^¶^, Wim H van Brakel, Julian Eaton, Bassey Ebenso, Kathy Fiekert, Fahmy Hanna, Maria Milenova, Charlene Sunkel, Corrado Barbui^&^, & Graham Thornicroft^&^

^¶^joint first authors; these authors contributed equally to this work

^&^joint last authors; these authors contributed equally to this work

[EVIDENCE PROFILE 2](#_Toc61644743)

[PICO QUESTION 2](#_Toc61644744)

[Background on the PICO question 2](#_Toc61644745)

[PART 1: EVIDENCE REVIEW 4](#_Toc61644746)

[Population/ Intervention / Comparison / Outcome (PICO) 4](#_Toc61644747)

[Search Strategy 5](#_Toc61644748)

[Evidence articles: abstracts 9](#_Toc61644749)

[Additional materials: narrative description or abstract 19](#_Toc61644750)

[Quality assessment: AMSTAR-2 40](#_Toc61644751)

[PART 2: FROM EVIDENCE TO RECOMMENDATIONS 41](#_Toc61644752)

[Evidence to Decision Table 41](#_Toc61644753)

[GDG Recommendations 45](#_Toc61644754)

[Appendix 1: Search strategy document 50](#_Toc61644755)

# EVIDENCE PROFILE

## PICO QUESTION

Which interventions are effective in reducing COVID-19 related stigma?

## Background on the PICO question

Novel coronavirus pneumonia was first detected in Wuhan, China, in late December 2019. This disease, caused by the virus SARS-CoV-2 (severe acute respiratory syndrome coronavirus 2), has been officially named coronavirus disease 2019, or COVID-19 by the World Health Organization (WHO). WHO declared the spread of the disease a "Public Health Emergency of International Concern" on 30 January 2020, and a pandemic on 11 March 2020 (1).

Public health emergencies, such as the outbreak of coronavirus disease 2019 (COVID-19), are stressful times for individuals, families, communities and nations. Given that COVID-19 is a new disease, it is understandable that its emergence and spread has caused confusion, anxiety and fear among the general public. Fear and anxiety about COVID-19 can, however, as with other perceived health threats, also result in stigma and discrimination toward people, places, or things associated with the disease (2).

The 2020 COVID-19 outbreak has provoked stigmatisation and discriminatory behaviours against people of certain ethnic backgrounds as well as anyone perceived to have been in contact with the virus (3,4). Anecdotal evidence and media reporting (5–7) outline experiences of stigma and discrimination amongst people affected by or associated with COVID-19. These reports are in line with previous evidence indicating that stigma has been a key concern for many in relation to comparable viral outbreaks and epidemics, e.g. severe acute respiratory syndrome (SARS), middle east respiratory syndrome (MERS), and Ebola virus disease (8–10).

Health-related stigma has been defined as a personal experience or social process characterised by exclusion, rejection, blame and devaluation, as a result of experiencing or anticipating negative social judgements due to a person or group being association with a given health condition (11).

In the process of stigmatisation, people are being labelled, stereotyped, separated, and/or experience loss of status and discrimination because of a potential negative affiliation with a disease (12). Given the complex ways in which stigma can occur and manifest, to facilitate classification, stigma has been defined as reflective of issues related to inaccurate knowledge (for example regarding an illness, how it spreads, and what people affected by the virus are like), attitudes (such as stereotyped negative beliefs, and associated negative emotional reactions), and behaviours (such as discriminatory behaviours, or shunning a person) (13,14).

Addressing stigma is important, as in addition to the negative behaviour towards people affected and associated, stigma can drive people to deny or hide the illness to avoid discrimination, prevent people from seeking health care immediately, and discourage people from adopting healthy behaviours (15,16). Such barriers could potentially contribute to more severe health problems, ongoing transmission, and difficulties controlling infectious diseases during a viral disease outbreak (17). Stigma and discrimination often affect the mental health of those who stigmatised, leading to anxiety and depression, which may worsen the above negative outcomes. Stigmatisation can also lead to the social rejection, avoidance and distancing of those who are feared (16). This can potentially lead to further harm, such as socially isolated people not being able to secure food or other basic necessities (18,19). The impact of stigma can be widespread. In relation to COVID-19, concerns regarding stigma are relevant not only for people who have a current confirmed or suspected infectious condition. Rather stigma can also occur after a person has been released from COVID-19 quarantine or is no longer symptomatic, even though they are no longer a risk for spreading the virus to others. Furthermore, stigma can also occur by association, meaning stigma may be experienced by people associated with COVID-19 due to their work (e.g. healthcare workers), country of origin or ethnicity (public perception of places and populations amongst whom the virus is more common), or affiliation with a person who is unwell (e.g. caregivers, family members).

The work of social scientists and anthropologists, crisis communicators and public health officials, to understand and counter COVID-19 stigmatization and is urgently needed (20–22). Such skill sets have been under-valued in past epidemics, resulting in delay in response and even attacks on health care staff, due to misinformation and related stigma (23).

In this context, the aim of this rapid review is to produce clinically and socially relevant and feasible recommendations regarding interventions to reduce stigma and discrimination related to COVID-19.

### References

1. WHO. WHO Director-General’s opening remarks at the media briefing on COVID-19 - 11 March 2020 [Internet]. 2020 [cited 2020 Apr 3]. Available from: https://www.who.int/dg/speeches/detail/who-director-general-s-opening-remarks-at-the-media-briefing-on-covid-19---11-march-2020

2. Centres for Disease Control and Prevention. Coronavirus Disease 2019 (COVID-19) Reducing stigma [Internet]. 2020 [cited 2020 Mar 27]. Available from: www.cdc.gov/coronavirus/2019-ncov/symptoms-testing/reducing-stigma.html

3. UNICEF. Social Stigma associated with COVID-19 [Internet]. 2020 [cited 2020 Mar 27]. Available from: https://www.unicef.org/media/65931/file/Social stigma associated with the coronavirus disease 2019 (COVID-19).pdf

4. Devakumar D, Shannon G, Bhopal SS, Abubakar I. Racism and discrimination in COVID-19 responses. Lancet [Internet]. 2020; Available from: http://dx.doi.org/10.1016/S0140-6736(20)30792-3

5. Phan S. The coronavirus panic is turning the UK into a hostile environment for east Asians. The Guardian [Internet]. 2020 Jan 27 [cited 2020 Apr 3]; Available from: https://www.theguardian.com/commentisfree/2020/jan/27/coronavirus-panic-uk-hostile-environment-east-asians

6. Buruma I. Virus as Metaphor. The New York Times [Internet]. 2020 Mar 28 [cited 2020 Apr 3]; Available from: https://www.nytimes.com/2020/03/28/opinion/coronavirus-racism-covid.html

7. Nylander D. Davina Nylander: How might the social stigma around covid-19 perpetuate the spread of disease? theBMJ Opinion [Internet]. 2020 Mar [cited 2020 Apr 3]; Available from: https://blogs.bmj.com/bmj/2020/03/23/social-stigma-surrounding-covid-19-perpetuate-spread-of-disease/

8. Person B, Sy F, Holton K, Govert B, Liang A, NCID/SARS Community Outreach Team. Fear and Stigma: The Epidemic within the SARS Outbreak. Emerg Infect Dis. 2004;10(2):358–63.

9. James PB, Wardle J, Steel A, Adams J. An assessment of Ebola-related stigma and its association with informal healthcare utilisation among Ebola survivors in Sierra Leone: A cross-sectional study. BMC Public Health. 2020;20(1):1–12.

10. Fukuda K, Wang R, Vallat B. Naming diseases: First do no harm. Science (80- ). 2015;348(6235):643.

11. Weiss MG, Ramakrishna J. Stigma interventions and research for international health. Lancet. 2006;367(9509):536–8.

12. Link BG, Phelan JC. Conceptualizing Stigma. Annu Rev Sociol [Internet]. 2001 Aug [cited 2013 Apr 2];27(1):363–85. Available from: http://www.jstor.org/stable/10.2307/2678626

13. Thornicroft G. Shunned: Discrimination against people with mental illness. Oxford, UK: Oxford University Press; 2006. 322 p.

14. Thornicroft G, Rose D, Kassam A, Sartorius N. Stigma: ignorance, prejudice or discrimination? Br J Psychiatry [Internet]. 2007 Mar [cited 2012 Nov 15];190(3):192–3. Available from: http://bjp.rcpsych.org/content/190/3/192

15. Knaak S, Mantler E, Szeto A. Mental illness-related stigma in healthcare: Barriers to access and care and evidence-based solutions. Healthc Manag Forum. 2017;30(2):111–6.

16. Stangl AL, Earnshaw VA, Logie CH, Van Brakel W, Simbayi LC, Barré I, et al. The Health Stigma and Discrimination Framework: A global, crosscutting framework to inform research, intervention development, and policy on health-related stigmas. BMC Med. 2019;17(1):18–23.

17. Van Bortel T, Basnayake A, Wurie F, Jambai M, Koroma AS, Muana AT, et al. Psychosocial effects of an Ebola outbreak at individual, community and international levels. Bull World Health Organ. 2016;94(3):210–4.

18. Togoh I. Spain: Elderly Care Home Residents Found Dead In Their Beds As Coronavirus Toll Worsens. Forbes [Internet]. 2020 Apr [cited 2020 Apr 14]; Available from: https://www.forbes.com/sites/isabeltogoh/2020/03/24/spain-elderly-care-home-residents-found- dead-in-their-beds-as-coronavirus-toll-worsens/#778718166707

19. BBC News. Coronavirus: Disabled boy dies in China after father quarantined [Internet]. BBC News. 2020 [cited 2020 Apr 14]. Available from: https://www.bbc.co.uk/news/world-asia-china-51362772

20. IASC Inter-Agency Standing Committee. Interim briefing note: Addressing mental health and psychosocial aspects of COVID-19 Outbreak [Internet]. 2020. Available from: https://interagencystandingcommittee.org/iasc-reference-group-mental-health-and-psychosocial-support-emergency-settings/interim-briefing

21. WHO. Mental Health and Psychosocial Considerations During COVID-19 Outbreak [Internet]. 2020. Available from: https://www.who.int/docs/default-source/coronaviruse/mental-health-considerations.pdf?sfvrsn=6d3578af_8

22. Nature. End coronavirus stigma now. 2020 Apr; Available from: https://media.nature.com/original/magazine-assets/d41586-020-01009-0/d41586-020-01009-0.pdf

23. Hall RCW, Hall RCW, Chapman MJ. The 1995 Kikwit Ebola outbreak: lessons hospitals and physicians can apply to future viral epidemics. Gen Hosp Psychiatry. 2008;30(5):446–52.

# PART 1: EVIDENCE REVIEW

## Population/ Intervention / Comparison / Outcome (PICO)

### Population:

People experiencing stigma due to being *affected by, or associated with*, COVID-19 or comparable stigmatised condition, directly or indirectly. This could be e.g.:

- People who are confirmed to have the condition
- People who are suspected to have the condition; whether symptomatic or asymptomatic
- People who have recovered from the condition
- People associated with the condition due to their work (e.g. healthcare or social care workers), country of origin or ethnicity (public perception of places and populations amongst whom the condition is more common), or affiliation with a person who is unwell (e.g. caregivers, family members)

People who can *act on stigma in relation* to COVID-19 or comparable stigmatised condition. This could be e.g.:

- People associated with the condition due to their work (e.g. healthcare workers), country of origin or ethnicity (public perception of places and populations amongst whom the condition is more common), or affiliation with a person who is unwell (e.g. caregivers, family members)
- Members of the general public
- Policy advisers (including civil servants, scientific/technical advisers) and policy makers
- People who have experience of the illness and their family members

All age groups are considered.

### Intervention:

Any type of interventions with a stated aim of reducing COVID-19 related stigma or with an implied aim of reducing stigma as indicated by the inclusion of at least one of the core stigma-related outcomes.

As we do not expect any studies on COVID-19, we will extrapolate data from other conditions with COVID-19 specific characteristics, namely infectious diseases with airborne transmission and communicable diseases, and other highly stigmatised conditions (SARS, MERS, Influenza (bird flu/H5N1/avian flu/avian influenza, Swine flu/H1N1), Ebola, Tuberculosis, Leprosy, HIV/AIDS, mental illness).

COMMENT: These conditions were selected based on a scoping review to identify recent comparable disease outbreaks, epidemics and pandemics in relation to which infection- or disease-related stigma had been discussed, infectious diseases and other health conditions in relation to which health-related stigma was an issue, and/or for which anti-stigma intervention evidence was available.

### Comparison:

None; searches are not restricted to controlled studies.

### Outcomes:

Main outcome

Stigma (incidence and burden).

No restriction placed on type of stigma; we will include e.g. public stigma, self-stigma, structural stigma, stigma-by-association, perceived stigma, anticipated stigma.

Measures of effect: Pooled percentages for categorical variables, and means for continuous variables, with their corresponding 95% Confidence Intervals (CIs)

Secondary outcomes

Outcomes not explicitly termed as "stigma", but that are theoretically considered reflective of stigma by virtue of capturing the following stigma-related components:

1. knowledge (e.g. public health awareness regarding given condition, negative misconceptions, myths, rumours);
2. attitudes (e.g. prejudice, stereotypes); or
3. behaviours (e.g. discrimination, violence, stigma coping/stigma resilience)

Measures of effect: Pooled percentages for categorical variables, and means for continuous variables, with their corresponding 95% CIs.

## Search Strategy

See ‘Systematic review search methods’ appendix 1.

### List of systematic reviews identified by the search process meeting the PICO criteria

Alonso M, Guillén AI, Muñoz M 2019. Interventions to Reduce Internalized Stigma in individuals with Mental Illness: A Systematic Review. Span J Psychol. 2019 May 14;22: E27. (EXCLUDED: Focused on severe mental illness specifically)

Andersson et al, 2019 Stigma reduction interventions in people living with HIV to improve health related quality of life, The Lancet, 24 (INCLUDED)

Bahtiar, Astuti Yuni Nursasi, Utilization of Interactive Educational Media in Improving Self Efficacy of Lung Tuberculosis Patients: Systematic Literature Review, Enferm Clin, 29 Suppl 2, 101-105 Sep 2019 (EXCLUDED: Not focused on anti-stigma intervention)

Büchter, R. B., & Messer, M. (2017). Interventions for reducing self-stigma in people with mental illnesses: a systematic review of randomized controlled trials. *GMS German Medical Science*, *15*, 1–12. <https://doi.org/10.3205/000248> (INCLUDED)

Chang et al, 2014 A Systematic Review of Global Cultural Variations in Knowledge, Attitudes and Health Responses to Tuberculosis Stigma Review Int J Tuberc Lung Dis, 18 (2), 168-73, i-iv Feb 2014 (EXCLUDED: Focused on conditions beyond inclusion criteria, included in additional material)

Clement S, Lassman F, Barley E, Evans-Lacko S, Williams P, Yamaguchi S, Slade M, Rüsch N, Thornicroft G, Mass media interventions for reducing mental health-related stigma (Review). Cochrane Database Syst Rev. 2013 Jul 23;(7):CD009453. doi: 10.1002/14651858.CD009453.pub2. PMID: 23881731 (INCLUDED)

Feyissa, G. T., Lockwood, C., Woldie, M., & Munn, Z. (2019). Reducing HIV-related stigma and discrimination in healthcare settings: A systematic review of quantitative evidence. *PLoS ONE*, *14*(1), 1–23. <https://doi.org/10.1371/journal.pone.0211298> (INCLUDED)

Feyissa GT, Lockwood C, Munn Z. The effectiveness of home-based HIV counselling and testing on reducing stigma and risky sexual behavior among adults and adolescents: A systematic review and meta-analyses. JBI Database System Rev Implement Rep. 2015 Jul 17;13(6):318-72. (EXCLUDED: More recent/comprehensive/relevant review was available)

Gronholm, P. C., Nye, E., & Michelson, D. (2018). Stigma related to targeted school-based mental health interventions: A systematic review of qualitative evidence. *Journal of Affective Disorders*, *240*(March), 17–26. <https://doi.org/10.1016/j.jad.2018.07.023> (EXCLUDED: Not focused on anti-stigma intervention)

Hanisch, S. E., Twomey, C. D., Szeto, A. C. H., Birner, U. W., Nowak, D., & Sabariego, C. (2016). The effectiveness of interventions targeting the stigma of mental illness at the workplace: a systematic review. *BMC Psychiatry*, *16*(1), 1. <https://doi.org/10.1186/s12888-015-0706-4> (INCLUDED)

Hartog et al, 2020. Stigma reduction interventions for children and adolescents in low- and middle-income countries: systematic review of intervention strategies Social Science & Medicine 246 (2020) 112749.) (EXCLUDED: Focused on conditions beyond inclusion criteria; included in additional material)

Heim E, Kohrt BA, Koschorke M, Milenova M, Thornicroft G. 2018. Reducing mental health-related stigma in primary health care settings in low- and middle-income countries: a systematic review. Epidemiol Psychiatr Sci. 2018 Sep 4;29: e3. (INCLUDED)

Heim, E., Henderson, C., Kohrt, B. A., Koschorke, M., Milenova, M., & Thronicroft, G. (2019). Reducing mental health-related stigma among medical and nursing students in low- and middle-income countries: a systematic review. *Epidemiology and Psychiatric Sciences*, *Apr 1*, 1–9. (EXCLUDED: More recent/comprehensive/relevant review was available)

Hofstraat K, Wim H van Brakel Social Stigma Towards Neglected Tropical Diseases: A Systematic Review Int Health, 8 Suppl 1, i53-70  Mar 2016 (EXCLUDED: Focused on conditions beyond inclusion criteria)

Janoušková M, Tušková E, Weissová A, Trančík P, Pasz J, Evans-Lacko S, et al. (2017) Can video interventions be used to effectively destigmatize mental illness among young people? A systematic review. Eur Psychiatry. Mar 1;41: 1–9. (EXCLUDED: More recent/comprehensive/relevant review was available; included in additional material)

Kemp et al (2019). Implementation science and stigma reduction interventions in low- and middle-income countries: a systematic review. *BMC Medicine*, *17*(6), 1–18. <https://doi.org/10.1186/s12916-018-1237-x> (EXCLUDED: Not focused on anti-stigma intervention)

Loutfy M, Tharao W, Logie C, Aden MA, Chambers LA, Wu W, Abdelmaseh M, Calzavara L. Systematic review of stigma reducing interventions for African/Black diasporic women. J Int AIDS Soc. 2015 Apr 8;18(1):19835. (EXCLUDED: More recent/comprehensive/relevant review was available)

Ma et al, 2019. Self-stigma reductions interventions for people living with HIV/AIDS and their families: a systematic review. AIDS and Behavior, 23:707-741 (INCLUDED)

Mak WWS, Mo PKH, Ma GYK, Lam MYY. Meta-analysis and systematic review of studies on the effectiveness of HIV stigma reduction programs. Soc Sci Med. 2017 Sep; 188:30-40. doi: 10.1016/j.socscimed.2017.06.045. Epub 2017 Jul (EXCLUDED: More recent/comprehensive/relevant review was available; included in additional material)

Mehta et al, 2015 Evidence for effective interventions to reduce mental health-related stigma and discrimination in the medium and long term: systematic review. The British Journal of Psychiatry 207, 377–384. doi: 10.1192/bjp.bp.114.151944 (INCLUDED)

Mellor C. School-based interventions targeting stigma of mental illness: systematic review. Psychiatr Bull (2014). 2014 Aug;38(4):164-71. (EXCLUDED: More recent/comprehensive/relevant review was available)

Mills, H., Mulfinger, N., Raeder, S., Rüsch, N., Clements, H., & Scior, K. (2020). Self-help interventions to reduce self-stigma in people with mental health problems: A systematic literature review. *Psychiatry Research*, *284*(July 2019), 112702. <https://doi.org/10.1016/j.psychres.2019.112702> (INCLUDED)

Morgan, A. J., Reavley, N. J., Ross, A., Too, L. S., & Jorm, A. F. (2018). Interventions to reduce stigma towards people with severe mental illness: Systematic review and meta-analysis. *Journal of Psychiatric Research*, *103*, 120–133. <https://doi.org/10.1016/j.jpsychires.2018.05.017> (EXCLUDED: Focused on severe mental illness specifically)

Nguyen, T., Holton, S., Tran, T., & Fisher, J. (2019). Informal mental health interventions for people with severe mental illness in low and lower middle-income countries: A systematic review of effectiveness. *International Journal of Social Psychiatry*, *65*(3), 194–206. <https://doi.org/10.1177/0020764019831322> (EXCLUDED: Not focused on anti-stigma intervention)

Nyblade et al, 2019 Stigma in Health Facilities: Why It Matters and How We Can Change It BMC Med, 17 (1), 25 2019 Feb 15 (EXCLUDED: Focused on conditions beyond inclusion criteria; included in additional material)

Pantelic, M., Steinert, J. I., Park, J., Mellors, S., & Murau, F. (2019). “Management of a spoiled identity”: Systematic review of interventions to address self-stigma among people living with and affected by HIV. *BMJ Global Health*, *4*(2). <https://doi.org/10.1136/bmjgh-2018-001285> (INCLUDED)

Rao et al, 2019 A Systematic Review of Multi-Level Stigma Interventions: State of the Science and Future Directions, BMC Med, 17 (1), 41, Feb 15 (EXCLUDED: Focused on conditions beyond inclusion criteria; included in additional material)

Ross AM, Morgan AJ, Jorm AF, Reavley NJ. [A systematic review of the impact of media reports of severe mental illness on stigma and discrimination, and interventions that aim to mitigate any adverse impact.](https://pubmed.ncbi.nlm.nih.gov/30349962/?from_term=%28%28social+stigma%5BMeSH+Terms%5D%29+OR+%28stigma%29+OR+%28discrimination%5BMeSH+Terms%5D%29+OR+%28discrimination%29%29+AND+%28%28mental+illness%29+OR+%28mental+health%29+OR+%28psychiatric+illness%29+OR+%28%22mentally+disabled+persons%22%5BMeSH+Terms%5D%29+OR+%28%22mental+disorders%22%5BMeSH+Terms%5D%29+OR+%28%22mental+health%22%5BMeSH+Terms%5D%29+OR+%28%22schizophrenia%22%5BMeSH+Terms%5D%29%29&from_filter=pubt.systematicreviews&from_filter=ds1.y_10&from_sort=date&from_page=10&from_pos=5) Soc Psychiatry Psychiatr Epidemiol. 2019 Jan;54(1):11-31. (EXCLUDED: Focused on conditions beyond inclusion criteria)

Sengupta S, Banks B, Jonas D, Miles MS, Smith GC. HIV interventions to reduce HIV/AIDS stigma: a systematic review. AIDS Behav. 2011 Aug;15(6):1075-87. (EXCLUDED: More recent/comprehensive/relevant review was available)

Sermrittirong et al, 2014. How to reduce stigma in Leprosy. A systematic Literature Review. Lepr Rev, 85 839, 149-157 (INCLUDED)

Sommerland et al, 2017. Evidence-based Interventions to Reduce Tuberculosis Stigma: A Systematic Review, Int J Tuberc Lung Dis, 21 (11), 81-86 2017 Nov 1 (INCLUDED)

Stangl AL, Lloyd JK, Brady LM, Holland CE, Baral S. A systematic review of interventions to reduce HIV-related stigma and discrimination from 2002 to 2013: how far have we come? J Int AIDS Soc. 2013 Nov 13;16(3 Suppl 2):18734. (EXCLUDED: More recent/comprehensive/relevant review was available)

Thornicroft G, Mehta N, Clement S, Evans-Lacko S, Doherty M, Rose D, Koschorke M, Shidhaye R, O'Reilly C, Henderson C. Evidence for effective interventions to reduce mental-health-related stigma and discrimination. Lancet. 2016 Mar 12;387(10023):1123-1132 (EXCLUDED: Not a systematic review, included in additional material

Topp et al, 2019. Strategies to reduce stigma related to visible chronic skin disease: a systematic review, JEADV, 33, 2029-2038 (EXCLUDED: Focused on conditions beyond inclusion criteria; included in additional material)

Tsang, H. W. H., Ching, S. C., Tang, K. H., Lam, H. T., Law, P. Y. Y., & Wan, C. N. (2016). Therapeutic intervention for internalized stigma of severe mental illness: A systematic review and meta-analysis. *Schizophrenia Research*, *173*(1–2), 45–53. <https://doi.org/10.1016/j.schres.2016.02.013> (EXCLUDED: Focused on severe mental illness specifically)

Wood, L., Byrne, R., Varese, F., & Morrison, A. P. (2016). Psychosocial interventions for internalised stigma in people with a schizophrenia-spectrum diagnosis: A systematic narrative synthesis and meta-analysis. *Schizophrenia Research*, *176*(2–3), 291–303. <https://doi.org/10.1016/j.schres.2016.05.001> (EXCLUDED: Focused on severe mental illness specifically)

Xu Z, Rüsch N, Huang F, Kösters M. Challenging mental health related stigma in China: Systematic review and meta-analysis. I. Interventions among the general public. Psychiatry Res. 2017 Sep; 255:449-456. (EXCLUDED: More recent/comprehensive/relevant review was available)

Yamaguchi S, Wu SI, Biswas M, Yate M, Aoki Y, Barley EA, Thornicroft G. Effects of short-term interventions to reduce mental health-related stigma in university or college students: a systematic review. J Nerv Ment Dis. 2013 Jun;201(6):490-503 (EXCLUDED: More recent/comprehensive/relevant review was available not the last updated)

### List of commentaries, reviews and other papers, as identified by the search process and expert consultation that went into additional materials

American Psychological Association 2020. Combating bias and stigma related to COVID-19. How to stop the xenophobia that’s spreading along with the coronavirus. <https://www.apa.org/topics/covid-19-bias>

Asmundson G. (2020) Coronaphobia: Fear and the 2019-nCoV outbreak. Journal of Anxiety Disorders 70, 102196

Barrett et al, 2008. Stigma in the Time of Influenza: Social and Institutional Responses to Pandemic Emergencies. J Infect Dis , 197 Suppl 1, S34-S7

Centers for Disease Control and Prevention, 2020. Coronavirus Disease (COVID-19) Reducing stigma. [www.cdc.gov/coronavirus/2019-ncov/symptoms-testing/reducing-stigma.html](http://www.cdc.gov/coronavirus/2019-ncov/symptoms-testing/reducing-stigma.html)

Davtyan et al. Addressing Ebola-related Stigma: Lessons Learned from HIV/AIDS 1 HIV infection. Glob Health Action 2014, 7: 26058 - <http://dx.doi.org/10.3402/gha.v7.26058>

Devakumar et al, 2020. Racism and discrimination in COVID-19 responses. Lancet.com Published online April 1, 2020

Earnshaw V. Don’t Let Fear of COVID -19 Turn into Stigma. Harvard Business Review [Internet]. 2020 Apr; Available from: https://hbr.org/2020/04/dont-let-fear-of-covid-19-turn-into-stigma

Earnshaw et al, 2013. Influenza Stigma During the 2009 H1N1 Pandemic. Appl Soc Psychol, 43 (Suppl 1) 2013 Jun 1

Fischer et al, 2019. Addressing Disease-Related Stigma During Infectious Disease Outbreaks. Disaster Med Public Health Prep. 2019 Dec;13(5-6):989-994

IASC Reference Group on Mental Health and Psychosocial Support. Mental Health and Psychosocial Support in Ebola Virus Disease Outbreaks. A Guide for Public Health Programme Planners. Geneva; 2015.

IASC Inter-Agency Standing Committee. Interim briefing note: Addressing mental health and psychosocial aspects of COVID-19 Outbreak [Internet]. 2020. Available from: https://interagencystandingcommittee.org/iasc-reference-group-mental-health-and-psychosocial-support-emergency-settings/interim-briefing

Lin CY, 2020 Social reaction toward the 2019 novel coronavirus (COVID-19). Soc Health Behav; 3:1-Mak et al, 2006 Comparative stigma of HIV/AIDS, SARS, and tuberculosis in Hong Kong. Soc Sci Med. 2006

Logie, C. H., & Turan, J. M. (2020). How Do We Balance Tensions Between COVID-19 Public Health Responses and Stigma Mitigation? Learning from HIV Research. *AIDS and Behavior*, (0123456789), 1–4. <https://doi.org/10.1007/s10461-020-02856-8>

Mayrhuber, E. A. S., Niederkrotenthaler, T., & Kutalek, R. (2017). “We are survivors and not a virus:” Content analysis of media reporting on Ebola survivors in Liberia. *PLoS Neglected Tropical Diseases*, *11*(8), 1–19. https://doi.org/10.1371/journal.pntd.0005845

Mak WWS, Mo PKH, Cheung RYM, Woo J, Cheung FM, Lee D. (2006) Comparative stigma of HIV/AIDS, SARS, and Tuberculosis in Hong Kong. *Soc Sci Med*; **63**: 1912–22.

Mak WWS, Cheung F, Woo J, *et al.* (2009) A comparative study of the stigma associated with infectious diseases (SARS, AIDS, TB). *Hong Kong Med J*; **15**: 34–7.

Nature. (2020, April). *End coronavirus stigma now*. Retrieved from https://media.nature.com/original/magazine-assets/d41586-020-01009-0/d41586-020-01009-0.pdf

Person et al, 2004 Fear and Stigma: The Epidemic within the SARS outbreak, Emerging infectious Diseases, vol 10, 2 pag 358-363

World Health Organization (2020a), Social stigma associated with COVID-19, 27 March 2020 https://www.who.int/docs/default-source/coronaviruse/covid19-stigma-guide.pdf?sfvrsn=226180f4_2

World Health Organization (2020b), Mental health and psychosocial consideration during the COVID-19 outbreak, 18 March 2020 <https://www.who.int/docs/default-source/coronaviruse/mental-health-considerations.pdf>

World Health Organization, 2019. What is the evidence on the role of the arts in improving health and well-being? A scoping review. Health Evidence Network synthesis report 67, Fancourt & Finn, <http://www.euro.who.int/en/publications/abstracts/what-is-the-evidence-on-the-role-of-the-arts-in-improving-health-and-well-being-a-scoping-review-2019>

### List of systematic reviews identified by the search process that went into summary tables

Andersson et al, 2019 Stigma reduction interventions in people living with HIV to improve health related quality of life, Lancet HIV. 2020 Feb;7(2):e129-e140. doi: 10.1016/S2352-3018(19)30343-1.

Büchter, R. B., & Messer, M. (2017). Interventions for reducing self-stigma in people with mental illnesses: a systematic review of randomized controlled trials. *GMS German Medical Science*, *15*, 1–12. <https://doi.org/10.3205/000248>

Clement S, Lassman F, Barley E, Evans-Lacko S, Williams P, Yamaguchi S, Slade M, Rüsch N, Thornicroft G, Mass media interventions for reducing mental health-related stigma (Review). Cochrane Database Syst Rev. 2013 Jul 23;(7):CD009453. doi: 10.1002/14651858.CD009453.pub2. PMID: 23881731

Feyissa, G. T., Lockwood, C., Woldie, M., & Munn, Z. (2019). Reducing HIV-related stigma and discrimination in healthcare settings: A systematic review of quantitative evidence. *PLoS ONE*, *14*(1), 1–23. https://doi.org/10.1371/journal.pone.0211298

Hanisch, S. E., Twomey, C. D., Szeto, A. C. H., Birner, U. W., Nowak, D., & Sabariego, C. (2016). The effectiveness of interventions targeting the stigma of mental illness at the workplace: a systematic review. *BMC Psychiatry*, *16*(1), 1. <https://doi.org/10.1186/s12888-015-0706-4>

Heim E, Kohrt BA, Koschorke M, Milenova M, Thornicroft G. 2018. Reducing mental health-related stigma in primary health care settings in low- and middle-income countries: a systematic review. Epidemiol Psychiatr Sci. 2018 Sep 4;29: e3.

Ma et al, 2019. Self-stigma reductions interventions for people living with HIV/AIDS and their families: a systematic review. AIDS and Behavior, 23:707-741

Mehta et al, 2015 Evidence for effective interventions to reduce mental health-related stigma and discrimination in the medium and long term: systematic review. The British Journal of Psychiatry 207, 377–384. doi: 10.1192/bjp.bp.114.151944

Mills, H., Mulfinger, N., Raeder, S., Rüsch, N., Clements, H., & Scior, K. (2020). Self-help interventions to reduce self-stigma in people with mental health problems: A systematic literature review. *Psychiatry Research*, *284*(July 2019), 112702. https://doi.org/10.1016/j.psychres.2019.112702

Pantelic, M., Steinert, J. I., Park, J., Mellors, S., & Murau, F. (2019). “Management of a spoiled identity”: Systematic review of interventions to address self-stigma among people living with and affected by HIV. *BMJ Global Health*, *4*(2). <https://doi.org/10.1136/bmjgh-2018-001285>

Sermrittirong et al, 2014. How to reduce stigma in Leprosy. A systematic Literature Review. Lepr Rev, 85 839, 149-157

Sommerland et al, 2017. Evidence-based Interventions to Reduce Tuberculosis Stigma: A Systematic Review, Int J Tuberc Lung Dis, 21 (11), 81-86 2017 Nov 1

## Evidence articles: abstracts

i.e. Abstracts of key systematic reviews of interventions, and primary intervention studies that went into summary tables

These key systematic review articles were selected primarily based on recency (i.e. including the most recent review article, assumed to include the most up-to-date overview of available evidence, including the content captured in earlier reviews on the same topic). A consideration of comprehensiveness did, however, also guide selection - if the most recent systematic review had a very narrow focus (e.g. a particular age group or geographic region), and another semi-contemporaneous review on the topic was available that had used a broader search strategy identifying more articles, then the more comprehensive systematic review was selected for inclusion in an effort to capture a broader set of evidence. Decisions regarding inclusion were made through discussion between the two lead authors and endorsed by the full review group (all authors).

| Andersson et al, 2019 | The authors provide a narrative focused review of stigma reduction interventions that have implication for health-related quality of life for people living with HIV across all income setting.  As the UNAIDS 90-90-90 targets for people living with HIV are increasingly being reached in many contexts, health-related quality of life, the so-called fourth 90, warrants special attention. HIV-related stigma and discrimination are major barriers for overall health-related quality of life despite impressive clinical and virological improvements in HIV care. There is a scarcity of well-designed intervention studies that document stigma reduction in people living with HIV and few studies that specifically assess the effect of stigma on health-related quality of life. Further, few interventions target discrimination from providers outside of HIV-specific care or involve people living with HIV in both the design and implementation. Lastly, evidence on methods to reduce stigma in several under-represented key populations and geographical regions is insufficient and research on intersectional stigma (i.e., the convergence of multiple stigmatised identities) needs further attention. |
| --- | --- |
| Büchter et al.,2017 | Background: Self-stigma occurs when people with mental illnesses internalize negative stereotypes and prejudices about their condition. It can reduce help-seeking behaviour and treatment adherence. The effectiveness of interventions aimed at reducing self-stigma in people with mental illness is systematically reviewed. Results are discussed in the context of a logic model of the broader social context of mental illness stigma. Methods: Medline, Embase, PsycINFO, ERIC, and CENTRAL were searched for randomized controlled trials in November 2013. Studies were assessed with the Cochrane risk of bias tool. Results: Five trials were eligible for inclusion, four of which provided data for statistical analyses. Four studies had a high risk of bias. The quality of evidence was very low for each set of interventions and outcomes. The interventions studied included various group based anti-stigma interventions and an anti-stigma booklet. The intensity and fidelity of most interventions was high. Two studies were considered to be sufficiently homogeneous to be pooled for the outcome self-stigma. The meta-analysis did not find a statistically significant effect (SMD [95% CI] at 3 months: -0.26 [-0.64, 0.12], I2=0%, n=108). None of the individual studies found sustainable effects on other outcomes, including recovery, help-seeking behaviour and self-stigma. Conclusions: The effectiveness of interventions against self-stigma is uncertain. Previous studies lacked statistical power, used questionable outcome measures and had a high risk of bias. Future studies should be based on robust methods and consider practical implications regarding intervention development (relevance, implementability, and placement in routine services). |
| Clement et al., 2013 | People define stigma in various ways. In this review we focus on two key aspects of stigma: discrimination (treating people unfairly because of the group they belong to) and prejudice (negative attitudes and emotions towards certain groups). People with mental health problems often experience stigma. It can have awful effects on their lives. Mass media are media that are intended to communicate with large numbers of people without using face-to-face contact. Examples include newspapers, billboards, pamphlets, DVDs, television, radio, cinema, and the Internet. Anti-stigma campaigns often include mass media interventions, and can be expensive, so it is important to find out if the use of mass media interventions can reduce stigma. We reviewed studies comparing people who saw or heard a mass media intervention about mental health problems with people who had not seen or heard any intervention, or who had seen an intervention which contained nothing about mental ill health or stigma. We aimed to find out what effects mass media interventions may have on reducing stigma towards people with mental health problems. We found 22 studies involving 4490 people. Five of these studies had data about discrimination and 19 had data about prejudice. We found that mass media interventions may reduce, increase, or have no effect on discrimination. We found that mass media interventions may reduce prejudice. The amount of the reduction can be considered as small to medium, and is similar to reducing the level of prejudice from that associated with schizophrenia to that associated with major depression. The quality of the evidence about discrimination and prejudice was low, so we cannot be very certain about these findings. Only three studies gave any information about financial costs and two about adverse effects, and there were limitations in how they assessed these, so we cannot draw conclusions about these aspects. |
| Feyissa et al, 2019 | Introduction: Stigma and discrimination (SAD) related to HIV compromise access and adherence to treatment and support programs among people living with HIV (PLHIV). The ambitious goal of ending the epidemic of HIV by 2030 set by the United Nations Joint Program of HIV/AIDS (UNAIDS) will thus only be achieved if HIV-related stigma and discrimination are reduced. The objective of this review was to locate, appraise and describe international literature reporting on interventions that addressed HIV-related SAD in healthcare settings.  Methods: The databases searched were: Cumulative Index to Nursing and Allied Health (CINAHL), Excerpta Medica Database from Elsevier (EMBASE), PubMed and Psychological Information (PsycINFO) database. Two individuals independently appraised the quality of the papers using appraisal instruments from the Joanna Briggs Institute (JBI). Data were extracted from papers included in the review using the standardized data extraction tool from JBI. Quality of evidence for major outcomes was assessed using Grading of Recommendations, Assessment, Development and Evaluation (GRADE).  Results: The authors retained 14 records reporting on eight studies. Five categories of SAD reduction (information-based, skills building, structural, contact-based and biomedical interventions) were identified. Training popular opinion leaders (POLs) resulted in significantly lower mean avoidance intent scores (MD = -1.87 [95% CI -2.05 to -1.69]), mean prejudicial attitude scores (MD = -3.77 [95% CI -5.4 to -2.09]) and significantly higher scores in mean compliance to universal precaution (MD = 1.65 [95% CI 1.41 to 1.89]) when compared to usual care (moderate quality evidence). The Summary of Findings table (SOF) is shown in Tab 1.  Conclusions: Evidence of moderate quality indicates that training popular opinion leaders is effective in reducing avoidance intent and prejudicial attitude and improving compliance to universal precaution. Very low-quality evidence indicates that professionally-assisted peer group interventions, modular interactive training, participatory self-guided assessment and intervention, contact strategy combined with information giving and empowerment are effective in reducing HIV-related stigma. Further Randomized Controlled Trials (RCTs) are needed. Future trials need to use up-to-date and validated instruments to measure stigma and discrimination. |
| Hanisch et al., 2016 | Background: The majority of people experiencing mental-health problems do not seek help, and the stigma of mental illness is considered a major barrier to seeking appropriate treatment. More targeted interventions (e.g. at the workplace) seem to be a promising and necessary supplement to public campaigns, but little is known about their effectiveness. The aim of this systematic review is to provide an overview of the evidence on the effectiveness of interventions targeting the stigma of mental illness at the workplace. Methods: Sixteen studies were included after the literature review. The effectiveness of anti-stigma interventions at the workplace was assessed by examining changes in: (1) knowledge of mental disorders and their treatment and recognition of signs/symptoms of mental illness, (2) attitudes towards people with mental-health problems, and (3) supportive behavior. Results: The results indicate that anti-stigma interventions at the workplace can lead to improved employee knowledge and supportive behavior towards people with mental-health problems. The effects of interventions on employees’ attitudes were mixed, but generally positive. The quality of evidence varied across studies. Conclusions: This highlights the need for more rigorous, higher-quality evaluations conducted with more diverse samples of the working population. Future research should explore to what extent changes in employees’ knowledge, attitudes, and supportive behavior lead to affected individuals seeking help earlier. Such investigations are likely to inform important stakeholders about the potential benefits of current workplace anti-stigma interventions and provide guidance for the development and implementation of effective future interventions. |
| Heim et al., 2018 | Aims. This systematic review compiled evidence on interventions to reduce mental health- related stigma in primary health care (PHC) in low- and middle-income countries (LMICs). Studies targeting PHC staff (including non-professionals) were included. Primary outcomes were stigmatising attitudes and discriminatory behaviours. Methods. Data collection included two strategies. First, previous systematic reviews were searched for studies that met the inclusion criteria of the current review. Second, a new search was done, covering the time since the previous reviews, i.e. January 2013 to May 2017. Five search concepts were combined in order to capture relevant literature: stigma, mental health, intervention, PHC staff and LMICs. A qualitative analysis of all included full-texts was done with software MAXQDA. Full-texts were analysed with regards to the content of interventions, didactic methods, mental disorders, cultural adaptation, type of outcome measure and primary outcomes. Furthermore, a risk of bias assessment was undertaken. Results. A total of 18 studies were included. Risk of bias was rated as high in most included studies. Only six studies had tested their intervention against a control condition, two of which had used random allocation. Most frequently used interventions were lectures providing theoretical information. Many studies also used interactive methods (N = 9), discussed case studies (N = 8) or used role plays (N = 5). Three studies reported that they had used clinical practice and supervision. Results of these studies were mixed. No or little effects were found for brief training interventions (e.g. 1 h to 1 day). Longer training interventions with more sophisticated didactic methods produced statistically significant changes in validated stigma questionnaires. These results have to be interpreted with caution due to risk of bias. Methods for cultural adaptation of interventions were rarely documented. Conclusions. More rigorous trials are needed in LMICs to test interventions that target discriminatory behaviours in relationship with patients. Cultural adaptation of stigma interventions and structural/institutional factors should be more explicitly addressed in such trials. |
| Ma et al, 2019 | Stigma is a primary concern for people living with human immunodeficiency virus (HIV)/AIDS (PLWHA), and has great impact on their and their family members’ health. While previous reviews have largely focused on the public stigma, this systematic review aims to evaluate the impact of HIV/acquired immunodeficiency syndrome (AIDS)-related self-stigma reduction interventions among PLWHA and their families. A literature search using eight databases found 23 studies meeting the inclusion criteria. Five types of intervention approaches were identified: (1) psycho-educational intervention, (2) supportive intervention for treatment adherence (antiretroviral therapy), (3) psychotherapy intervention, (4) narrative intervention, and (5) community participation intervention. Overall, the reviewed articles suggested a general trend of promising effectiveness of these interventions for PLWHA and their family members. Psycho-educational interventions were the main approach. The results highlighted the need for more interventions targeting family members of PLWHA, and mixed-methods intervention studies. |
| Mehta et al., 2015 | Background: Most research on interventions to counter stigma and discrimination has focused on short-term outcomes and has been conducted in high-income settings.  Aims: To synthesise what is known globally about effective interventions to reduce mental illness-based stigma and discrimination, in relation first to effectiveness in the medium and long term (minimum 4 weeks), and second to interventions in low- and middle-income countries (LMICs).  Method: We searched six databases from 1980 to 2013 and conducted a multi-language Google search for quantitative studies addressing the research questions. Effect sizes were calculated from eligible studies where possible, and narrative syntheses conducted. Subgroup analysis compared interventions with and without social contact.  Results: Eighty studies (n = 422 653) were included in the review. For studies with medium or long-term follow-up (72, of which 21 had calculable effect sizes) median standardised mean differences were 0.54 for knowledge and −0.26 for stigmatising attitudes. Those containing social contact (direct or indirect) were not more effective than those without. The 11 LMIC studies were all from middle-income countries. Effect sizes were rarely calculable for behavioural outcomes or in LMIC studies.  Conclusions: There is modest evidence for the effectiveness of anti-stigma interventions beyond 4 weeks follow-up in terms of increasing knowledge and reducing stigmatising attitudes. Evidence does not support the view that social contact is the more effective type of intervention for improving attitudes in the medium to long term. Methodologically strong research is needed on which to base decisions on investment in stigma-reducing interventions. |
| Mills et al., 2020 | People with mental health problems often experience self-stigma, whereby they internalise stereotypic or stigmatising views held by others. Self-stigma is known to have negative effects on self-esteem and self-efficacy and a continuing impact on psychological wellbeing. Self-help interventions designed to reduce self-stigma may have an important contribution to make. This review aimed to provide an overview and critical appraisal of the literature on self-help interventions that target self-stigma related to mental health problems. A systematic review of five electronic databases (PsycINFO, MEDLINE, CINAHL Plus, Scopus and EMBASE) was carried out to identify articles published between January 2007 and July 2019. Eight articles that reported on self-help interventions for self-stigma were identified and evaluated using a combination of quality appraisal and narrative synthesis. |
| Pantelic et al, 2019 | This systematic review aims to synthesise evidence on predictors of internalised HIV stigma amongst people living with HIV in sub-Saharan Africa. Preferred Reporting Items for Systematic Reviews and Meta-Analyses guidelines were used. Studies were identified through electronic databases, grey literature, reference harvesting and contacts with key researchers. Quality of findings was assessed through an adapted version of the Cambridge Quality Checklists. A total of 590 potentially relevant titles were identified. Seventeen peer-reviewed articles and one draft book chapter were included. Studies investigated socio-demographic, HIV-related, intra-personal and interpersonal correlates of internalised stigma. Eleven articles used cross-sectional data, six articles used prospective cohort data and one used both prospective cohort and cross-sectional data to assess correlates of internalised stigma. Poor HIV-related health weakly predicted increases in internalised HIV stigma in three longitudinal studies. Lower depression scores and improvements in overall mental health predicted reductions in internalised HIV stigma in two longitudinal studies, with moderate and weak effects, respectively. No other consistent predictors were found. Studies utilising analysis of change and accounting for confounding factors are necessary to guide policy and programming but are scarce. High-risk populations, other stigma markers that might layer upon internalised stigma, and structural drivers of internalised stigma need to be examined |
| Sermrittirong et al, 2014: | B**ackground:**Interventions to reduce leprosy-related stigma, to reduce the manifestations of stigma that cause so much suffering to individuals and their families.  **Purpose:**This systematic review aims to identify interventions that have been used to reduce such stigma and to summarise what is known about their efficacy.  **Method:**Electronic searches were undertaken using PubMed (Medline), CINAHL and PsycInfo databases. The internet was searched using Google Scholar for papers not published in these databases. All relevant papers written in Thai or English were included.  **Results:**After reading 55 papers, three duplications and parallel literatures were removed, 18 were removed on abstract screening and nine after reading the full papers; eventually, 25 were included in this review. Interventions with some evidence of effectiveness in terms of stigma reduction comprise the integration of leprosy programmes into general health care; Information Education and Communication (IEC) programmes; and socio-economic rehabilitation.  **Conclusion:**More evaluations are needed of the effect of the integration of leprosy programmes into general health services. The design and implementation of IEC interventions need to be preceded by careful study of the target area and population and should be undertaken in combination with other activities. |
| Sommerland et al, 2017 | Background: While substantial progress is being made in tuberculosis (TB) control, the success of public health efforts is hampered by pervasive stigma.  Objective: To perform a systematic literature review to assess the effectiveness of interventions aimed at reducing TB stigma in patients, health care workers, care givers and the general community.  Design: Studies were eligible for inclusion if they evaluated interventions aimed at reducing TB stigma and were published between 1950 and 2015. We searched eight databases (PubMed, Cochrane Library, Ovid, Embase, PsycInfo, Sociological Abstracts, Cumulative Index to Nursing and Allied Health Literature, World Health Organization Latin American and Caribbean Health Sciences Literature), and complemented the searches by using the snowball strategy and by reviewing relevant grey literature.  Results: Only seven studies were identified as providing quantitative (n = 4) or qualitative (n = 3) evidence of effectiveness in reducing TB stigma. Quality assessment of the studies was poor. Knowledge-shaping and attitude-changing interventions aimed at the public, patients and their families were effective in reducing anticipated stigma. Home visits and support groups were effective in reducing both anticipated and internalised stigma.  Conclusion: There is a dearth of reliable information on the effectiveness of TB stigma-reduction interventions. Knowledge-shaping, attitude-changing and patient-support interventions can be effective in reducing TB stigma, but more rigorous evaluations are needed. |

### SUMMARY Table: evidence articles

| **First author,**  **year** | **Type of clinical condition** | **Type of evidence** | **Population** | **Intervention** | **Outcome** | **Results** | **Comment** |
| --- | --- | --- | --- | --- | --- | --- | --- |
| Andersson et al, 2019 | **HIV/AIDS** | Systematic review; n=27 studies  (quasi-experimental studies, qualitative or mixed methods studies, trials and pre-/post-test studies) | People living with HIV | Different stigma- reducing interventions | Stigma amongst affected people (enacted, anticipated, internalised) | Group based behavioural intervention, patient centred mental health programme and community support initiative showed to reduce stigma | There is a scarcity of well designed intervention studies that document stigma reduction in people living with HIV and few studies that specifically assess the effect of stigma on health-related quality of life. |
| Büchter et al.,2017 | **Mental illness** | Systematic review; n=5 studies  (randomised controlled trials /RCTs) | People with mental illness | interventions aimed at reducing self-stigma  Included various group based anti- stigma interventions and an anti-stigma booklet. | Self-stigma | 4 of 5 included studies had high risk of bias.  The meta-analysis did not find a statistically significant effect. None of the individual studies found sustainable effects on other outcomes, including recovery, help-seeking behaviour and self-stigma. | The effectiveness of interventions against self-stigma is uncertain.  But it should not be concluded that interventions aimed at reducing self-stigma in people with mental illnesses are ineffective. The sparse evidence and a number of methodological reasons that reside in the limitation of the included studies might ex- plain the disappointing results. |
| Clement et al., 2013 | **Mental illness** | Systematic review; n=22 studies  (RCTs, cluster RCTs, or interrupted time series studies) | General public  (or any of its constituent groups, e.g. occupational or sociodemographic groups or any other target group) | Mass media interventions on reducing stigma (discrimination and prejudice) related to mental ill health compared to inactive controls | Stigma, discrimination and prejudice  Secondary outcomes: knowledge, cost, reach, recall, and awareness of interventions, duration/sustainability of media effects, audience reactions to media content, and unforeseen adverse effects. | Mass media interventions may reduce prejudice in the immediate, short and medium term.  Overall, the extent to which they do this may be considered small‐to‐medium, roughly equivalent to reducing the level of prejudice from that associated with symptoms of schizophrenia to the level associated with symptoms of major depression, and mainly rated as ‘quite important’ by consumers. | Some types of intervention had larger beneficial effects. Consequently, there is justification for continuing to use mass media as one strategy for countering the stigma associated with mental ill health.  As mass media interventions have the potential to reach large numbers of people, even small benefits may have important effects at the population level.  Mass media interventions were able to increase knowledge and were well received in the small number of studies which assessed these outcomes. |
| Feyissa et al, 2019 | **HIV/AIDS** | Systematic review; n=14 records reporting on 8 studies  (all studies with comparative designs; e.g. RCTs, quasi-experimental, pre-post design) | Healthcare workers | Information based approach  Skills building interventions (demonstration and role playing)  Structural interventions  Contact strategies  Biomedical interventions  Counselling and support interventions | Stigma and discrimination related to HIV amongst healthcare workers, and institutional factors | Training popular opinion leaders (POLs) resulted in significantly lower mean avoidance intent scores (MD = -1.87 [95% CI -2.05 to -1.69]), mean prejudicial attitude scores (MD = -3.77 [95% CI -5.4 to -2.09]) and significantly higher scores in mean compliance to universal precaution (MD = 1.65 [95% CI 1.41 to 1.89]) when compared to usual care. | Evidence of moderate quality indicates that training popular opinion leaders is effective in reducing avoidance intent and prejudicial attitude and improving compliance to universal precaution.  Very low quality evidence indicates that professionally-assisted peer group interventions, modular interactive training, participatory self-guided assessment and intervention, contact strategy combined with information giving and empowerment are effective in reducing HIV-related stigma |
| Hanisch et al., 2016 | **Mental illness** | Systematic review; n=16 studies  (RCTs and quasi-experimental studies) | People in the workplace (not people with mental illness in workplace) | interventions targeting stigma against mental illness at the workplace | Public stigma; in workplace | Workplace anti-stigma interventions can be particularly effective in changing employees’ knowledge of mental disorders, as well as helping behaviour, while results related to attitudinal change were mixed, but positive overall.  Ten anti-stigma interventions were shown to be effective in increasing mental-health knowledge with one exception.  Six studies with high risk of bias had a positive impact on mental-health literacy. Although evidence on effectiveness of interventions on changing attitudes was mixed, nine (of 14) studies did report improvements in participants’ stigmatizing attitudes.  All types of anti-stigma interventions in 11 studies consistently had a significant positive impact on employees’ supportive behaviour with the exception of one study, which reported a marginally significant effect. | *>> However, due to methodological shortcomings in the majority of the included studies, the lack of follow-ups beyond post- intervention assessments, as well as heterogeneity in terms of intervention content, duration, and outcome measures, the evidence for the effectiveness of workplace anti-stigma interventions is inconclusive and must be interpreted with caution.*  *>> The development and implementation of effective anti-stigma programs specifically designed for the workplace is, however, of high importance.*  1 -Tailored strategies targeting the workplace might prove a more promising route to stigma change than public campaigns; more people might be reached effectively via more targeted interventions (e.g. at work).  2 - participation in anti-stigma programs, for example in the scope of personnel development, could be made mandatory in an organizational setting, whereas public stigma campaigns require people to participate voluntarily.  3 - exposure to mass-media approaches to stigma change can be short in time, whereas workplace interventions can be more intensive in terms of length and information. |
| Heim et al., 2018 | **Mental illness** | Systematic review; n=18 studies  (qualitative or quantitative studies, no comparator defined) | Primary health care (PHC) staff (including non-professionals) in Low Middle Income Countries | Mental health-related training programme addressing PHC workers’ knowledge, attitudes and behaviours in terms of professional skills.  (providing theoretical information through lectures was the most frequent intervention, and more practical interventions targeting discriminatory behaviours, such as role plays or clinical practice under supervision, were rarely used) | Stigma,  in terms of “PHC workers’ knowledge, attitudes (stigma-related) and behaviours in terms of professional skills” | Most studies found some kind of positive effects of their intervention on attitudes of PHC staff towards people with mental illness, but some of these effects were rather small.  No or little effects were found from short training interventions, e.g. a 1-h training on depression screening, or a one-session stigma intervention for general practitioners | Quality of included studies varied greatly, with a high number of studies showing a high risk of bias.  *>>> From the present state of the literature, no meaningful conclusions can be drawn on the effectiveness of stigma interventions, key ingredients or target populations within health care.*  all included studies measured outcomes by assessing knowledge and attitudes, with only a few studies measuring behavioural outcomes.  *>>>* few of the included studies have made an attempt to culturally adapt their interventions .*.. most likely that stigma interventions require procedures for cultural adaptation, since how stigma is experienced varies across cultures* |
| Ma et al, 2019 | **HIV/AIDS** | Systematic review; n=23 studies  (experimental, quasi-experimental, or pre-post design) | People living with HIV/AIDS (PLWHA) and their families | Psycho-educational intervention  Supportive intervention for treatment adherence (antiretroviral therapy)  Psychotherapy intervention  Narrative intervention  Community participation intervention | Self-stigma  Secondary outcomes: depression, self-efficacy, self-esteem, anxiety and stress. | General trend of promising effectiveness of these interventions for PLWHA and their family members. Psycho-educational interventions were the main approach. | The heterogeneity of the study, coupled with insufficient data for the measurement of effect size, constrained the ability to draw conclusions on the most effective interventions.  The evidence suggests that there is a lack of interventional research targeting family members of PLWHA. |
| Mehta et al., 2015(q1) | **Mental illness** | Systematic review; Question 1 n=72 studies  (all quantitative study designs (incl. RCTs), reporting comparison with a control group or a baseline comparator) | Any  (But most interventions were in HIC settings (93%), an aimed at students (37%)) | Any type of intervention with a stated aim of changing mental health-related stigma or with an implied aim of changing stigma | Stigma  (any type of stigma reduction, long-term effectiveness) | At follow-up at 4 weeks or longer, interventions aimed at reducing mental health-related stigma typically had a medium-sized effect on knowledge outcomes and a small effect on attitudinal outcomes, although for both types of outcome statistically non- significant findings were as common as significant ones.  There were insufficient data on behavioural outcomes to draw any conclusions on the medium- or long-term effectiveness of interventions to reduce discrimination. | *>> findings did not support the superiority of social contact interventions as we had expected.*  As it is vital that stigma reduction is sustained in the longer term, the effectiveness of such social contact interventions clearly warrants further research.  *>> although social contact appears to be the most strongly evidence-based type of intervention to reduce stigma when measured by immediate post-intervention outcomes, there is not at present evidence to show that such immediate benefits persist in the longer term* |
| Mehta et al., 2015(q2) | **Mental illness** | Systematic review; Question 2 n=11 studies  (all quantitative study designs (incl. RCTs), reporting comparison with a control group or a baseline comparator) | Any  (Six studies were aimed at school and university students, two at caregivers of people with schizophrenia, and three at healthcare professionals.)  in Low-Middle Income Countries | Any type of intervention with a stated aim of changing mental health-related stigma or with an implied aim of changing stigma | Stigma  (any type of stigma reduction, long-term effectiveness) | *Effect size* could only be calculated for one study: large effect size for improving stigmatising attitudes.  *Comparing significant and non-significant findings* >>   - No statistically significant improvements in knowledge reported. - Significant reductions in stigmatising attitudes in 11 or 12 scale outcomes assessed. - No studies assessed behavioural outcomes (discrimination). | No studies from low-income countries, all from middle-income.  *>> There is a clear need for more stigma reduction studies, particularly from low-income countries.*  *>> results should be interpreted with caution, low methodological quality or unclear risk of bias* |
| Mills et al., 2020 | **Mental illness** | Systematic review; n=8 studies  (quantitative or qualitative methodologies) | People with mental illness | self-help interventions health problems  (seven: web-based interventions, “primarily” psychoeducation; one non-web-based intervention, self-help workbook) | Self-stigma | Self-help interventions can be of benefit in reducing self-stigma, specifically depression personal stigma, and self-stigma related to help-seeking. However, the results were mixed for other areas of personal stigma, such as anxiety, suicide, and PTSD | Only one included study had outcome explicitly measuring self-stigma, others included broader stigma measures (but all studies targeted self-stigma]  Limited evidence identified |
| Pantelic et al, 2019 | **HIV/AIDS** | Systematic review; n=20 studies  (RCTs, controlled clinical trials, prospective and retrospective controlled cohort studies, controlled pre-post studies, interrupted time series) | People living with HIV and key populations affected by HIV in Low-Middle Income Countries | Studies were clustered into four categories based on the socioecological level of risk or resilience that they targeted: (1) individual level only, (2) individual and relational levels, (3) individual and structural levels and (4) structural level only**.** | Self-stigma  Multidimensional stigma outcome measure that differentiates between self-stigma and other types of stigma | Structural interventions such as scale-up of antiretroviral treatment, prevention of medication stockouts, social empowerment and economic strengthening may help substantially reduce self-stigma among individuals |  |
| Sermrittirong et al, 2014: | **Leprosy** | Systematic review; n=25 studies  (qualitative, quantitative, or mixed methods designs) | General public, medical staff, patient with leprosy | Integration of leprosy services in general health care;  Information, Education and Communication (IEC) programmes;  Socio-economic rehabilitation (SER);  Changing the name of the disease;  Counselling | Stigma | Interventions with at least some evidence of success in reducing stigma are the integration of a leprosy programme into general health care, and IEC and SER interventions. | To collect additional evidence of the effectiveness of the interventions, particularly where the leprosy programme has already been integrated into general health care, stigma should also be included in programme evaluation objectives. IEC interventions should be preceded by careful study of the target area and population. They should be combined with other stigma reduction strategies to achieve an optimal effect. |
| Sommerland et al, 2017 | **Tuberculosis (TB**) | Systematic review; n=7 studies  (RCTs, quasi-experimental studies with longitudinal or cross-sectional designs, qualitative studies, mixed-methods studies) | General public,  People with TB  Care givers , including health care workers | Psychosocial support groups  Awareness raising by community volunteers  Community nursing  Health education programme  TB clubs: each club had regular weekly  TB clubs, home visits  Educational workshops | Stigma:  anticipated stigma  internalized stigma | The support groups were effective against the negative social impact faced by MDR-TB patients | Main findings: lack of reliable information on evidence-based interventions for anticipated and internalised stigma, and no evidence-based intervention for enacted stigma.  There is a lack of rigorous evaluation of stigma-reduction interventions and interventions found to be beneficial have rarely been repeated (lack of proven replicability)  >>support groups and conscious focus on improving attitudes in the general community, showed promising results in reducing both internalised and anticipated stigma  >>potential synergies in simultaneously engaging a range of populations, such as TB patients, their families, and HCWs, in multivalent stigma interventions |
|  | **COVID-19** | No systematic reviews, no primary studies were found in the search | | | | | |
|  | **SARS/MERS** | No systematic reviews, no primary studies were found in the search | | | | | |
|  | **INFLUENZA** | No systematic reviews, no primary studies were found in the search | | | | | |
|  | **EBOLA** | No systematic reviews, no primary studies were found in the search | | | | | |

## Additional materials: narrative description or abstract

### STIGMA and COVID-19

WHO, 2020a

WHO, UNICEF and the International Federation of Red Cross and Red Crescent Societies (IFRC) developed a community-based guide to preventing and addressing social stigma associated with COVID-19 targeted at government, media and local organisations working on the new coronavirus disease (COVID-19) (WHO, 2020).

The level of stigma associated with COVID-19 is based on three main factors: 1) it is a disease that’s new and for which there are still many unknowns; 2) we are often afraid of the unknown; and 3) it is easy to associate that fear with ‘others’. It is understandable that there is confusion, anxiety, and fear among the public. Unfortunately, these factors are also fuelling harmful stereotypes.

Stigma can undermine social cohesion and prompt possible social isolation of groups, which might contribute to a situation where the virus is more, not less, likely to spread. This can result in more severe health problems and difficulties controlling a disease outbreak.

Stigma can:

- Drive people to hide the illness to avoid discrimination
- Prevent people from seeking health care immediately
- Discourage them from adopting healthy behaviours

How we communicate about COVID-19 is critical in supporting people to take effective action to help combat the disease and to avoid fuelling fear and stigma. An environment needs to be created in which the disease and its impact can be discussed and addressed openly, honestly and effectively.

Here are some tips on how to address and avoid compounding, social stigma:

1. Words matter: dos and don’ts when talking about the new coronavirus (COVID-19)
2. Do your part: simple ideas to drive stigma away
3. Communication tips and messages.

WORDS MATTER:

When talking about coronavirus disease, certain words (i.e. suspect case, isolation…) and language may have a negative meaning for people and fuel stigmatizing attitudes. They can perpetuate existing negative stereotypes or assumptions, strengthen false associations between the disease and other factors, create widespread fear, or dehumanise those who have the disease.

This can drive people away from getting screened, tested and quarantined. We recommend a ‘people-first’ language that respects and empowers people in all communication channels, including the media. Words used in media are especially important, because these will shape the popular language and communication on the new coronavirus (COVID-19). Negative reporting has the potential to influence how people suspected to have the new coronavirus (COVID-19), patients and their families and affected communities are perceived and treated.

There are many concrete examples of how the use of inclusive language and less stigmatizing terminology can help to in control epidemics and pandemics from the HIV, TB and H1N1 Flu.

DOS and DON'TS

Below are some dos and don'ts on language when talking about the new coronavirus disease (COVID-19):

DO - talk about the new coronavirus disease (COVID-19)

Don’t - attach locations or ethnicity to the disease, this is not a “Wuhan Virus”, “Chinese Virus” or “Asian Virus”.

DO - talk about “people who have COVID-19”, “people who are being treated for COVID-19”, “people who are recovering from COVID-19” or “people who died after contracting COVID-19”

Don’t - refer to people with the disease as “COVID-19 cases” or “victims”

DO - talk about “people who may have COVID-19” or “people who are presumptive for COVID-19”

Don’t - talk about “COVID-19 suspects” or “suspected cases”.

DO - talk about people “acquiring” or “contracting” COVID-19

Don’t - talk about people “transmitting COVID-19” “infecting others” or “spreading the virus” as it implies intentional transmission and assigns blame. Using criminalising or dehumanising terminology creates the impression that those with the disease have somehow done something wrong or are less human than the rest of us, feeding stigma, undermining empathy, and potentially fuelling wider reluctance to seek treatment or attend screening, testing and quarantine.

DO - speak accurately about the risk from COVID-19, based on scientific data and latest official health advice.

Don’t - repeat or share unconfirmed rumours, and avoid using hyperbolic language designed to generate fear like “plague”, “apocalypse” etc.

DO - talk positively and emphasise the effectiveness of prevention and treatment measures. For most people this is a disease they can overcome. There are simple steps we can all take to keep ourselves, our loved ones and the most vulnerable safe.

Don’t - emphasise or dwell on the negative, or messages of threat. We need to work together to help keep those who are most vulnerable safe.

DO - emphasise the effectiveness of adopting protective measures to prevent acquiring the new coronavirus, as well as early screening, testing and treatment.

DO YOUR PART:

Governments, citizens, media, key influencers and communities have an important role to play in preventing and stopping stigma surrounding, for example of people from China and Asia in general. We all need to be intentional and thoughtful when communicating on social media and other communication platforms, showing supportive behaviours around the new coronavirus disease (COVID-19).

Here are some examples and tips on possible actions to counter stigmatizing attitudes:

- Spreading the facts: Stigma can be heightened by insufficient knowledge about how the new coronavirus disease (COVID-19) is transmitted and treated, and how to prevent infection. In response, prioritise the collection, consolidation and dissemination of accurate country- and community-specific information about affected areas, individual and group vulnerability to COVID-19, treatment options and where to access health care and information. Use simple language and avoid clinical terms. Social media is useful for reaching a large number of people with health information at relatively low cost.
- Engaging social influencers such as religious leaders on prompting reflection about people who are stigmatized and how to support them, or respected celebrities to amplify messages that reduce stigma. The information should be well targeted and the celebrities who are asked to communicate this information must be personally engaged, and geographically and culturally appropriate to the audiences they seek to influence. An example would be a mayor (or another key influencer) going live on social media and shaking hands with the leader of the Chinese community.
- Amplify the voices, stories and images of local people who have experienced the new coronavirus (COVID-19) and have recovered or who have supported a loved one through recovery to emphasise that most people do recover from COVID-19. Also, implementing a “hero” campaign honouring caretakers and healthcare workers who may be stigmatized. Community volunteers also play a great role in reducing stigma in communities.
- Make sure you portray different ethnic groups. All materials should show diverse communities being impacted and working together to prevent the spread of COVID-19. Ensure that typeface, symbols and formats are neutral and don’t suggest any particular group.
- Ethical journalism: Journalistic reporting which overly focuses on individual behaviour and patients’ responsibility for having and “spreading COVID-19” can increase stigma of people who may have the disease. Some media outlets have, for example, focused on speculating on the source of COVID-19, trying to identify “patient zero” in each country. Emphasizing efforts to find a vaccine and treatment can increase fear and give the impression that we are powerless to halt infections now. Instead, promote content around basic infection prevention practices, symptoms of COVID-19 and when to seek health care.
- Link up: There are a number of initiatives to address stigma and stereotyping. It is key to link up to these activities to create a movement and a positive environment that shows care and empathy for all.

COMMUNICATION TIPS and MESSAGES

An “infodemic” of misinformation and rumours is spreading more quickly than the current outbreak of the new coronavirus (COVID-19). This contributes to negative effects including stigmatization and discrimination of people from areas affected by the outbreak. We need collective solidarity and clear, actionable information to support communities and people affected by this new outbreak.

Misconceptions, rumours and misinformation are contributing to stigma and discrimination which hamper response efforts.

- Correct misconceptions, at the same time as acknowledging that people’s feelings and subsequent behaviour are very real, even if the underlying assumption is false.
- Promote the importance of prevention, lifesaving actions, early screening and treatment.

Collective solidarity and global cooperation are needed to prevent further transmission and alleviate the concerns of communities.

- Share sympathetic narratives, or stories that humanize the experiences and struggles of individuals or groups affected by the new coronavirus (COVID-19)
- Communicate support and encouragement for those who are on the frontlines of response to this outbreak (health care workers, volunteers, community leaders etc).

Facts, not fear will stop the spread of novel coronavirus (COVID-19)

- Share facts and accurate information about the disease.
- Challenge myths and stereotypes.
- Choose words carefully. The way we communicate can affect the attitudes of others (see do’s and don’ts above).

WHO, 2020b

Mental health and psychosocial considerations during the COVID-19 outbreak.

In January 2020 the World Health Organization (WHO) declared the outbreak of a new coronavirus disease, COVID-19, to be a Public Health Emergency of International Concern. WHO stated that there is a high risk of COVID-19 spreading to other countries around the world. In March 2020, WHO made the assessment that COVID-19 can be characterized as a pandemic. WHO and public health authorities around the world are acting to contain the COVID-19 outbreak. However, this time of crisis is generating stress throughout the population. The considerations presented in the document have been developed by the WHO Department of Mental Health and Substance Use as a series of messages that can be used in communications to support mental and psychosocial well-being in different target groups during the outbreak: 1 Messages for the general population; 2. Messages for healthcare workers; 3. Messages for team leaders or managers in health facilities; 4. Messages for carers of children; 5. Messages for older adults, people with underlying health conditions and their carers; 6. Messages for people in isolation

Nature, 2020

The pandemic is fuelling deplorable racism, especially against Asian people. Education and research will also pay the price.

When the World Health Organization (WHO) announced in February that the disease caused by the new coronavirus would be called COVID-19, the name was quickly adopted by organizations involved in communicating public-health information. As well as naming the illness, the WHO was implicitly sending a reminder to those who had erroneously been associating the virus with Wuhan and with China in their news coverage— including Nature. That we did so was an error on our part, for which we take responsibility and apologize. For years, it was common for viral diseases to be associated with the landscapes, places or regions where the first outbreaks occurred — as in Middle East respiratory syndrome, or Zika virus, named after a forest in Uganda. But in 2015, the WHO introduced guidelines to stop this practice and thereby reduce stigma and negative impacts such as fear or anger directed towards those regions or their people. The guidelines underlined the point that viruses infect all humans: when an outbreak happens, everyone is at risk, regardless of who they are or where they are from. And yet, as countries struggle to control the spread of the new coronavirus, a minority of politicians are sticking with the outdated script. US President Donald Trump has repeatedly associated the virus with China. Brazilian lawmaker Eduardo Bolsonaro — the son of President Jair Bolsonaro — has called it “China’s fault”. Politicians elsewhere, including in the United Kingdom, are also saying that China bears responsibility. Continuing to associate a virus and the disease it causes with a specific place is irresponsible and needs to stop. As infectious-disease epidemiologist Adam Kucharski reminds us in his timely book The Rules of Contagion, published in February, history tells us that pandemics lead to communities being stigmatized, which is why we all need to exercise more care. If in doubt, seek advice, and always fall back on the consensus of the evidence.

Racist attacks

Failing to do so has consequences. It’s clear that since the outbreak was first reported, people of Asian descent around the world have been subjected to racist attacks, with untold human costs — for example, on their health and livelihoods. Law-enforcement agencies say they are making investigation of hate crimes a high priority, but such inquiries might come too late for some, including many of the more than 700,000 Chinese undergraduate, master’s and PhD students studying at universities outside China. The majority are in Australia, the United Kingdom and the United States. Many have returned home while their institutions are closed owing to lockdowns, and many might not return. Students are hesitating to come back, in part because of fears of continuing racism, along with uncertainty over the future of their courses and not knowing when international travel will resume. These young people will experience disruption and the loss of new connections and opportunities. But the loss of students from China and other countries in Asia has wide-ranging -and worrying implications for the scholarly enterprise, too. It means that universities in the affected countries will become less diverse — something that has not happened for generations.

A loss for all

For decades, campuses have striven to boost diversity, and countries have enacted policies to encourage international academic mobility. Diversity is valuable for its own sake. It encourages understanding and dialogue between cultures, and sharing of points of view and ways of being. And it has always been a fuel for research and innovation. Moreover, a diverse campus body is needed to improve policies and structures so that universities — and research publishing — can become more welcoming. Many barriers to diversity remain: in the April issue of Nature Reviews Physics, for example, researchers and science communicators from China, India, Japan and South Korea report examples of discrimination and other factors that prevent them being heard in international journals (S. Hanasoge et al. Nature Rev. Phys. 2, 178–180; 2020). Many leaders want to listen to and act on expert scientific advice to deal with this pandemic and save lives. On terminology, the advice is clear: we must all do everything we can to avoid and reduce stigma; not associate COVID-19 with particular groups of people or places; and emphasize that viruses do not discriminate — we are all at risk. It would be tragic if stigma, fuelled by the coronavirus, led Asia’s young people to retreat from international campuses, curtailing their own education, reducing their own and others’ opportunities and leaving research worse off — just when the world is relying on it to find a way out. Coronavirus stigma must stop — now.

APA, 2020

History has shown that epidemics and pandemics tend to provoke xenophobia and stigma. This was the case with diseases such as Ebola and MERS and it is now the case with COVID-19. An increasing number of news reports document instances of stereotyping, harassment and bullying directed at people perceived to be of Asian descent following the spread of the new coronavirus. While the origin of the virus appears linked to a specific region of China, no one racial or ethnic group is at greater risk of infection or spread. Associating the coronavirus with China or specific regions within China — for example, through references to the “China virus” or “Wuhan virus” — helps engender biases and xenophobia. That’s why the World Health Organization strongly recommends against linking communicable diseases to specific geographic regions and recommends that all governments refrain from their use.

Why is addressing stigma so important?

Decades of research show discrimination is associated with poorer health and mental health among LGBTQ, Asian American, African American, American Indian, Alaska Native, Muslim American and Latinx populations. Stigmatized groups are particularly vulnerable during epidemics and pandemics — and it can put them and others at increased risk. That’s because stigma can lead people to hide symptoms of illness to avoid discrimination. They may not seek health care when they need it and may further isolate themselves, which comes with its own health risks.

In addition, stigmatized groups are more likely to be un- or underinsured, to have difficulty accessing culturally appropriate care, and to face bias in health-care systems, all of which ultimately compound the difficulty of containing the spread of viruses.

What you can do

The World Health Organization recommends that governments, citizens, media, key influencers and communities take steps to counter the contagion of bias and stigma, even as we collectively work to contain the spread of the virus. These steps include:

- Spread the facts: People are more susceptible to biases and stereotypes when they lack accurate information. Clear, concise and culturally appropriate communication — in multiple forms and in multiple languages — is needed to reach broad segments of the population, with particular focus on marginalized communities.
- Engage social influencers: Faith leaders, business leaders, elected officials and celebrities can be very effective in modelling appropriate communication and denouncing efforts to link epidemics with specific geographic areas and populations. It can be particularly effective for influencers to be visible having positive interactions with members of stigmatized groups.
- Amplify the voices of people with lived experience with coronavirus: Most people who contract the virus recover, and it can be reassuring to the public to hear their experiences, particularly when these individuals reflect the diversity of our communities. Similarly, honoring frontline care providers can reduce stigma against these groups.
- Portray different ethnic groups in public information materials: Images of diverse communities working together to reduce risk can powerfully communicate messages of solidarity and shared commitments to health and well-being. However, an outsized focus on Asian Americans in the case of COVID-19 could be harmful, so it’s important to guard against this.
- Promote ethical journalism: Media reports which focus on individual behavior and infected individuals’ “responsibility” for having and spreading the virus can stigmatize these individuals. News consumers should insist on responsible media reports that emphasize prevention practices, symptoms to look for and when to seek care.
- Correct myths, rumors and stereotypes, and challenge those whose language promotes bias: We all have a responsibility to correct stigmatizing language, and to challenge myths. No elected official should use language linking any epidemic to a particular people or geographic region, and challenging such language is a civic responsibility. Similarly, separating fact from rumor is important in an era when people are distrustful of information sources.

Bias, stigma and discrimination are public health threats. They damage the health, mental health and well-being of stigmatized groups. They also make it harder to contain current and future epidemics. We all share responsibility for good public health practice. Just as we know to wash our hands and maintain appropriate social distance, we should practice good behaviors when it comes to embracing and valuing diverse peoples and communities.

IASC, 2020

In any epidemic, it is common for individuals to feel stressed and worried. Some of fears and reactions associated with an epidemic spring from realistic dangers, but many reactions and behaviours are also borne out of lack of knowledge, rumours and misinformation.

Common responses of people affected (both directly and indirectly) might include fear of being socially excluded/placed in quarantine because of being associated with the disease (e.g. racism against people who are from, or perceived to be from, affected areas)

The constant fear, worry, uncertainties and stressors in the population during the COVID-19 outbreak can lead to long-term consequences within communities, families and vulnerable individuals: incl. stigma towards surviving patients resulting in rejection by communities.

Social stigma and discrimination can be associated with COVID-19, including towards people who have been infected, their family members and health care and other frontline workers. Steps must be taken to address stigma and discrimination at all phases of the COVID-19 emergency response. Care should be taken to promote the integration of people who have been affected by COVID-19 without over-targeting.

Risk communication is a key element of any outbreak. As an example, integrating positive mental health messages into all general public messages (TV, social media, etc.) will promote the wellbeing of the population. It is the responsibility of all sectors (e.g. health, social welfare, protection, education) including members of the media, to share such messages. A comprehensive public awareness campaign should be mobilized in order to educate communities, address stigma and discrimination and any excessive fears of contagion.

Frontline workers (including nurses, doctors, ambulance drivers, case identifiers, and others) may experience additional stressors during the COVID-19 outbreak:

due to e.g. stigmatization towards those working with COVID-19 patients and their remains, and reduced capacity to use social support due to intense work schedules and stigma within the community towards frontline workers.

Some frontline workers may experience ostracization by their family or community due to their fear and stigma; or indeed some families of responders may also be stigmatized and isolated from their community. This stigmatization can be detrimental to the mental wellbeing of affected people and can make an already challenging situation far more difficult (and may affect the morale of workers). During this time, it is important that the mental wellbeing of responders is protected. Engaging with community leaders is a key step to counteract such misconceptions. Peer support groups for responding health staff might also offer opportunities for other social support during the response, while sharing staff care information.

Strong emphasis needs to be placed on the strength and resourcefulness of communities rather than weaknesses and vulnerabilities. Special consideration should be made to ensure the protection of vulnerable groups, including (…) ethnic/cultural groups being targeted with stigma or discrimination.

GLOBALLY RECOMMENDED ACTIVITIES The list below outlines fourteen key activities that should be implemented as part of the response to COVID-19.

- #4: Establish a MHPSS strategy for COVID-19 cases, survivors, contacts (particularly those in isolation), family members, frontline workers and the broader community, with special attention to the needs of special or/and vulnerable groups. Ensure that the strategy addresses: fear, stigma, negative coping strategies (e.g. substance abuse) and the other needs identified through assessment and is building on positive, community- proposed coping strategies and promotes close collaboration between communities and health, education and social welfare services.

CDC, 2020

Center for Disease Control and Prevention (CDC) published a document on stigma related to COVID-19. Fear and disease about COVID-19 can lead to social stigma and discrimination and it can occur when people associate a disease with a population or nationality, even though not everyone in that population or from that region is specifically at risk for the disease. Stigma can also occur after a person has been released from COVID-19 quarantine even though they are not considered a risk for spreading the virus to others.

Some groups of people who may be experiencing stigma because of COVID-19 include:

- People of Asian descent
- People who have travelled
- Emergency responders or healthcare professionals

Stigma hurts everyone by creating fear or anger towards other people.

Stigmatized groups may be subjected to:

- Social avoidance or rejection
- Denials of healthcare, education, housing or employment
- Physical violence.

Stigma affects the emotional or mental health of stigmatized groups and the communities they live in. The document concluded that stopping stigma is important to making communities and community members resilient; everyone can help stop stigma related to COVID-19 by knowing the facts and sharing them with others in your community.

Earnshaw, 2020

Stigma has exacerbated the suffering from every major infectious disease epidemic in our history, and it will certainly play a role in the current COVID-19 pandemic. Stigma is an evolutionary response: We are hard-wired to physically distance ourselves from others who could infect us. We have an entire suite of evolved reactions, called “parasite avoidance,” to prevent us from maintaining contact with others who may carry communicable diseases. These reactions are what make us feel disgusted by signs of sickness, such as vomiting or skin lesions, whether or not these signs represent an actual threat to our own health.

There is a moral as well as physical component, as well. We tend to believe that bad things happen to bad people. This “just-world fallacy” tricks us into thinking that people who are infected with a disease may have done something wrong to deserve it. Maybe people who have become infected with COVID-19 didn’t wash their hands long enough, touched their face too much, or didn’t socially distance enough. This belief is comforting, helping us believe that we are in control of our own fate. It tells us that if we do everything right, we won’t become infected. Yet, we simply don’t live in a just world: We could do everything right, wash our hands for 60 seconds instead of just 20, and still become infected with COVID-19.

The Toll of Stigma

My decades of research show that stigma harms the mental and physical health of people with disease. This stigma can take the forms of social rejection, gossip, physical violence, and denial of services. Experiencing stigma from others can lead to elevated depressive symptoms, stress, and substance use. Alarmingly, people don’t have to experience stigma from others to be negatively affected by it. Just anticipating stigma from other people — perhaps because you’ve already seen sick people be ostracized or judged for their illness — can lead to anxiety and stress. Infected people may also internalize stigma, believing that they did something wrong or are a bad person because they became infected with a disease. The fact that many COVID-19 patients are medically isolated compounds the problem — patients under such separation orders have been shown to be at greater risk of distress.

Stigma does not only impact people who are sick, but extends to people who have an actual or perceived association with a disease. Family members of people with disease and healthcare providers caring for people with disease are at high risk of experiencing stigma from others during epidemics. In the context of COVID-19, stigma has additionally been directed at Asian Americans and people who have travelled to areas affected by the pandemic.

Stigmatizing anyone during a pandemic poses a threat to everyone. Research from HIV, Ebola, Hansen’s Disease, and other infectious disease epidemics shows that stigma undermines efforts at testing and treating disease. People who worry that they will be socially shunned if they are sick are less likely to get tested for a disease or seek treatment if they experience symptoms. Because of the just-world fallacy, they may also not believe that they could have a disease — after all, they’re a good person who has taken precautions to avoid the illness.

Social Distance, Not Social Isolation

The good news is that scientists working in diverse disease contexts have identified tools that can be leveraged to address stigma during COVID-19, including strategies for both reducing stigma and strengthening resilience, so that even if people are exposed to stigma they may not be as negatively affected by it.

Education is one of the most popular tools to deconstruct stigma. It can dispel harmful stereotypes, such as that Asian Americans are more likely to have COVID-19. Local and national leaders who fall ill to COVID-19 should be open about their diagnosis to help normalize the disease. When NBA star Magic Johnson announced he was HIV positive, HIV testing rates increased dramatically across the nation. In this regard, social media posts from celebrities who have the disease are also likely to help lift the taboo. I’ve been reminding colleagues and friends: If Tom Hanks and Rita Wilson can get COVID-19, we all can.

Corporate leaders can clarify that organizational values of inclusion, acceptance, and diversity extend to people who are affected by COVID-19. In some instances, enforcing anti-discrimination policies may be necessary. A patient who is fully recovered from COVID-19 is no longer infectious and should not be treated any differently from his or her colleagues. Corporate leaders can also create clear and confidential guidelines for reporting and responding to COVID-19 cases among employees, so that employees feel safe reporting if they become sick and secure that they will have a job when they have recovered. Organizations should also invest in wellness programs that promote resilience to stigma and other stressors. For example, mindfulness activities help to improve resilience to a wide range of stressors and there are a variety of platforms facilitating access to them.

While leadership is important, we all play a vital role in removing stigma during a pandemic. Indeed, one of our best reduction and resilience tools is simple social support. Employees can schedule virtual coffee hours, lunches, and happy hours with their co-workers to check in on them. We can call and send texts to our neighbours, especially those who have been sick, to update them on our lives and express hope of re-connection after social-distancing measures are lifted. We should also talk openly about the mental health struggles we are all facing — opportunities to talk with others about stressors including stigma can promote positive coping and mental well-being.

Although stigma is an evolved reaction to disease, it is not an inevitable one. Stigma divides and turns us against each other, but pandemics remind us of how connected we all are. Our shared vulnerability to this virus is a source of solidarity. We must remember that the virus — not people with COVID-19 or affected by COVID-19 — is the enemy.

Logie & Turan 2020

We are not being invaded. The body is not a battlefield. The ill are neither unavoidable casualties nor the enemy. We—medicine, society—are not authorized to fight back by any means whatever. – Sontag (1 p. 180)

Sontag’s quotation above from ‘AIDS and its metaphors’ reminds us to expose, and disengage from, constructions of illness that propagate fear. We are called to address the fear of COVID-19 by correcting misinformation. While misinformation is indeed a driver of fear and stigma, other underlying facilitators produce stigma and need to be considered in stigma mitigation. HIV research and an understanding of the historical construction of illness can be leveraged to mitigate COVID-19 stigma. COVID-19 public health responses—essential for prevention and containment —also have the potential to exacerbate stigma. We outline four tensions between COVID19 containment and stigma mitigation, and offer possible ways forward.

Tensions between COVID19 containment and stigma mitigation: 1) physical distancing, 2) travel restrictions, 3) misinformation, and 4) engaging affected communities.

1. Social distancing can lead to “othering” affected people, due to reduced interactions with them. However, social distancing is necessary public health guidance to slow the spread of COVID-19. Social distancing should not exacerbate othering, avoidance and mistreatment. Stigma messaging should reflect the evolving pattern of COVID-19 risk, to foster empathy and transform social distancing to “a normal and sustained practice” until pandemic is over. Building connections via kindness and caring can motivate uptake of non-stigmatising social distancing.
2. Travel bans and movement restrictions are necessary to help with virus confinement and to mitigate impact on overstretched health systems. But this can also facilitate stigma and xenophobia, and can serve to reinforce social hierarchies and power inequities. Enforced travel bans and movement restrictions might disproportionately affect already stigmatised people and vulnerable populations (e.g. homeless people, persons who are incarcerated, migrants and refugees, undocumented immigrants, and racial minorities. To counteract this, travel bans and quarantine could include anti-stigma and anti-xenophobia public messaging, and training of legal authorities. Instead of criminalising breaching COVID-19 related health policies, approaches should focus on empowering and strengthening communities and support persons to protect their own and others’ health.
3. Misinformation and lack of awareness around COVID-19 needs to be addressed. But this alone is not sufficient, it is necessary to also tackle facilitators like social inequities, including racism and xenophobia. Public health strategies to improve access to COVID-19 testing and employment sick leave benefits can reduce stigma. Addressing underlying health inequities is a long-term investment and process; tension between this and immediate work to provide information on COVID-19. Interventions should address both drivers (knowledge, misinformation) and facilitators (health policies, institutional practices) of stigma. Should harness momentum and act now to harness political investment in challenging social inequities that exacerbate impact of COVID-19 on marginalised communities.
4. It is necessary to engage with persons affected by COVID-19 in developing stigma mitigation strategies, but these persons may experience social and health disparities that constitute barriers to research participation. Lived experience of COVID-19 can inform contextually specific and stigma-informed public health policies. COVID-19 stigma might also intersect with other stigmas (e.g. gendered family roles), and social disparities are associated with health disparities. Syndemics approach could be useful to map out impact of social inequities on health issues, including COVID-19. Creative, web- based, and community-engaged strategies can aim to reduce participation barriers to involve persons most impacted by COVID-19 stigma in research and program development.

Asmundson, 2020

In a recent editorial the author discussed the concept of “coronaphobia”. Research on the psychological reactions to previous epidemics and pandemics suggests that various psychological vulnerability factors may play a role in coronaphobia, including individual difference variables such as the intolerance of uncertainty, perceived vulnerability to disease, and anxiety (worry) proneness. More research is needed to understand the relationship between coronaphobia and coronavirus-related xenophobia. Research from other outbreaks of infectious disease suggests that individual difference variables such as the perceived vulnerability to disease may play a role in both coronaphobia and coronavirus-related xenophobia. Likewise, lack of information and misinformation, often aided by sensational popular media headlines and foci, have been shown to fuel health-related fears and phobias. These factors may also play a significant role in coronaphobia. An important question is whether healthcare systems throughout the world are ready to deal with the surge of so-called “worried well” patients; that is, the surge into hospital emergency rooms of people who misinterpret their bodily sensations as signs of potential infection with the 2019-nCoV coronavirus. What has been lacking in the media, is a discussion of whether we are ready for a surge of patients into hospital emergency rooms whose problems are not coronavirus, but minor respiratory ailments combined with coronaphobia. The current outbreak of 2019-nCoV represents a call to action for psychosocial researchers and practitioners. It is vitally important to understand the psychosocial fallout of 2019-nCoV, such as excessive fear (or lack of concern and due caution) and discrimination, and to find evidence-based ways of addressing these issues. This will be important not only for 2019-nCoV, but also for future outbreaks of infection.

Chung-Ying Lin, 2020

In this editorial, the author highlighted that as the COVID‑19 outbreak is ongoing, a wave of fear and worry in the society has arisen. Fear and stigma toward the epidemics of COVID‑19 may lead to negative consequences of disease control, something prior SARS and Ebola outbreaks are vivid evidence of. Therefore, it has been advocated that there is a need to design an effective anti-stigma program that breaks the misperception in COVID‑19, increases the public’s knowledge about COVID‑19, and spreads encouraging positive and supportive messages.

Devakumar et al, 2020

Devakumar and colleagues found that the coronavirus disease 2019 (COVID-19) pandemic has uncovered social and political fractures within communities, with racialised and discriminatory responses to fear, disproportionately affecting marginalised groups. Rather than being an equaliser, given its ability to affect anyone, COVID-19 policy responses have disproportionately affected people of colour and migrants— people who are over-represented in lower socioeconomic groups, have limited health-care access, or work in precarious jobs. This is especially so in resource-poor settings that lack forms of social protection. Self-isolation is often not possible, leading to higher risk of viral spread. Ethnic minority groups are also at greater risk because of comorbidities. Furthermore, migrants, particularly those without documents, avoid hospitals for fear of identification and reporting, ultimately presenting late with potentially more advanced disease. Acts of discrimination occur within social, political, and historical contexts. Political leaders have misappropriated the COVID-19 crisis to reinforce racial discrimination, doubling down, for example, on border policies and conflating public health restrictions with antimigrant rhetoric. The strength of a health system is inseparable from broader social systems that surround it. Epidemics place increased demands on scarce resources and enormous stress on social and economic systems. Health protection relies not only on a well-functioning health system with universal coverage, but also on social inclusion, justice, and solidarity. In the absence of these factors, inequalities are magnified and scapegoating persists, with discrimination remaining long after. Division and fear of others will lead to worse outcomes for all.

Such a program can be designed with the use of social media, given the high access rate in relation to social media. Social media through the Internet allows people to communicate without time and space limits, and different platforms of the social media (e.g. Facebook, WhatsApp, LINE, WeChat, Twitter, Skype) have been well developed. With the correct information and knowledge posted on the social media, the fear and stigma are likely to be lowered. The author concludes that health‑care providers should think about potential programs to combat COVID‑19 misinformation, stigma, and fear.

### STIGMA and SARS

Person et al, 2004

The authors discussed SARS-related Fear, Stigmatization, and Discrimination. Fear of SARS arose from the underlying anxiety about a disease with an unknown cause and possible fatal outcome. Because of their evolving nature and inherent scientific uncertainties, outbreaks of emerging infectious diseases can be associated with considerable fear in the general public or in specific communities, especially when illness and deaths are substantial. Mitigating fear and discrimination directed toward persons infected with, and affected by, infectious disease can be important in controlling transmission. Persons who are feared and stigmatized may delay seeking care and remain in the community undetected. Quelling fear-driven stigmatization and discrimination during the SARS outbreak required tailored intervention strategies carried out by the SARS Community Outreach Team. These activities complemented traditional risk communication for the general public. To be effective, behavioural intervention approaches, messages, and materials had to be salient for the affected population, in this case Asian-American communities within the United States. Further, these interventions aimed at promoting an accurate understanding of the epidemic both in the general population and within the affected community, that is, the dynamic nature of the outbreak and its cause, treatment options, and prevention strategies. Through interpersonal connections, the team members worked to promote reassurance and enhance community resiliency.

### STIGMA and INFLUENZA (bird flu/H5N1, Swine flu/H1N1)

Barrett et al, 2008

The authors examined the role of stigma in social and institutional responses to infectious disease emergencies, to better understand and minimize these dynamics in the event of a pandemic of virulent influenza. By extension of these lessons to infectious diseases in general, stigma can be seen as a biosocial phenomenon with 4 essential elements. First, stigma can present major barriers against health care seeking, thereby reducing early detection and treatment and furthering the spread of disease. Second, social marginalization often can lead to poverty and neglect, thereby increasing the susceptibility of populations to the entry and amplification of infectious diseases. Third, potentially stigmatized populations may distrust health authorities and resist cooperation during a public health emergency. Finally, social stigma may distort public perceptions of risk, resulting in mass panic among citizens and the disproportionate allocation of health care resources by politicians and health professionals. The historical models discussed in this article illustrate the ways in which stigma and fear can severely impede efforts to manage the spread of an outbreak of virulent influenza. Yet, they also provide lessons for the mitigation or prevention of these social dynamics. Chief among these lessons is the importance of building a surge capacity for public trust.

Earnshaw et al, 2013

The authors examined the extent to which H1N1 was stigmatized at the height of the 2009 H1N1 pandemic in the U.S. and explores the role that H1N1 stigma played in people’s desire for physical distance from others with H1N1. H1N1 was the most stigmatized disease, with participants endorsing greater prejudice towards people with H1N1 than people with cancer or HIV/AIDS. Further, H1N1 stigma partially mediated the relationship between participants’ perceptions that H1N1 was threatening and their desire for physical distance from people with H1N1. Therefore, H1N1 stigma played a role in, but was not entirely responsible for, the relationship between perceptions that H1N1 was threatening and desire for distance from others with H1N1. Stigma is a complex paradox in relation to public health strategies during influenza pandemics – can both bolster public health efforts to protect people from influenza infection by increasing people’s desire for physical distance from others with influenza, but it may also undermine detection, treatment, and containment efforts thereby contributing to poor health outcomes of people infected with influenza and increasing the spread of influenza. It may be possible to reduce people’s feelings of prejudice towards others with influenza while maintaining their desire for distance from others with influenza. The challenge for social psychologists is to develop effective strategies to reduce influenza stigma without also reducing desire for distance from others with influenza. Effective stigma reduction strategies have been developed for other types of stigmas that could be adapted to reduce influenza stigma. However, many of these strategies rely on increasing contact with stigmatized individuals and are therefore ill-suited to reducing influenza stigma. Facing another paradox of how to reduce influenza stigma without increasing contact with people with influenza, social psychologists might consider exploring stigma-reduction strategies involving increasing community trust of government officials and health care providers. People who trust that their government officials and health care providers will protect them from the threat of influenza may be less likely to stigmatize others with influenza.

### STIGMA and TUBERCULOSIS

Chang et al, 2014

Setting: Tuberculosis (TB) related stigma is associated with lack of treatment adherence. Individual perceptions of stigma differ by societal context. Limited data are available on variations of TB stigma worldwide.

Objective: To describe the influence of TB stigma on knowledge, attitudes and responses to TB and to identify similarities and differences across countries.

Design: Systematic review of international descriptive studies.

Results: A total of 1268 studies were identified from PubMed/Medline, Web of Science, Cochrane, PsycINFO and Cumulative Index to Nursing and Allied Health Literature database searches. Eighty-three studies from 35 countries met the inclusion criteria for English, peer-reviewed, original and non-interventional studies. Variation and similarities in the influence of TB stigma on knowledge, attitudes and responses to TB across countries were identified. Stigma antecedents included negative attitudes and misperceptions regarding the causes of TB and the association with the human immunodeficiency virus. Decisions about illness disclosure and choices between traditional healers and public or private providers were influenced by TB stigma. Sex-influenced perceptions and management of TB and public health responses contributed to TB stigma.

Conclusion: Our findings confirm cultural variations with respect to TB and the potential for stigma. Cultural variations should be considered in the development of interventions aimed at reducing stigma and improving treatment adherence.

### STIGMA and EBOLA

Davtyan et al, 2014

The authors discussed how lessons learned from HIV infection could be useful for Ebola Virus Disease (EVD). Stigma in the context of EVD is disconcerting as it also originates from structural inadequacies, including poverty, lack of education, and political conflict. These factors combined with cultural practices subsequently influence attitudes, beliefs, and behaviors with respect to disease transmission Stigmatizing attitudes and behaviors directed towards those with EVD are similar to HIV/AIDS in many important ways. For instance, the idea that EVD only affects certain groups such as poor Africans and African immigrants is comparable to HIV/AIDS as it was attributed to homosexuality in the early days of the epidemic. Additionally, EVD and HIV/AIDS have both been categorized as divine retribution for committing undesirable acts. Other similarities include irrational and unfounded fear of contracting the pathogen through mechanisms that have not been scientifically indicated, stigmatization of people who associate with those living with the diseases in question and the perception that both diseases are the result of propaganda for population control. To address stigma in the context of EVD, we can draw upon lessons learned from HIV, consider and perhaps apply strategies that have shown promise. To disseminate accurate information to communities impacted by EVD in ways that facilitate translation of information into action, recruitment and training of Popular Opinion Leaders (POL), may be beneficial. POL include members of a community who are highly respected and who may have the ability to mobilize people to work for a common goal, in this case to fight stigma. Multi-level community interventions that include accurate information about disease transmission, skill-building, counselling and support, and testimonials from persons who survived the disease as well as those who took care of EVD patients may also be effective. Collective strategies such as social activism, capacity, resilience and skill building, and empathy-based contact promoting programs have been beneficial in the fight against HIV/AIDS-related stigma and can be tailored for addressing EVD-related stigma. In the long run, education, prevention, and a therapeutic vaccine will be the optimal solutions for reducing the stigma associated with both EVD and HIV.

Mayrhuber et al., 2017

Background: The Ebola virus disease epidemic between 2013 and 2016 in West Africa was unprecedented. It resulted in approximately 28.000 cases and 10.000 Ebola survivors. Many survivors face social, economic and health-related predicaments and media reporting is crucially important in infectious disease outbreaks. However, there is little research on reporting of the social situation of Ebola survivors in Liberia. Methods: The study used a mixed methods approach and analysed media reports from the Liberian Daily Observer (DOL), a daily newspaper available online in English. We were interested to know how the situation of Ebola survivors was portrayed; in what way issues such as stigma and discrimination were addressed; and which stigma reduction interventions were covered and how. We included all articles on the situation of Ebola survivors in the quantitative and in-depth qualitative analysis published between April 2014 and March 2016. Results: The DOL published 148 articles that portrayed the social situation of Ebola survivors between the 24 months observation period. In these articles, Ebola survivors were often defined beyond biological terms, reflecting on a broader social definition of survivorship. Survivorship was associated with challenges such as suffering from after-effects, social and economic consequences and psychological distress. Almost 50% of the articles explicitly mentioned stigmatisation in their reporting on Ebola survivors. This was contextualised in untrustworthiness towards international responses and the local health care system and inconclusive knowledge on cures and transmission routes. In the majority of DOL articles stigma reduction and engaging survivors in the response was reported as crucially important. Discussion: Reporting in the DOL was educational-didactical and well-balanced in terms of disseminating available medical knowledge and reflecting the social situation of Ebola survivors. While the articles contextualised factors contributing to stigmatisation throughout the reporting, journalistic scrutiny regarding effectiveness of interventions by government and NGOs was missing.

IASC, 2015

EVD outbreaks lead to significant mental and psychosocial effects in a number of ways, including social problems may emerge after a population is exposed to the virus and the EVD response: for example, breakdown of community support systems, and social stigma and discrimination associated with EVD. The mental health of specific groups requires special consideration, including those who have recovered from the disease and those who are living with the consequences of the epidemic, such as orphans and other family members, as well as health care staff and other frontline support workers. Support offered to such groups should be targeted and integrated into community-wide interventions to avoid reinforcing stigma.

Stigma and discrimination

Social stigma and discrimination can be associated with EVD, including towards persons who have been infected, their family members and health care and other frontline workers (1, 11, 12, 13). Steps must be taken to address stigma and discrimination at all phases of an EVD emergency response. Care should be taken to promote the integration of people who have been affected by EVD without over-targeting (i.e. without increasing stigma by drawing attention to them). Health and social services, for example, should, if at all possible, be available to all community members rather than just those affected by EVD. However, it should be noted that outreach can be essential, as some people who have survived EVD may isolate themselves due to prevailing stigma. Community members and leaders (including traditional healers and religious leaders as well as mental health service users and associations of people who have survived EVD) should be engaged to understand the sources of stigma and the steps that can be taken to dispel any unnecessary fears and misconceptions. At the same time, it should be recognized that empowerment of those who have survived and are affected by EVD is essential to overcoming stigma, including self- stigma, and discrimination.

Communication of the disease should involve providing community members with clear explanations and facilitate discussions regarding EVD as a disease, the introduction of interventions and services (e.g. ambulances, ETUs, contact tracing). Convey the process for isolation and treatment to communities, including what to expect when a person with EVD is picked up for admission to a facility or upon admission to a facility for treatment. Helping communities understand that it is possible to survive EVD is important in reducing stigma and discrimination against people who have survived EVD in the long term.

The needs of persons who have survived EVD

While some persons who survived EVD were welcomed home from ETUs, many more experienced stigma, discrimination and rejection by their communities. Some individuals were deserted by their spouses and other family members. Stigmatization was faced not only by individuals but by families and whole communities, resulting in people losing their jobs and even their homes. Similar experiences have been reported in previous outbreaks of Ebola and related diseases. Children, whether they have recovered from EVD themselves or have been cleared after a family member contracted the disease, can also experience significant disruption to their social supports, with changes to friendship/peer supports, social isolation and stigma and subsequent challenges in being able to re-engage in education. While it is important to address the specific needs of persons who have survived EVD in the emergency phase, in the long term it will be necessary to incorporate these groups into mainstream services. If services are seen as being equally distributed, the risk of continued stigma against persons who have survived EVD will decrease.

### STIGMA and LEPROSY

Topp and colleagues, 2019

The authors conducted a systematic review to synthesize what is known globally about effective interventions to reduce stigma associated with visible chronic skin diseases, including leprosy. Four electronic databases were searched until May 2018. Studies evaluating interventions to reduce stigmatization in patients with visible chronic skin diseases and applying at least one stigma-related outcome measure were included. Data were extracted on study design, country, study population, outcome measures and main findings. Results were subsequently synthesized in a narrative review. Critical Appraisal Skills Programme tools were used to assess study quality. Nineteen studies were included in the review. Study design was very heterogeneous and study quality rather poor. Thirteen studies addressed patients with leprosy in low-and middle-income countries, and one study each targeted patients with onychomycosis, leg ulcer, facial disfigurement, atopic dermatitis, vitiligo and alopecia. Evaluated interventions were mainly multi-faceted incorporating more than one type of intervention. While 10 studies focused on the reduction in self-stigma and 4 on the reduction in public stigma, another 5 studies aimed at reducing both. The present review revealed a lack of high-quality studies on effective approaches to reduce stigmatization of patients with visible chronic skin diseases. Development and evaluation of intervention formats to adequately address stigma is essential to promote patients’ health and well-being.

### STIGMA and HIV

Hartog et al, 2020

Stigmatisation and discrimination are common worldwide, and have profound negative impacts on health and quality of life. Research, albeit limited, has focused predominantly on adults. There is a paucity of literature about stigma reduction strategies concerning children and adolescents, with evidence especially sparse for low- and middle-income countries (LMIC). This systematic review synthesised child-focused stigma reduction strategies in LMIC, and compared these to adult-focused interventions. Relevant publications were systematically searched in July and August 2018 in the following databases; Cochrane, Embase, Global Health, HMIC, Medline, PsycINFO, PubMed and WorldWideScience.org, and through Google Custom Search. Included studies and identified reviews were cross-referenced. Three categories of search terms were used: (i) stigma, (ii) intervention, and (iii) LMIC settings. Data on study design, participants and intervention details including strategies and implementation factors were extracted. Within 61 unique publications describing 79 interventions, utilising 14 unique stigma reduction strategies, 14 papers discussed 21 interventions and 10 unique strategies involving children. Most studies targeted HIV/AIDS (50% for children, 38% for adults) or mental illness (14% vs 34%) stigma. Community education (47%), individual empowerment (15%) and social contact (12%) were most employed in child-focused interventions. Most interventions were implemented at one socio-ecological level; child-focused interventions mostly employed community-level strategies (88%). Intervention duration was mostly short; between half a day and a week. Printed or movie-based material was key to deliver child-focused interventions (37%), while professionals most commonly implemented adult-focused interventions (53%). Ten unique, child-focused strategies were all evaluated positively, using a diverse set of scales. Children and adolescents are under-represented in stigma reduction in LMIC. More stigma reduction interventions in LMIC, addressing a wider variety of stigmas, with children as direct and indirect target group, are needed.

Mak et al, 2017

Objective: The present study conducted a meta-analysis and systematic review on studies evaluating the effectiveness of stigma reduction programs in improving knowledge and reducing negative attitudes towards people living with HIV (PLHIV).

Results: Meta-analysis (k = 42 studies) found significant and small effect sizes in the improvement of the participants' knowledge of HIV/AIDS from interventions with (Cohen's d = 0.48, 95% CI [0.30, 0.66]) and without control groups (Cohen's d = 0.42, 95% CI [0.28, 0.57]). Significant and small effect sizes were found in the improvement of the participants' attitudes toward PLHIV from interventions with (Cohen's d = 0.39, 95% CI [0.23, 0.55]) and without control groups (Cohen's d = 0.25, 95% CI [0.11, 0.39]). Significant and small effect sizes were sustained at the follow-up assessments. Subgroup analysis showed that number of intervention sessions, intervention settings, and sample type significantly moderated the effect sizes in the meta-analysis. Findings from the systematic review of 35 studies indicated that most of the included studies showed positive results in reducing negative attitudes toward PLHIV and improving HIV-related knowledge. Most of the included studies tended to have low methodological quality.

Conclusion: The present meta-analysis and systematic review indicated that the studies generally found small improvement in HIV-related knowledge and reduction in negative attitudes towards PLHIV among the stigma reduction programs being evaluated. High-quality stigma reduction programs with multidimensional stigma indicators and psychometrically sound outcome measures are highly warranted.

### STIGMA and MENTAL ILLNESS

Janoušková et al., 2017

Video is considered to be an effective, easy to use tool employed in anti-stigma interventions among young people. Mass media has been shown to be effective for reducing stigma; however, there is insufficient evidence to determine the destigmatization effects of videos specifically. This article systematically reviews the effectiveness of video intervention in reducing stigma among young people between 13 and 25 years. We searched 13 electronic databases including randomized controlled trials, cluster randomized controlled trials, and controlled before and after studies. Of the 1426 abstracts identified, 23 studies (reported in 22 papers) met the inclusion criteria. Video interventions led to improvements in stigmatising attitudes. Video was found to be more effective than other interventions, such as classical face-to-face educational sessions or simulation of hallucinations. According to results of two studies, social contact delivered via video achieved similar destigmatization effect to that delivered via a live intervention. Although the quality of studies as well as the form of video interventions varied, the findings suggest that video is a promising destigmatization tool among young people; however, more studies in this area are needed. There was a lack of evidence for interventions outside of school environments, in low- and middle-income countries, and studies, which looked at long-term outcomes or measured impact on actual behaviour and implicit attitudes. The review generates recommendations for video interventions targeted at young people.

Nyblade et al, 2020

Stigma in health facilities undermines diagnosis, treatment, and successful health outcomes. Addressing stigma is fundamental to delivering quality healthcare and achieving optimal health. This correspondence article seeks to assess how developments over the past 5 years have contributed to the state of programmatic knowledge-both approaches and methods-regarding interventions to reduce stigma in health facilities, and explores the potential to concurrently address multiple health condition stigmas. It is supported by findings from a systematic review of published articles indexed in PubMed, Psychinfo and Web of Science, and in the United States Agency for International Development's Development Experience Clearinghouse, which was conducted in February 2018 and restricted to the past 5 years. Forty-two studies met inclusion criteria and provided insight on interventions to reduce HIV, mental illness, or substance abuse stigma. Multiple common approaches to address stigma in health facilities emerged, which were implemented in a variety of ways. The literature search identified key gaps including a dearth of stigma reduction interventions in health facilities that focus on tuberculosis, diabetes, leprosy, or cancer; target multiple cadres of staff or multiple ecological levels; leverage interactive technology; or address stigma experienced by health workers. Preliminary results from ongoing innovative responses to these gaps are also described. The current evidence base of stigma reduction in health facilities provides a solid foundation to develop and implement interventions. However, gaps exist and merit further work. Future investment in health facility stigma reduction should prioritize the involvement of clients living with the stigmatized condition or behavior and health workers living with stigmatized conditions and should address both individual and structural level stigma.

Thornicroft et al, 2016

Stigma and discrimination in relation to mental illnesses have been described as having worse consequences than the conditions themselves. Most medical literature in this area of research has been descriptive and has focused on attitudes towards people with mental illness rather than on interventions to reduce stigma. In this narrative Review, we summarise what is known globally from published systematic reviews and primary data on effective interventions intended to reduce mental-illness-related stigma or discrimination. The main findings emerging from this narrative overview are that: (1) at the population level there is a fairly consistent pattern of short-term benefits for positive attitude change, and some lesser evidence for knowledge improvement; (2) for people with mental illness, some group-level anti-stigma inventions show promise and merit further assessment; (3) for specific target groups, such as students, social-contact-based interventions usually achieve short-term (but less clearly long-term) attitudinal improvements, and less often produce knowledge gains; (4) this is a heterogeneous field of study with few strong study designs with large sample sizes; (5) research from low-income and middle-income countries is conspicuous by its relative absence; (6) caution needs to be exercised in not overgeneralising lessons from one target group to another; (7) there is a clear need for studies with longer-term follow-up to assess whether initial gains are sustained or attenuated, and whether booster doses of the intervention are needed to maintain progress; (8) few studies in any part of the world have focused on either the service user’s perspective of stigma and discrimination or on the behaviour domain of behavioural change, either by people with or without mental illness in the complex processes of stigmatisation. We found that social contact is the most effective type of intervention to improve stigma-related knowledge and attitudes in the short term. However, the evidence for longer-term benefit of such social contact to reduce stigma is weak. In view of the magnitude of challenges that result from mental health stigma and discrimination, a concerted effort is needed to fund methodologically strong research that will provide robust evidence to support decisions on investment in interventions to reduce stigma.

### STIGMA and MIXED CONDITIONS

Fisher et al, 2019

The authors identified some strategies to reduce disease-related stigma during infectious disease outbreaks and public health emergencies responses.

Brief, practical strategies to reduce disease-related stigma during a public health emergency may facilitate more effective control of emerging infectious diseases, as outlined below.

1. Anticipate Stigma and Be on the Watch for It: Stigma is common and has been documented in outbreaks of Ebola and other infectious diseases such as TB and SARS. Anticipating disease-related stigma during infectious disease outbreaks enables those planning and coordinating emergency response efforts to address it, and thereby potentially counteract its negative effects.

2. Assess What People Know and Don’t Know About the Disease to Help Identify Stigmatization: Misinformation and myths are key contributors to stigma. At the outset of a response, it is vital to find out what people affected or potentially affected by an epidemic know and don’t know, what they believe and don’t believe, what they are hearing and not hearing, and what they are saying and not saying about the disease. Rapid, low-resource means of assessment could include obtaining input from partner organizations and having discussions with community leaders and clinicians as they have important roles early in an emergency response. Other approaches are to analyze news media messaging, examine e-mails and phone calls to public health departments, and obtain information about which pages are being read the most on a public health department website. Responders can also conduct searches of publicly available social media posts and messages, although this may require significant expertise and staff time. These approaches would provide information about where people might lack knowledge, which, in turn could help determine where stigma might arise. Additionally, telephone polling, community meetings, and focus groups could provide direct information about barriers, opportunities, and potentially conflicting messages from other sources. Keeping open lines of communication between public health officials and the public is critical in any public health emergency. Whatever the method and focus of assessing current knowledge and beliefs, a systematic approach is needed to gather a valid and reliable basis of information from which to move forward.

3. Partner with Community Leaders and Groups to Address Stigma: Respected community groups and leaders can provide valuable insight about stigmatization and stigmatized groups, add legitimacy to public health efforts, and serve as a critical conduit to disseminate information to individuals and groups who might mistrust the government or other sources. As de facto cultural translators, community leaders can help determine which methods and messages may reduce stigma and help organize community events designed to reduce stigma.

4. Develop and Conduct Public Messaging and Community Campaigns to Fight Stigma: Developing informative public messages that do not convey or perpetuate stigma, and directly counter stigmatization of groups or individuals, is a critical step in addressing disease-related stigma. Disease responders can develop and disseminate messages through public outreach and social-marketing campaigns with trusted community leaders and subject matter experts. Working with community leaders or peer role models for messaging also can be helpful. It is important to tailor health messages and interventions for specific populations because the experience of disease-related stigma can vary across groups. Health-care workers are another important target group, though often overlooked, for messaging to decrease disease-related stigma. Studies indicate that messaging and education aimed at stigma among public health and health-care workers can have an impact on their attitudes.

5. Implement Practical Interventions, Skills-Building Training, and Other Educational Programs to Reduce Stigma: Information campaigns and public outreach by trusted community leaders and experts are necessary but may not be sufficient alone to counter stigma. In certain situations, education that is more intensive may be needed. Preferably, interventions should be relatively brief, as time is critical during an emerging disease response. They might be integrated into counseling staff and volunteers about fears. Creating opportunities for affected groups to interact with unaffected groups has been shown to be a promising approach to reduce stigma, particularly when combined with information-based interventions. Examples of contact-based interventions include engaging in 1-on-1 conversation or listening to a testimonial from an affected individual. Interventions that use skills building can focus on role-play exercises, strategies for resolving negative attitudes, and coping behavior such as engaging with and providing social support for stigmatized groups and refusing to exclude people who pose no risk of transmitting illness.

6. Evaluate Stigma-Reducing Efforts: Interventions designed to reduce stigma during public health emergency response efforts should be evaluated to assure that they are effective and to improve their effectiveness. Detailed evaluation methods are out of scope here, but we provide a few key points that would be relevant for an evaluation embedded within an emergency response. From the outset, members of the evaluation team should have a clear understanding of the program goals and evaluation objectives related to the stigma-reducing efforts being assessed. Evaluators should obtain feedback from those who delivered and from those who received stigma-reducing interventions to assess not only whether the interventions worked, but also whether they reached the target groups. Further, public health agencies can monitor public reactions to information to identify barriers, provide evidence of success, and determine a need for additional resources, and adjust messages accordingly.

Mak et al, 2006

The authors conducted a review on stigma in three different conditions: HIV/AIDS, TB and SARS. Results suggested that individuals with HIV/AIDS, TB, or those who were previously infected with SARS were stigmatized by the general public in a varying extent, with individuals diagnosed with HIV/AIDS being the most harshly and blatantly stigmatized, followed by those with TB and those who recovered from SARS. The public also perceived the infection of HIV/AIDS to be more internally controlled by the individuals and regarded individuals having HIV/AIDS as more responsible and blameworthy of their disease than their SARS and TB counterparts. Differences in disease attributions and stigma hinge on several features related to the diseases: (1) whether the disease creates physical limitations to the infected, (2) whether the disease poses serious consequences to others, and (3) whether the disease is associated with symbolic meaning or negative images. Although SARS and TB are associated with physical suffering, they are considered to be caused mainly by external factors, such as bacterial infection and poor hygiene, which are not perceived as morally reprehensible (Kelly, 1999). Thus, stigmatization of SARS and TB may be driven mainly by the dread of the illness itself, which may be reduced with a decrease level of perceived threat. Given that SARS is an acute and nascent disease with its nature still uncertain, the public may be less likely to blame people for their infection when much is unknown about the disease. The present study has identified constructs in the attribution process that can be targeted in stigma reduction programs. It also showed stigma to be related to public attitudes towards government policies. Whereas SARS stigma was found to affect public’s views towards prevention, public education, research, and protection of the infected, stigma did not seem to affect public attitudes towards AIDS research and legal protection of individuals with TB. In public health campaigns, to reduce stigma towards infectious diseases, emphasis should go beyond factual knowledge. More efforts should be placed in strategically changing the attributions made by the public towards infectious diseases.


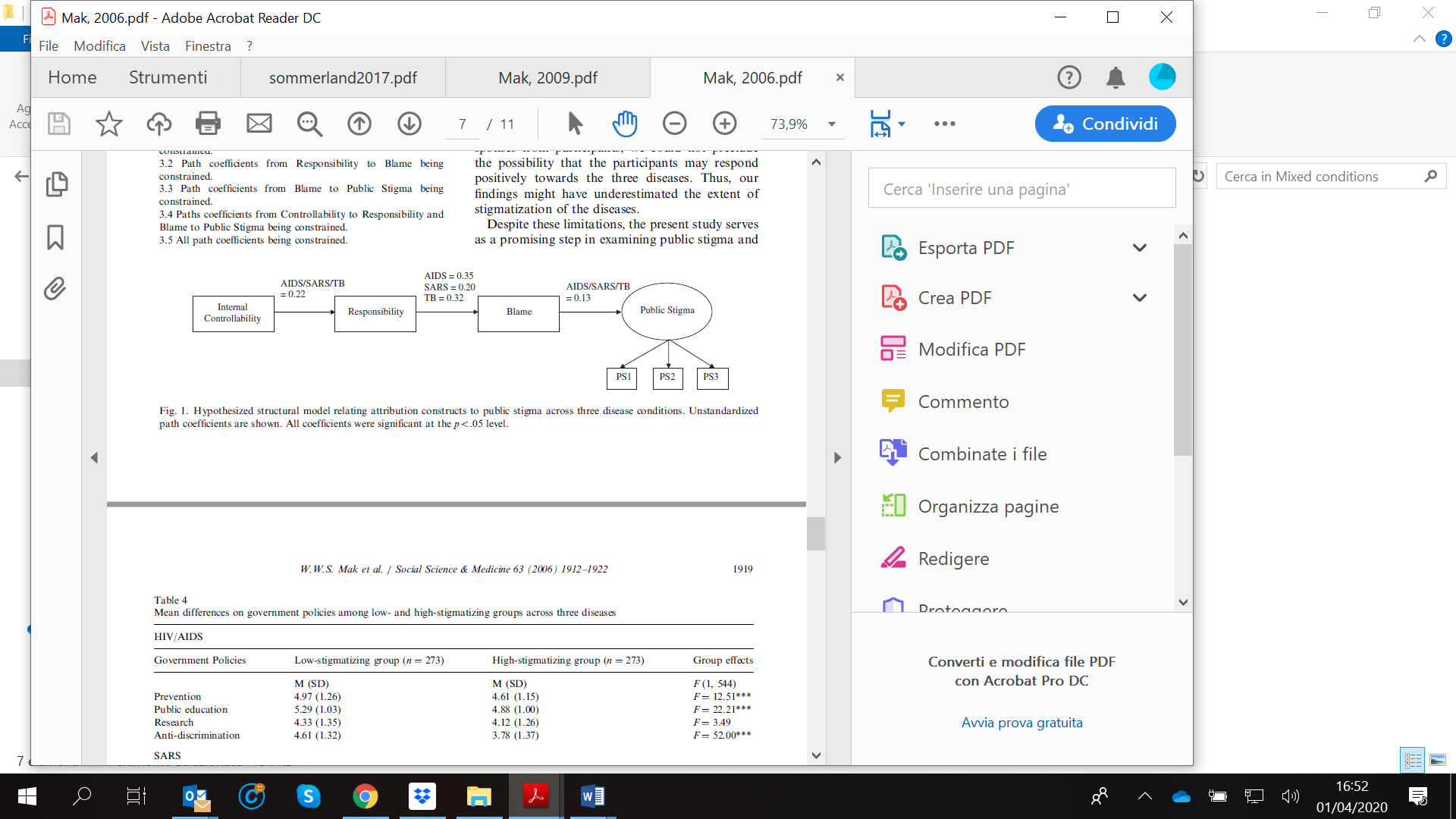


Mak et al, 2009

Mak and colleagues confirmed in a following paper on stigma associated with SARS, HIV/AIDS and TB, that stigma reduction and promotion of public awareness should focus not only on knowledge but also cognitive representations of illness and interpersonal contact to alleviate stigma. Along with providing psycho-education and information about treatment and medication, family-based interventions should focus on the self-stigma imposed on patients and caregivers.

Rao et al, 2019

The authors conducted a systematic review of original research on multi-level stigma-reduction interventions. They used the following eligibility criteria for inclusion: (1) peer-reviewed, (2) contained original research, (3) published prior to initiation of search on November 30, 2017, (4) evaluated interventions that operated on more than one level, and (5) examined stigma as an outcome. Twenty-four articles met the inclusion criteria. The articles included a range of countries (low, middle, and high income), stigmatized conditions/populations (e.g., HIV, mental health, leprosy), intervention targets (e.g., people living with a stigmatized condition, health care workers, family, and community members), and stigma reduction strategies (e.g., contact, social marketing, counselling, faith, problem solving), with most using education-based approaches. A total of 12 (50%) articles examined community-level interventions alongside interpersonal and/or intrapersonal levels, but only 1 (4%) combined a structural-level intervention with another level. Of the 24 studies, only 6 (25%) were randomized controlled trials. While most studies (17 of 24) reported statistically significant declines in at least one measure of stigma, fewer than half reported measures of practical significance (i.e., effect size); those that were reported varied widely in magnitude and were typically in the small-to-moderate range.

WHO, 2019

Over the past two decades, there has been a major increase in research into the effects of the arts on health and well-being, alongside developments in practice and policy activities in different countries across the WHO European Region and further afield. This report synthesizes the global evidence on the role of the arts in improving health and well-being, with a specific focus on the WHO European Region. Results from over 3000 studies identified a major role for the arts in the prevention of ill health, promotion of health, and management and treatment of illness across the lifespan. The reviewed evidence included study designs such as uncontrolled pilot studies, case studies, small-scale cross-sectional surveys, nationally representative longitudinal cohort studies, community-wide ethnographies and randomized controlled trials from diverse disciplines. The beneficial impact of the arts could be furthered through acknowledging and acting on the growing evidence base; promoting arts engagement at the individual, local and national levels; and supporting cross-sectoral collaboration. Particularly, the arts have been used to reduce stigma associated with certain health conditions. Arts programmes in schools have been used to improve mental health literacy, empathy and inclusion. Arts festivals have been found to increase positive attitudes towards mental health, increase appreciation of the abilities and creativity of people with mental illness and increase perceived collective efficacy within communities to improve mental health care. Drama has been used to address mental health stigma, with theatre productions on bipolar disorder found to reduce stigma in the short term among health-care providers. The Rural Art Roadshow initiative has been used to build community resilience, reduce stigma and promote a positive image of mental health in remote communities. In psychiatric inpatient units, song-writing has been found to reduce levels of experienced stigma, self-stigma and total stigma. Patterns in national press articles suggest a growing use of the arts to destigmatize mental illness, including challenging the belief that individuals with mental illness are incapable of work, thus highlighting the role of the arts in facilitating return to employment and breaking down barriers between those with and without mental illness . Storylines in major television sitcoms have been used to help to reduce the secrecy and shame surrounding postpartum psychosis. In particular, feelings of relatedness towards protagonists in such storylines were found to be a key factor in their success as an intervention. Physical health issues such as living with HIV or dementia can also be tackled effectively by the arts. Photo stories, creative activities and fiction writing have been found to disrupt stereotypes about HIV and to provide cathartic opportunities and increase social support among people with HIV. They also have an impact on health professionals by humanizing people with HIV by increasing the professionals’ understanding and supporting empathetic emotional responses. Drama performances about dementia, poetry projects and community choirs for people with and without dementia have been found to increase the understanding of dementia, shift negative attitudes and reduce stigma. The arts have also been found to promote broader health equity: arts programmes and theatre projects have increased the understanding of the health and fertility needs of individuals who are lesbian, gay, bisexual, transgender or queer (LGBTQ) or of another gender minority and have also increased empathy and self-reflection of personal biases . Films have also been shown to improve parental attitudes towards children who are LGBTQ.

### SUMMARY Table: additional materials

|  | **Title** | **Stigmatised condition** | **Type of additional material** | **Summary of relevant content** |
| --- | --- | --- | --- | --- |
| (American Psychological Association 2020) | Combating bias and stigma related to COVID-19 | COVID-19 | Technical guidance, briefing papers/reports | Epidemics and pandemics tend to provoke xenophobia and stigma. Bias, stigma and discrimination are public health threats. Stigma needs to be addressed and reduced through spreading the facts, engaging with social influencers, amplifying voices of lived experience, portraying a wide range of ethnic groups in information materials, promoting ethical journalism, and correcting myths, rumours and stereotypes. |
| (Asmundson & Taylor 2020) | Coronaphobia: Fear and the 2019-nCoV outbreak | COVID-19 | Editorial, commentary, opinion piece, correspondence, narrative report | Coronaphobia and coronavirus-related xenophobia can be fuelled by lack of information and misinformation, sensationalist media. Important to use evidence-based strategies to address these issues. |
| (Barrett & Brown 2008) | Stigma in the Time of Influenza: Social and Institutional Responses to Pandemic Emergencies | Influenza | Editorial, commentary, opinion piece, correspondence, narrative report | Focus on stigma in the event of a pandemic of virulent influenza. Building public trust is a key factor to mitigate or prevent the social dynamics of stigma and fear, that can impede efforts to manage a viral outbreak. |
| (Centres for Disease Control and Prevention 2020) | Coronavirus Disease 2019 (COVID-19): Reducing stigma | COVID-19 | Technical guidance, briefing papers/reports | Fear about COVID-19 can lead to stigma and discrimination, and stigma comes with multiple negative consequences. Stopping stigma is critically important, e.g. through sharing correct information. |
| (Chang & Cataldo 2014) | A systematic review of global cultural variations in knowledge, attitudes and health responses to tuberculosis stigma | Tuberculosis | Systematic review* | Stigma antecedents included negative attitudes and misconceptions; stigma influenced decisions of disclosure and help-seeking; sex-influenced perceptions and management of tuberculosis and public health responses contributed to stigma. Stigma-reduction interventions need to consider cultural variation. |
| (Davtyan *et al.* 2014) | Addressing Ebola-related stigma: Lessons learned from HIV/AIDS | Ebola | Editorial, commentary, opinion piece, correspondence, narrative report | Can address Ebola virus disease using lessons learned from HIV: disseminating accurate information, supporting translating of information to action, involving popular opinion leaders in local communities, using multi-level community interventions, testimonials from persons who have survived the disease/taken care of patients, collective strategies, empathy-based contact. In the long run, stigma can be reduced via education, prevention and availability of a vaccine. |
| (Devakumar *et al.* 2020) | Racism and discrimination in COVID-19 responses | COVID-19 | Editorial, commentary, opinion piece, correspondence, narrative report | COVID-19 has led to discriminatory responses, disproportionately affecting already marginalised groups. Programmes to counter discrimination and division should be designed using social media, with a focus on sharing accurate information to reduce stigma and fear. |
| (Earnshaw & Quinn 2013) | Influenza stigma during the 2009 H1N1 pandemic | Influenza | Data-based study reporting on stigma experiences | Stigma in relation influenza pandemics is complex – can protect people from illness by increasing desire for physical distance, but also contribute to poor health outcomes for people experiencing or anticipating stigma. Cannot rely on strategies based on increasing social contact, instead need to reduce stigma e.g. through increased community trust. |
| (Earnshaw 2020) | Don’t Let Fear of Covid-19 Turn into Stigma | COVID-19 | Editorial, commentary, opinion piece, correspondence, narrative report | Stigma exacerbates suffering from infectious disease epidemics. Strategies - e.g. education, openness, anti-discrimination policies, and providing social support – can reduce stigma and strengthen resilience to mitigate impacts of stigma exposure. |
| (Fischer *et al.* 2019) | Addressing Disease-Related Stigma during Infectious Disease Outbreaks | Mixed conditions | Editorial, commentary, opinion piece, correspondence, narrative report | Brief, practical strategies to reduce disease-related stigma in a public health emergency can facilitate more effective control of emerging infectious diseases. Necessary to anticipate disease-related stigma during infectious disease outbreaks and plan to address it. Need to understand what people (do not) know, believe, hear, and say. Need to engage with local leaders, service providers; analyse media etc. Open communication needed between health officials and the public. Need to partner with community organisations; develop and conduct public messaging and community campaigns; implement practical interventions, skills-building, and educational programmes. Create opportunities for affected groups to interact with non-affected groups. |
| (Hartog *et al.* 2020) | Stigma reduction interventions for children and adolescents in low- and middle-income countries: Systematic review of intervention strategies | HIV/AIDS | Systematic review* | Most studies focused on HIV/AIDS or mental illness. Most common intervention types included community education, individual empowerment, and social contact. Interventions generally implemented at only one socio-ecological level, generally at community-level. Interventions were generally brief (0.5 day – 1 week); delivered using printed or movie-based materials (for children) or via professionals (for adults). Child-focused interventions all evaluated positively. More evidence needed from LMICs, addressing a wider variety of stigmas. |
| (IASC Reference Group on Mental Health and Psychosocial Support 2015) | Mental Health and Psychosocial Support in Ebola Virus Disease Outbreaks. A Guide for Public Health Programme Planners | Ebola | Technical guidance, briefing papers/reports | To avoid reinforcing stigma, support offered to groups affected by Ebola virus disease should be targeted and integrated into community-wide interventions, particularly after the immediate emergency phase. Necessary to address stigma and discrimination at all phases of an Ebola virus disease emergency response; integration of people affected without over-targeting. Some services available to all and not only those affected by virus, but also outreach strategies to people who are isolated due to stigma. Involving community members and leaders to understand source of stigma and specific fears and misconceptions. Empowerment of survivors and those affected by the virus is essential to overcoming stigma. Clear communication about disease, interventions, and that it is possible to survive the disease. |
| (IASC Inter-Agency Standing Committee 2020) | Interim briefing note: Addressing mental health and psychosocial aspects of COVID-19 Outbreak | COVID-19 | Technical guidance, briefing papers/reports | Epidemics cause stress, worries, and fears, that can lead to stigmatising reactions and social exclusion. Importance of promoting integration, appropriate risk communication, recognising key groups potentially facing stigma. Outlines globally recommended activities to address stigma. |
| (Janoušková *et al.* 2017) | Can video interventions be used to effectively destigmatize mental illness among young people? A systematic review | Mental illness | Systematic review* | Video-based interventions led to improvements in stigmatising attitudes; was more effective than face-to-face educational sessions or simulation of hallucinations. Social contact via video achieved similar impact to live social contact. Methodological quality of studies varied, but findings indicate video is a promising tool for stigma-reduction amongst young people. Lack of evidence from outside schools, LMICs, long-term outcomes, impact on behaviour and implicit attitudes. |
| (Lin 2020) | Social Reaction toward the 2019 Novel Coronavirus (COVID‑19) | COVID-19 | Editorial, commentary, opinion piece, correspondence, narrative report | Anti-stigma programmes are needed to break the misinformation of COVID-19, increasing the public’s knowledge of the virus, and positive and supporting messaging. |
| (Logie & Turan 2020) | How Do We Balance Tensions Between COVID-19 Public Health Responses and Stigma Mitigation? Learning from HIV Research. | COVID-19 | Editorial, commentary, opinion piece, correspondence, narrative report | Fear of COVID-19 can be addressed through correcting misinformation. Public health response to COVID-19 can unwittingly exacerbate stigma; attention on physical distancing, travel restrictions, misinformation, and engaging with affected communities. |
| (Mak *et al.* 2006) | Comparative stigma of HIV/AIDS, SARS, and Tuberculosis in Hong Kong | Mixed conditions | Data-based study reporting on stigma experiences | Stigmatisation often driven by dread of illness, which can be reduced by decreasing level of perceived threat. Need to consider how illness is attributed in public mind, so that this process can be targeted in stigma-reduction programmes. In public health campaigns it is necessary to go beyond factual knowledge to reduce stigma, need to strategically change attributions made by public. |
| (Mak *et al.* 2009) | A comparative study of the stigma associated with infectious diseases (SARS, AIDS, TB) | Mixed conditions | Data-based study reporting on stigma experiences | Stigma reduction and promotion of public awareness should not only focus on knowledge, but also cognitive representations of illness. Need to include interpersonal contact to alleviate stigma. Along with providing psycho-education and information about treatment and medication, family-based interventions should focus on the self-stigma imposed on patients and caregivers. |
| (Mak *et al.* 2017) | Meta-analysis and systematic review of studies on the effectiveness of HIV stigma reduction programs | HIV/AIDS | Systematic review* | Interventions led to small, significant improvements in negative attitudes, sustained at follow-up assessments. Effect sizes moderated by number of intervention sessions, intervention setting, and sample type. Most studies were of low methodological quality. More high-quality research needed, with multidimensional stigma indicators and psychometrically sound outcome measures. |
| (Mayrhuber *et al.* 2017) | "We are survivors and not a virus:" Content analysis of media reporting on Ebola survivors in Liberia | Ebola | Data-based study reporting on stigma experiences | In reporting on Ebola virus disease stigma, stigma reduction and engaging survivors in this response was highlighted as crucially important. Newspaper reporting itself was educational-didactical, balancing information on medical knowledge and reflecting on the social situation of Ebola survivors. |
| (Nature 2020) | End coronavirus stigma now | COVID-19 | Editorial, commentary, opinion piece, correspondence, narrative report | Coronavirus pandemic has fuelled racist reactions. To reduce stigma and discrimination it is important to avoiding language associating virus with specific countries/regions, or placing blame on specific groups. |
| (Nyblade *et al.* 2019) | Stigma in health facilities: Why it matters and how we can change it | Mental illness | Systematic review* | Interventions focused on HIV, metal illness and substance abuse stigma. Multiple common stigma-reduction approaches used, implemented in multiple ways. Health facility stigma reduction should prioritise involvement of people living with the stigmatised condition/behaviour, or health workers living with the stigmatised condition/behaviour. Need to address both individual and structural level stigma. |
| (Person *et al.* 2004) | Fear and Stigma: The Epidemic within the SARS Outbreak | SARS | Editorial, commentary, opinion piece, correspondence, narrative report | Outbreak associated with fear, stigmatisation and discrimination. Mitigating these impacts can be important for controlling transmission. Tailored intervention strategies with community outreach strategies were used to complement traditional risk communications, materials needed to be tailored for the affected population, and interventions should aim to promote an accurate understanding of the epidemic. Interpersonal connections can help to promote reassurance. |
| (Rao *et al.* 2019) | A systematic review of multi-level stigma interventions: State of the science and future directions | Mixed conditions | Systematic review* | Multiple intervention targets in focus, e.g. people living with a stigmatized condition, health care workers, family, and community members. Multiple stigma reduction strategies used, e.g., contact, social marketing, counselling, faith, problem solving. Most interventions conducted at community-level, some on interpersonal and/or intrapersonal level, only one considered structural-level. Generally reported improvements in at least one measure of stigma, but practical significance of this not often assessed. |
| (Thornicroft *et al.* 2016) | Evidence for effective interventions to reduce mental-health-related stigma and discrimination | Mental illness | Editorial, commentary, opinion piece, correspondence, narrative report | 1) At population level consistent pattern of short-term benefits for positive attitudes change, to some extent also knowledge; 2) group-level interventions targeting people with mental illness show promise; 3) interventions based on social contact, with specific target groups, achieve at least short-term improvement in attitudes, to some extent also knowledge; 4) studies heterogeneous, rarely strong study designs or large samples; 5) lack of evidence from LMICs; 6) cannot overgeneralise findings from one target group to another; 7) need more longer-term follow-ups; 8) need more focus on service users’ perspectives, focus on behaviours/discrimination. |
| (Topp *et al.* 2019) | Strategies to reduce stigma related to visible chronic skin diseases: a systematic review | Leprosy | Systematic review* | Research in field has used varied study designs. Interventions in the field mainly multi-faceted, incorporating multiple types of interventions. Targeted self-stigma, public-stigma, or both. Methodological quality of studies quite poor, lack of high-quality research on affective approaches to reducing stigma in this area. |
| (World Health Organization 2019) | What is the evidence on the role of the arts in improving health and well-being? A scoping review. | Mixed conditions | Scoping review | Arts recognised to have a major role in health promotion. Can reduce stigma associated with health conditions, and improve mental health literacy, empathy, inclusion, social support, reduce secrecy and shame. E.g. arts festivals, drama, and storytelling can reduce stigma, increase positive attitudes and appreciation of people with mental illness, challenge inaccurate beliefs and build community resilience. |
| (World Health Organization 2020a) | Mental Health and Psychosocial Considerations During COVID-19 Outbreak | COVID-19 | Technical guidance, briefing papers/reports | Messages to support mental health and psychosocial wellbeing of different target groups during the coronavirus outbreak. |
| (World Health Organization 2020b) | Social stigma associated with COVID-19 | COVID-19 | Technical guidance, briefing papers/reports | Guidance to prevent and address social stigma associated with COVID-19, targeted at government, media and local organisations, including guidance on terminology, communication and messaging. |
| *Systematic review not selected as key article to provide evidence for this review, but was included as additional materials | | | | |

## Quality assessment: AMSTAR-2

Risk of bias will be assessed for studies providing evidence on the impact of anti-stigma interventions in relation to a given condition. The methodological quality of systematic reviews will be assessed using the AMSTAR-2 tool.

AMSTAR 2 domains: 1 PICO, 2 protocol, 3 study design, 4 search strategy, 5 study selection, 6 data extraction, 7 excluded studies, 8 included studies, 9 risk of bias, 10 funding sources, 11 meta-analysis, 12 impact risk of bias, 13 discussing risk of bias, 14 heterogeneity, 15 publication bias, 16 conflicts of interest.

| Systematic reviews | AMSTAR-2 Domain | | | | | | | | | | | | | | | | |
| --- | --- | --- | --- | --- | --- | --- | --- | --- | --- | --- | --- | --- | --- | --- | --- | --- | --- |
|  | 1 | 2 | 3 | 4 | 5 | 6 | 7 | 8 | 9 | 10 | 11 | 12 | 13 | 14 | 15 | 16 |  |
| Mills 2020 |  |  |  |  |  |  |  |  |  |  | n/a | n/a |  |  |  |  |  |
| Büchter 2017 |  |  |  |  |  |  |  |  |  |  |  |  |  |  |  |  |  |
| Heim 2018 |  |  |  |  |  |  |  |  |  |  | n/a | n/a |  |  |  |  |  |
| Mehta 2015 (q1) |  |  |  |  |  |  |  |  |  |  | n/a | n/a |  |  |  |  |  |
| Mehta 2015 (q2) |  |  |  |  |  |  |  |  |  |  | n/a | n/a |  |  |  |  |  |
| Hanisch 2016 |  |  |  |  |  |  |  |  |  |  | n/a | n/a |  |  |  |  |  |
| Clement 2013 |  |  |  |  |  |  |  |  |  |  |  |  |  |  |  |  |  |
| Andersson, 2019 |  |  |  |  |  |  |  |  |  |  | n/a | n/a |  |  |  |  |  |
| Ma, 2019 |  |  |  |  |  |  |  |  |  |  | n/a | n/a |  |  |  |  |  |
| Pantelic 2019 |  |  |  |  |  |  |  |  |  |  | n/a | n/a |  |  |  |  |  |
| Feyissa, 2019 |  |  |  |  |  |  |  |  |  |  | n/a | n/a |  |  |  |  |  |
| Sermrittirong, 2014 |  |  |  |  |  |  |  |  |  |  | n/a | n/a |  |  |  |  |  |
| Sommerland, 2017 |  |  |  |  |  |  |  |  |  |  | n/a | n/a |  |  |  |  |  |

|  | Criterion met |
| --- | --- |
|  | Criterion not met |
|  | Criterion partially met |
| n/a | Not applicable |

# PART 2: FROM EVIDENCE TO RECOMMENDATIONS

## Evidence to Decision Table

|  | **Judgement** | **evidence** |
| --- | --- | --- |
| **Problem** | Is the problem a priority?  ○ No ○ Probably no ○ Probably yes **x Yes**  ○ Varies ○ Don't know | - The outbreak of COVID-19 has been extremely stressful for individuals, families, communities and nations. Given that COVID-19 is a new disease, it is understandable that its emergence and spread has caused confusion, anxiety and fear among the general public. Fear and anxiety about COVID-19 can result in stigma and discrimination toward people, places, or things associated with the disease (WHO,2020; APA 2020). - The 2020 COVID-19 outbreak has provoked stigmatisation and discriminatory behaviours against people of certain ethnic backgrounds as well as anyone perceived to have been in contact with the virus. Anecdotal evidence and media reporting outline experiences of stigma and discrimination amongst people affected by or associated with COVID-19 (Devakumar et al, 2020; WHO, 2020). - Discriminatory attitudes and behaviours are also evident in the public “shaming” of people who are deemed to not adhere appropriately to COVID-19 related public health measures, such as social distancing and quarantining indoors - For years, it was common for viral diseases to be associated with the landscapes, places or regions where the first outbreaks occurred. But in 2015, the WHO introduced guidelines to stop this practice and thereby reduce stigma and negative impacts such as fear or anger directed towards those regions or their people. The guidelines underlined the point that viruses infect all humans: when an outbreak happens, everyone is at risk, regardless of who they are or where they are from. Continuing to associate a virus and the disease it causes with a specific place is irresponsible and needs to stop (Nature, 2020) - These reports are in line with previous evidence indicating that stigma has been a key concern for many in relation to comparable viral outbreaks and epidemics, e.g. severe acute respiratory syndrome (SARS), middle east respiratory syndrome (MERS), and Ebola virus disease. - The impact of stigma can be widespread. In relation to COVID-19, concerns regarding stigma are relevant not only for people who have a current confirmed or suspected infectious condition. Rather stigma can also occur after a person has been released from COVID-19 quarantine or is no longer symptomatic, even though they are no longer a risk for spreading the virus to others. Furthermore, stigma can also occur via association, meaning stigma can be experienced also by people associated with COVID-19 due to their work (e.g. healthcare workers), country of origin or ethnicity (public perception of places and populations amongst whom the virus is more common), or affiliation with a person who is unwell (e.g. caregivers, family members) (CDC, 2020; Thornicroft et al, 2016; IASC 2020). - Fear and stigma toward the epidemics of COVID‑19 may lead to negative consequences of disease control (Chung-Ying Lin, 2020). - Differences in disease attributions and stigma hinge on several features related to the diseases: (1) whether the disease creates physical limitations to the infected, (2) whether the disease poses serious consequences to others, and (3) whether the disease is associated with symbolic meaning or negative images (Mak et al, 2006). - Addressing stigma is fundamental to delivering quality healthcare and achieving optimal health (Nyblade et al, 2020). - The role of popular media and communication is an important concern: misinformation (and information) is often aided by sensational popular media headlines and foci, which have been shown to fuel health-related fears and phobias and to contribute to stigma and discrimination which hamper response efforts (Asmundson 2020). - Public stigma can distort public perceptions of risk, resulting in mass panic. A key lesson from past models of stigma and fear is the importance of mitigating this by building a surge capacity for public trust (Barrett et al, 2008). - The perceived vulnerability to disease may play a role in stigma and also in “coronaphobia”, with people who misinterpret their bodily sensations as signs of potential infection with the 2019-nCoV coronavirus (Asmundson, 2020). - Providing recommendations to reduce stigma in relation to COVID-19 has recently been highlighted as a key priority by an editorial on coronavirus and stigma published in Nature, which concluded that “leaders want to listen and act on expert scientific advice”. Evidence-informed stigma-reduction strategies should therefore urgently be provided. |
| **Values** | Is there important uncertainty about or variability in how much people value the main outcomes?  ○ Important uncertainty or variability ○ Possibly important uncertainty or variability ○ Probably no important uncertainty or variability **x No important uncertainty or variability** | - Despite how the outbreak of the novel coronavirus is very recent, the focus on the risk of disease-related stigma was very strong immediately. In fact, many institutions were quick to publish documents in which this issue was addressed, as evidenced by the materials in the section above on “priority”. - For example, a recent article in the International Journal of Science Nature highlights the prejudiced attacks that have taken place against people of Asian descent around the world since the outbreak was first reported, with untold human costs. The article’s emphasis on the importance of stigma reduction is evident in its title, “End coronavirus stigma now “, and with regards to this it posits that many leaders want to listen to and act on expert scientific advice to deal with the impacts of this pandemic (Nature, 2020). - Stigmatisation is reported to have profound negative impacts on health and quality of life. At least in relation to mental illnesses, the prevailing stigma and discrimination have been described as having worse consequences than the conditions themselves (Thornicroft et al, 2016). - Healthcare and social care workers hold a critical role in the management of disease outbreaks, but this group can also experience stigma and related stress due to their association with the illness. Stigma can thus reduce the capacity of these key frontline workers to perform professionally, and protecting their mental wellbeing through strategies addressing stigma is important (IASC, 2020) - Overall, although much direct evidence is lacking, there is probably no important uncertainty about or variability in how much communities recognise the importance of truth and justice, hence reject in principle negative behaviour towards people with a disease. There is therefore a common value in the reduction of stigma related to COVID-19 and to conditions sharing some similarities with COVID-19 |
| **Balance of effects** | Does the balance between desirable and undesirable effects favor the intervention or the comparison?  ○ Favors the comparison ○ Probably favors the comparison ○ Does not favor either the intervention or the comparison **x Probably favors the intervention** ○ Favors the intervention  ○ Varies ○ Don't know | - There is no direct evidence on the effectiveness of interventions with a stated aim of reducing stigma in people with COVID-19. - There is widespread evidence and consensus that mechanisms (drivers and facilitators) leading to stigma, its manifestations, impact and interventions that are effective to reduce stigma are very similar across conditions and cultures (Van Brakel, 2006; Van Brakel et al, 2019; Stangl et al, 2019); providing a rationale for considering evidence on stigma-reduction from comparable stigmatised conditions. - There is some evidence suggesting the efficacy of a variety of interventions with a stated aim of reducing stigma in conditions sharing some similarities with COVID-19, namely SARS, MERS, Influenza-bird flu/H5N1/avian flu/avian influenza, Swine flu/H1N1, Ebola, Tuberculosis, Leprosy, HIV/AIDS, mental illness. Indirectly, this evidence may inform the development of recommendations to reduce stigma related to COVID-19. Previous work on stigma reduction in relation emerging disease outbreaks has likewise drawn on lessons learned from anti-stigma strategies applied to other conditions. There is evidence of the substantial negative impacts of stigma and discrimination where the measures have not been taken to combat them. - In particular, we found evidence in support of stigma-reducing interventions for leprosy, tuberculosis, HIV/AIDS and mental illness. - The studies included in the identified systematic reviews have substantial methodological and clinical heterogeneity, and consequently formal meta-analyses have been conducted only in very few cases. - Most systematic reviews included primary studies with heterogeneous study designs, including randomised trials, quasi-experimental studies, and studies with mixed-methods designs. - When available, effect sizes of anti-stigma interventions (across conditions) are small-to-moderate. - Stigma-reduction often involves correcting myths, rumours and stereotypes, and challenging bias. The emphasis public health campaigns to reduce stigma towards infectious diseases, emphasis should go beyond factual knowledge. More efforts should be placed in strategically changing the attributions made by the public towards infectious diseases (Mak et al, 2006), e.g. stigma and fears around SARS and TB (“externally” caused, posing a potential consequence to others) may be “mainly driven by fear of the illness itself”, which may be reduced with a decrease level of perceived threat. Mitigating fear and discrimination directed toward people infected with, and affected by, infectious disease can be important in controlling transmission. - Interventions with attitude-changing or knowledge-shaping components, such as information and communication programmes, have been shown to be effective in reducing stigma for leprosy, tuberculosis (case-control and quasi experimental studies), HIV, mental illness. These intervention components have a focus on educating the general population, family members and/or people about the illness while also projecting positive images of people with that illness. For mental illness the interventions targeted to people in the workplace may be particularly effective in changing employees’ knowledge of mental disorders, as well as helping behaviour. - Involving key community stakeholders, community leaders, social influences, and people with lived experience of the stigmatised condition is recommended in developing and implementing effective strategies to address stigma - Training popular opinion leaders is effective in reducing avoidance intent and prejudicial attitude and improving compliance to universal precaution for HIV and TB. - Involvement of people affected by the condition was often found to be a key factor for success in a range of interventions in leprosy, HIV, tuberculosis and mental illness. Using champions both in local community meetings and in (mass) media has been successful in leprosy, HIV, tuberculosis, and mental health. - Integration of specific interventions into general health care programmes has been shown to be a successful implementation strategy for reducing stigma for leprosy and HIV. - Group-based interventions can reduce stigma as experienced by persons with stigmatised conditions: group-based behavioural interventions may be effective in reducing stigma as experienced by people with HIV (enacted, anticipated, internalised) (Andersson, 2019), and support groups showed promise in reducing Internalised and anticipated stigma as experienced by persons with TB (Sommerland, 2017) - Psycho-educational interventions (Ma, 2019) and social empowerment strategies (Pantellic 2019) can reduce self-stigma amongst people with HIV - Community-based strategies can also reduce experiences of stigma amongst persons with the stigmatised condition: community support initiatives reduced stigma (enacted, anticipated, internalised) amongst people with HIV (Andersson, 2019), and conscious efforts to improve community attitudes showed promise in reducing Internalised and anticipated stigma as experienced by persons with TB (Sommerland, 2017)Self-help interventions have been shown to be of benefit in reducing self-stigma in mental illness, HIV, leprosy and TB.Mass media interventions may reduce prejudice for mental illness in the immediate, short and medium term. - Arts programs or interventions using different forms of art have shown to be of benefit in reducing stigma associated with certain health conditions. Arts programmes in schools, arts festival and drama been found to increase positive attitudes towards mental health and increase perceived collective efficacy within communities to improve mental health care. - Interventions to reduce mental illness stigma in the workplace can be effective in improving knowledge and helping behaviours. - LMIC-based research is very limited, and available evidence often based on small-scale studies/research of mixed methodological quality/heterogeneous designs. - Studies investigating the long-term impact of interventions to reduce stigma are lacking. - In general, there is no evidence suggesting that these interventions may be associated with undesirable effects. - As a general rule, it has been suggested that public health strategies to improve access to COVID-19 testing and employment sick leave benefits may be very effective strategies to reduce stigma. - It has been suggested that movement restrictions, physical distancing, surveillance mechanisms, travel bans and quarantine should always include anti-stigma and anti-xenophobia public messaging, and training of legal authorities, aiming to avoid criminalising breaching COVID-19 related health policies. Approaches should focus on empowering and strengthening communities and support people to protect their own and others’ health. Strategies to avoid transmissions through avoiding in-person contact should be framed as “physical distancing”, rather than “social distancing” which can exacerbate otherness and stigma, or “social isolation” which can limit access to social support. Building connections via kindness and caring can motivate uptake of non-stigmatising physical distancing. - The language and messaging used in public health strategies is highlighted as one means of reducing stigma. In relation to COVID-19 specifically, the WHO Department of Mental Health and Substance Use developed a document in which there are a series of messages that can be used in communications to support mental and psychosocial well-being in different target groups during the outbreak in a way that reduced stigma and misinformation. - The importance of messaging in stigma reduction was also highlighted by a recent editorial in Nature (2020), which states “On terminology, the advice is clear: we must all do everything we can to avoid and reduce stigma; not associate COVID-19 with particular groups of people or places; and emphasize that viruses do not discriminate — we are all at risk.” - In language and messaging regarding COVID-19, it is important to consider how affected people are discussed in relation to illness (e.g. “people treated for COVID-19”, instead of “victims” or “cases”), whether countries, locations or ethnicity are unduly mentioned (e.g. “COVID-19”, not “Chinese Virus”), how sickness, treatment, recovery and death are described (e.g. “person contracted COVID-19”, not people “transmitting COVID-19” or “super-spreaders”; not “fighting/battling the disease” or “succumbing to illness” indicating role of personal character in illness trajectory), what tone is taken (e.g. reporting information based on official health data, rather than repeating unconfirmed rumours; emphasising effectiveness of prevention and treatment, instead using of hyperbolic negative language [“plague”, “apocalypse”] or dwelling on negatives or messages of threat). - Language in media in particular can be important, as media reporting shapes popular language and communication (WHO 2020). - Anti-stigma messaging should also be targeted in view of the specific beliefs and fears and drive stigma in a given setting, community, or target population. - To understand stigma in a given context, it might be required to scope information from local organisations, through discussions with community leaders, clinicians, analysing news messages, most accessed information on public health websites, social media posts etc. Contextualised insights regarding the particular beliefs and fears that drive stigma can provide information based on which anti-stigma efforts can be developed. |
| **Resources required** | How large are the resource requirements (costs)?  ○ Large costs ○ Moderate costs ○ Negligible costs and savings ○ Moderate savings ○ Large savings  **x Varies** ○ Don't know | - No direct evidence identified for this. - Most of the effective interventions described above require functioning health care systems and economic resources. - It is also often recommended that stigma-reduction forms a part of broader integrated health messaging (IASC 2020), indicating an expectation that there are resources to implement such a response on a population level. - A key principle of Disaster Risk Reduction is that investment in health systems in advance of epidemics and other emergencies is more efficient than efforts to build capacity rapidly during an emergency itself, and forms the third priority of the Sendai Framework on Disaster Risk Reduction (Aitsi-Selmi & Murray, 2015). - There is, however, evidence for substantial negative effects [and costs?] of poor health behaviours in the population linked to stigma. |
| **Equity** | What would be the impact on health equity?  ○ Reduced ○ Probably reduced ○ Probably no impact **x Probably increased** ○ Increased  ○ Varies ○ Don't know | - COVID-19 policy responses have disproportionately affected the populations at a global level. People who are over-represented in lower socioeconomic groups have limited health-care access, or work in precarious jobs. This is especially so in resource-poor settings that lack forms of social protection. Self-isolation is often not possible, leading to higher risk of viral spread. Ethnic minority groups are also at greater risk because of comorbidities. Furthermore, migrants, particularly those without documents, avoid hospitals for fear of identification and reporting, ultimately presenting late with potentially more advanced disease. For these reasons, marginalised groups (homeless people, people who are incarcerated, migrants and refugees, undocumented immigrants, and racial minorities) may be particularly exposed to stigma. Implementing stigma-related interventions, therefore, may likely reduce inequalities between marginalised groups and the society as a whole. By contrast, if stigma-related interventions may be implemented by economically developed contexts only, then their implementation may increase inequalities between high-income and low- and middle-income countries. - Bias, stigma and discrimination are in themselves considered public health threats (APA, 2020). Stigma is known to increase pre-existing differences faced by already disadvantaged or marginalised groups (IASC 2020, APA 2020), such as gender differences with women affected by the condition often facing double or triple ‘jeopardy’. Stigma-reduction efforts can thus be expected to have a positive effect on (health) equity. - Due to experiences of marginalisation, potentially stigmatized populations may distrust health authorities and resist cooperation during a public health emergency, accumulating different forms of stigmatization. This provides a further reason why considering stigma-reduction strategies in the broader context of health promotion (stigma in itself can also present a barrier to health-care seeking). - The social distancing approach and subsequent impact on people’s ability to work and earn money can in itself increase inequality and economic hardship – this can easily turn into a downward spiral. In TB the negative interaction between poverty, disease and stigma/ discrimination and their respective mutually reinforcing effects have been well documented. - Health protection relies not only on a well-functioning health systems with universal coverage, but also on social inclusion, justice, and solidarity. In the absence of these factors, inequalities are magnified and scapegoating persists, with discrimination remaining long after. Division and fear of others will lead to worse outcomes for all. - Rather than recognising complex long-term structural disadvantage and lack of opportunity that leaves certain groups more likely to have poor health (e.g. chronic conditions), there is a tendency to blame certain groups for behaviour associated with worse overall health (like smoking or obesity). Times of heightened fear may lead to media or population resorting to these stereotypes, implying individual fault for broader group characteristics. This form of ‘ecological fallacy’ has been seen in the response to reports of far higher rates of death from COVID-19 among African-American communities in the USA. |
| **Acceptability** | Is the intervention acceptable to key stakeholders?  ○ No ○ Probably no ○ Probably yes ○ Yes  **x Varies** ○ Don't know | - No evidence identified for this, but most reports on stigma in conditions sharing some similarities with COVID-19 indicate extensive acceptability of interventions to reduce stigma to a wide range of stakeholders - There is a strong public desire for clarity and transparency, and ability to distinguish good advice from false or misleading media reporting. Expert scientific advice regarding reducing stigma in relation to COVID-19 was highlighted as a key priority by an editorial in Nature (2020), signalling that evidence-based recommendations in this domain are welcomed. |
| **Feasibility** | Is the intervention feasible to implement?  ○ No ○ Probably no ○ Probably yes **x Yes**  ○ Varies ○ Don't know | - Interventions may not be similarly implemented in HIC and in LMIC. Most of the effective interventions require existing and functioning health care programs, and economic resources that are not equally distributed in the world. However, media-based interventions, including accurate information, careful use of terminology and engagement with population opinion leaders and champions can be used globally. Social media use is very high in many LMICs, but it needs to be recognised that access to online information has been politicised in some settings. - To be effective, behavioural intervention approaches, messages, and materials have to be salient for the affected population, so local contextualisation is needed. Therefore, the knowledge and perception regarding COVID-19, including local beliefs, fears, attitudes and behaviours should be investigated in settings where such factors are likely to vary. Similarly, interventions aiming at promoting an accurate understanding of the epidemic both in the general population and within the affected community, as well as the dynamic nature of the outbreak and its cause, treatment options, and prevention strategies would need extensive adaptation to local cultures and education levels. - It has also been outlined that steps must be taken to address stigma and discrimination at all phases of the COVID-19 emergency response (IASC, 2020), indicating that if longer-term resourcing is not available the impact of anti-stigma efforts might be compromised - Cultural/contextual variation may also exist in in the experience and manifestations of stigma, so interventions would need to take these aspects into due consideration. However, in relation to exploring mental health stigma-reduction strategies in LMICs, it was remarked the interventions had rarely been culturally/contextually adapted. - Different delivery modalities may adapt to different populations. For HIV-related stigma, for example, printed or movie-based material was shown to be key to deliver child-focused interventions, while professionals most commonly implemented adult-focused interventions (Hartog, 2020). - The highly contagious nature of COVID-19 and consequent responses of recommended self-isolation and public lock-downs, means that many responses have had to be adapted rapidly to ‘online’ or virtual versions (e.g. helplines rather than face-to-face contact). This means that evidence of efficacy of interventions (e.g. contact interventions for stigma reduction) have to take into account these adaptations. |

## GDG Recommendations

The recommendations presented below were developed by a review group, representing an interdisciplinary panel of international experts in the field of stigma and various infectious and/or highly stigmatised conditions, using standard WHO guideline development methodology. The recommendations were derived from data collated in the ‘Evidence to Decision’ table, summarising both evidence from systematic reviews, and insights from additional materials.

The recommendations are indicated as based on either “systematic reviews” (i.e. evidence from systematic reviews selected for inclusion in this review), or as based on “additional materials” (i.e. insights from commentaries, reviews and other papers, as identified by the search process and expert consultations, and determined as relevant for this review via expert consensus amongst the review group).

| **RECOMMENDATIONS** |
| --- |

**Overarching considerations**

The link between stigma and COVID-19, and its impact, should be considered not only for people who have a current confirmed or COVID-19, but also for people who have been released from COVID-19 quarantine or for people associated with COVID-19 due to their work (e.g. healthcare or social care workers), in relation to ethnicity or country of origin (because of public perceptions of groups among whom the virus is seen as more common, or who are to blame for its spread), or in relation to an affiliation with a person who is unwell or otherwise associated with the virus (e.g. caregivers, family members, peers).

Interventions to reduce stigma in relation with COVID-19 are recommended in all countries (High-, Middle-, and Lower-Income) as stigma is likely to manifest across cultures, and effective interventions to reduce it are likely to share similar basic characteristics.

All messages should be accessible to people of different ethnic and cultural groups, people with limited literacy, and people who require accessible formats due to disabilities. Inviting these groups to participate in planning and implementation of COVID-19 related activities improves the quality and effectiveness of such messaging.

Political exploitation of the pandemic needs to be avoided as it will lead to further stigma and discrimination and reduce the effectiveness of other efforts to combat COVID-19. The stigma of negative association with the outbreak for populations singled out in this way, is likely to endure past the current health crisis.

These recommendations are most effective if carried out together as part of a comprehensive response. That is, these recommendations are not intended as a list from which certain actions are selected, rather it is recommended that authorities actively consider implementing all of these recommendations where relevant, as an integrated approach to social inclusion and to individual, family and community recovery.

**1. Language and words†**

- 1. Language and words, which may have a negative meaning for people or reflect stigmatising attitudes, should not be used when talking about COVID-19. It is recommended: not to attach locations or ethnicity to the disease (not a Chinese Virus or Wuhan Virus), not to refer to people with COVID-19 as “cases” or “victims” or “suspects”; not to use exaggerated language or metaphors, such as “plague” or “apocalypse”; and not to use words that could imply intentional transmission and blame.
  2. Overemphasis of attribution of disease burden, severity and death to either ethnicity, pre-illness behaviour/travel history, age, gender or underlying medical conditions needs to be avoided as it devalues affected people, assigns blame to the victim and leads to a false sense of security in the rest of the population. These all undermine access to services and the epidemic control measures. (e.g. young people are more likely to break lock-down rules, as they have been made to believe that they are not in danger of serious complications)

**2. Media & Journalists†**

- 1. Language used in the media should be particularly considered (either as a delivery platform for anti-stigma strategies, or as a target for anti-stigma efforts to counter biased and stigmatised language), as media reporting can shape popular perceptions, discourse, communication, and behaviour.
  2. Mass media should be employed to share information focused on avoiding COVID-19 related stigma. The public must have easy access to balanced and accurate information from trusted sources, challenging inaccurate beliefs, and reducing prejudice against people affected by COVID-19 in the short, medium and long term. Sensationalist headlines and stories are to be avoided. Journalists have an ethical responsibility to understand and convey messages based on good science, and play an important role in emphasizing prevention practise, symptoms to look for and when and how to seek care.
  3. Communication training should be provided for those in government and in health/care services, including those providing public briefings
  4. Involve people affected by COVID-19, particularly using real-life based voices, stories and images of local people, including narratives related to recovery and hope. These are key factors for success in a range of anti-stigma interventions, and media can be used as a powerful platform for sharing such messages. Care should be taken to ensure consent and confidentiality when individual stories are reported.

**3. Public health interventions**

- 1. Stigma-reduction strategies in relation to COVID-19 targeting the general public should build on knowledge-shaping and attitude-changing strategies. *****
  2. Stigma in the general public can be reduced by providing treatment programmes for stigmatised conditions within general health care settings; this consideration is relevant in the treatment of possible long-term complications following COVID-19 (whereas during the acute phase best practices of infection/transmission control need to be observed, which may warrant special facilities and segregating procedures). *
  3. Mass media interventions targeting the general public (e.g. interventions delivered via newspapers, billboards, pamphlets, television, radio, cinema, or online) focusing on avoiding COVID-19 related stigma and on information are recommended to reduce prejudice for illness in the immediate, short and medium term. *
  4. Stigma-reduction campaigns for COVID-19 should include correcting myths, rumours and stereotypes, and challenging bias. Emphasis should go beyond factual knowledge, to strategically changing public perceptions about who is responsible for the outbreak, or addressing specific public misconceptions, which may vary among different populations and cultural/religious groups. In relation to infectious diseases, negative attributions are often based on fear. †
  5. Incorporating the arts and humanities into stigma-reduction approaches can stimulate social change, for example, by adopting a community arts collaborative approach to promote community engagement. Using ‘artists and art’ to showcase stories, conditions, and experiences of people who have suffered discrimination can help to cultivate engagement, empathy and acceptance. †
  6. To foster a sense of community and togetherness, rather than “otherness”, messages should emphasise the social responsibility of everyone to support efforts to reduce negative impacts of COVID-19. Focus should be on the positive outcomes achieved if everyone follows public heath instructions, which is more likely to succeed than actions directed by fear (e.g. enforcing compliance through media shaming, and/or emphasising negative outcome predictions). Positive messaging should also include praising and publicly supporting frontline workers and others who volunteer/assist vulnerable and in need populations (positive reinforcement/conditioning), and people from these communities themselves, who are often disproportionately represented among frontline workers. †
  7. Strategies to reduce and/or slow down transmission through avoiding physical contact should be framed as “physical distancing”, rather than “social distancing” which can exacerbate otherness and stigma, or “social isolation” which can increase the impact of stigma through limiting access to social support. Instead, remaining socially connected, facilitating communication, and promoting a sense of community, peer support, common purpose and inclusion for all is likely to improve solidarity and inclusion. †
  8. Stigma can be reduced through designing public health strategies for the whole population (e.g. universal access to COVID-19 testing, financial payments to facilitate economic security, health policies such as movement restrictions, physical distancing, surveillance mechanisms, travel bans and quarantine). This reduces discrimination compared to targeted strategies by avoiding implication of blame to particular individuals or groups. †
  9. Use of excessive policing or criminalising the breaching of COVID-19 related health policies is likely to increase stigma and discrimination, and risks loss of trust which may in turn reduce compliance with such measures, or lead to protests. †
  10. Comprehensive stigma mitigation requires public health strategies implemented in the immediate term (e.g. addressing misinformation and lack of awareness regarding COVID-19) to be complemented by efforts to tackle societal-level issues of social and economic inequalities that facilitate stigma in the long-term (e.g. racism, xenophobia, structural-level policies and laws). †

**4. Targeted public health interventions for key groups**

People directly affected by COVID-19

- 1. Stigma is likely to be a key concern for many people with direct experience of COVID-19, whether through a confirmed or suspected diagnosis, potentially during and after a period of illness. Stigma (anticipated, enacted, internalised) amongst persons affected by a stigmatised condition can be reduced through strategies building on group-based interventions, psycho-educational interventions, social empowerment strategies, community-based strategies (e.g. community support initiatives, improving community attitudes), and self-help interventions. *****

Family members

- 1. Stigma-by-association is also often experienced by family members of people affected by COVID-19. Interventions to mitigate this should build on close collaboration with communities, and positive, community-proposed coping strategies. Family-based interventions, and strategies based on empowerment, are also recommended to mitigate self-stigma amongst family members. †

Health care and frontline workers

- 1. Interventions to support and provide encouragement/counselling for those who are on the frontlines of response to COVID-19 (e.g. health and social care workers, volunteers, peer support workers) are strongly recommended. Furthermore, health care professionals and other key frontline workers need to be protected from discrimination and abuse related to their role in response to the outbreak, as this will increase their stress and work-pressures, and affects their ability to perform their roles. *
  2. An information-based approach, including the involvement of popular opinion leaders, should be implemented to reduce stigma against health workers. *

Vulnerable/high-risk populations

- 1. Protection from COVID-19 infection should be ensured for disadvantaged and marginalised groups (e.g. homeless people, people with disabilities, people who are incarcerated, migrants and refugees, and racial minorities), who may be particularly exposed to stigma and exclusion, and are at increased risk of COVID-19. Recommended stigma-related interventions should target, and be tailored to, these groups, who may also have health-related social inequalities. Care should be taken to ensure that targeted messaging does not inadvertently single out specific groups and lead to greater stigma and discrimination. †

1. **Involving communities and key stakeholders†**
   1. Involving persons affected by COVID-19, particularly using voices, stories and images of local people, is recommended as a key factor for success in a range of anti-stigma interventions.
   2. Anti-stigma messaging should be contextualised and targeted based on local knowledge of the specific beliefs and fears and drive stigma in a given setting, community, or target population, and if necessary rapidly deployed to minimise misinformation and fear at an early stage and as popular understanding changes.
   3. To understand stigma in a given context, it may be useful to scope information from local organisations, through discussions with community leaders, clinicians, analysing news messages, most accessed information on public health websites, and social media posts, for example by engaging with anthropologists and social scientists.
   4. Partnering with key community leaders (e.g. popular opinion influencers, religious and other trusted local leaders, peer role models) is essential to build trust, and develop and implement contextually appropriate strategies to address stigma. Respected community groups and leaders can provide valuable insight about stigmatisation and stigmatised groups. As de facto cultural translators, community leaders can help determine which methods and messages may reduce stigma and help organise community events designed to reduce stigma. They can draw on their interpersonal connections to promote reassurance and enhance community resiliency, add legitimacy to general public health efforts, and serve as a critical conduit to disseminate information to individuals and groups who might mistrust the government or other.

**Key**

*Recommendation is based on evidence from **systematic reviews** selected for inclusion in this review

†Recommendation is based on **additional materials** (i.e. insights from commentaries, reviews and other papers, as identified by the search process and expert consultations, and determined as relevant for this review via expert consensus of the review group)

# Appendix 1: Search strategy document

### Database searches

Searches will be conducted in:

PubMed/MEDLINE, PsycINFO, Cochrane Central and Campbell Collaboration

Search strategies are developed separately in relation to each condition, and each database.

Articles published in the English language will be included.

The search strategies are based on the following steps:

1. Search with limit to systematic reviews (and published in the last 10 years)

If this search yields systematic reviews relevant for inclusion, no further searches are ran for this condition.

If no systematic reviews on stigma-reduction in relation to the condition of interest are identified, the following step will be used

1. Search with limit to RCTs and clinical study and clinical trial and controlled clinical trial

If this search yields primary studies relevant for inclusion, no further searches are ran for this condition.

If no primary studies on stigma-reduction in relation to the condition of interest are identified, the following step will be used

1. Search without limits

This search is intended to identify other articles providing additional information reflecting guidance or commentaries on stigma-reduction in relation to the condition of interest.

Experts in infectious diseases and stigma will be consulted for further recommendations regarding relevant articles, and guidelines and recommendations from credited international and national public health organizations (e.g. CDC, WHO) and intra- and intergovernmental organizations responding to humanitarian crises and public health emergencies (e.g. UN).

### Search String

**COVID-19:**

((fear) OR (social stigma[MeSH Terms]) OR (stigma) OR (discrimination[MeSH Terms]) OR (discrimination)) AND ((COVID 19) OR (corona virus disease) OR (Covid-19) OR (2019-nCoV) OR (Novel coronavirus pneumonia) OR (NCP) OR (SARS-CoV-2) OR (wuhan coronavirus) OR (2019-ncov) OR (wuhan seafood market pneumonia virus) OR (2019 novel coronavirus))

**SARS:**

((social stigma[MeSH Terms]) OR (stigma) OR (discrimination[MeSH Terms]) OR (discrimination)) AND ((SARS) OR (sars coronavirus) OR (severe acute respiratory syndrome virus) OR (sars-related coronavirus) OR (sars related coronavirus) OR (sars-cov) OR (sars-associated coronavirus, urbani) OR (urbani sars associated coronavirus) OR (sars virus) OR (coronavirus, sars) OR (severe acute respiratory syndrome related coronavirus) OR (sars associated coronavirus') OR (coronavirus, sars[MeSH Terms]) OR (severe acute respiratory syndrome virus[MeSH Terms]))

**MERS:**

((social stigma[MeSH Terms]) OR (stigma) OR (discrimination[MeSH Terms]) OR (discrimination)) AND ((MERS) OR (middle east respiratory syndrome coronavirus) OR (mers-cov) OR (mers virus) OR (middle east respiratory syndrome related coronavirus') OR ("middle east respiratory syndrome coronavirus"[MeSH Terms]) OR ("middle east respiratory syndrome coronavirus/analysis"[MeSH Terms]))

**EBOLA:**

((social stigma[MeSH Terms]) OR (stigma) OR (discrimination[MeSH Terms]) OR (discrimination)) AND ((ebola) OR (ebola like virus[MeSH Terms]) OR (ebola hemorrhagic fever[MeSH Terms]) OR (evd))

#### INFLUENZA (H5N1/H1N1):

((social stigma[MeSH Terms]) OR (stigma) OR (discrimination[MeSH Terms]) OR (discrimination)) AND ((avian influenza[MeSH Terms]) OR (african swine fever virus[MeSH Terms]) OR (influenza) OR (H1N1) OR (H5N5) OR (bird flu))

#### TUBERCULOSIS:

((social stigma[MeSH Terms]) OR (stigma) OR (discrimination[MeSH Terms]) OR (discrimination)) AND ((tuberculosis) OR (pulmonary consumption) OR (lung tuberculosis) OR ("tuberculosis"[MeSH Terms]))

#### LEPROSY:

((leprosy) OR (leprosy[MeSH Terms])) AND ((social stigma[MeSH Terms]) OR (stigma) OR (discrimination[MeSH Terms]) OR (discrimination))

#### HIV:

((social stigma[MeSH Terms]) OR (stigma) OR (discrimination[MeSH Terms]) OR (discrimination)) AND ((HIV) OR (human immunodeficiency virus) OR (acquired immunodeficiency syndrome) OR (AIDS) OR (aids[MeSH Terms]) OR (acquired immune deficiency syndrome[MeSH Terms]))

#### MENTAL ILLNESS:

((social stigma[MeSH Terms]) OR (stigma) OR (discrimination[MeSH Terms]) OR (discrimination)) AND ((mental illness) OR (mental health) OR (psychiatric illness) OR ("mentally disabled persons"[MeSH Terms]) OR ("mental disorders"[MeSH Terms]) OR ("mental health"[MeSH Terms]) OR ("schizophrenia"[MeSH Terms]))

#### LIMITS

Validated study design filters within the databases were applied in the searches, as exemplified below.

1. Search with limit to systematic reviews

((fear) OR (social stigma[MeSH Terms]) OR (stigma) OR (discrimination[MeSH Terms]) OR (discrimination)) AND ((COVID 19) OR (corona virus disease) OR (Covid-19) OR (2019-nCoV) OR (Novel coronavirus pneumonia) OR (NCP) OR (SARS-CoV-2) OR (wuhan coronavirus) OR (2019-ncov) OR (wuhan seafood market pneumonia virus) OR (2019 novel coronavirus)) **Filters: Systematic Review** Sort by: Most Recent

1. Search with limit to RCTs and clinical study and clinical trial and controlled clinical trial

((fear) OR (social stigma[MeSH Terms]) OR (stigma) OR (discrimination[MeSH Terms]) OR (discrimination)) AND ((COVID 19) OR (corona virus disease) OR (Covid-19) OR (2019-nCoV) OR (Novel coronavirus pneumonia) OR (NCP) OR (SARS-CoV-2) OR (wuhan coronavirus) OR (2019-ncov) OR (wuhan seafood market pneumonia virus) OR (2019 novel coronavirus**)) Filters: Clinical study, Clinical Trial, Controlled Clinical Trial, Randomized Controlled Trial** Sort by: Most Recent

**COMMENT:** During the screening of results, a post hoc specification was added to include articles focused on “mental illness” in a general sense, and to exclude articles focused on severe mental illness specifically. This specification was decided on upon noticing that a number of systematic reviews focused on stigma in relation to severe mental illness, but the potentially specific nature of stigma related to severe mental illness might not provide a useful point of comparison with stigma in relation to COVID-19. Also, given how much anti-stigma research is conducted in relation to mental illnesses, this restriction provided a rationale for limiting the volume of mental illness-related evidence that was considered for this review, to ensure a more balanced set of evidence from across conditions could be included. There were no further deviations from the predefined review protocol.

### Example of detailed search strategy

- Condition: SARS
- Database: PUBMED
- Date: April 2020

The search strategies are based on the following steps:

**Step 1) Search with limit to systematic reviews (and published in the last 10 years)**

((social stigma[MeSH Terms]) OR (stigma) OR (discrimination[MeSH Terms]) OR (discrimination)) AND ((SARS) OR (sars coronavirus) OR (severe acute respiratory syndrome virus) OR (sars-related coronavirus) OR (sars related coronavirus) OR (sars-cov) OR (sars-associated coronavirus, urbani) OR (urbani sars associated coronavirus) OR (sars virus) OR (coronavirus, sars) OR (severe acute respiratory syndrome related coronavirus) OR (sars associated coronavirus') OR (coronavirus, sars[MeSH Terms]) OR (severe acute respiratory syndrome virus[MeSH Terms])) Filters: Systematic Review in the last 10 years

3 results (all excluded)

**Step 2) Search with limit to RCTs and clinical study and clinical trial and controlled clinical trial**

((social stigma[MeSH Terms]) OR (stigma) OR (discrimination[MeSH Terms]) OR (discrimination)) AND ((SARS) OR (sars coronavirus) OR (severe acute respiratory syndrome virus) OR (sars-related coronavirus) OR (sars related coronavirus) OR (sars-cov) OR (sars-associated coronavirus, urbani) OR (urbani sars associated coronavirus) OR (sars virus) OR (coronavirus, sars) OR (severe acute respiratory syndrome related coronavirus) OR (sars associated coronavirus') OR (coronavirus, sars[MeSH Terms]) OR (severe acute respiratory syndrome virus[MeSH Terms])) Filters: Clinical Trial, Controlled Clinical Trial, Randomized Controlled Trial

5 results (all excluded)

**Step 3) Search without limits**

((social stigma[MeSH Terms]) OR (stigma) OR (discrimination[MeSH Terms]) OR (discrimination)) AND ((SARS) OR (sars coronavirus) OR (severe acute respiratory syndrome virus) OR (sars-related coronavirus) OR (sars related coronavirus) OR (sars-cov) OR (sars-associated coronavirus, urbani) OR (urbani sars associated coronavirus) OR (sars virus) OR (coronavirus, sars) OR (severe acute respiratory syndrome related coronavirus) OR (sars associated coronavirus') OR (coronavirus, sars[MeSH Terms]) OR (severe acute respiratory syndrome virus[MeSH Terms]))

136 results
